# Dual-targeting phytochemicals Ergosterol and Quercetagetin implicate steroid metabolism–associated pathways in lung and liver cancer models

**DOI:** 10.3389/fphar.2026.1773130

**Published:** 2026-05-01

**Authors:** Yujiao Chen, Yuqian Wu, Madineh Moradialvand, Omar Eladl, Simiao Du, Shanshan Yang, Xiaomin Liu, Shujun Zhang, Jianqing Chen, Jingwen Zeng, Xiaowei Su, Hong Ma, Hong Lu, Jianhua Feng, Jun Cao, Li Zhong, Guixue Wang, Jun Yang, Hassan Karimi-Maleh, Pooyan Makvandi

**Affiliations:** 1 Zhejiang University School of Medicine, Hangzhou, China; 2 Department of Physiology, Department of Cardiology of the Second Affiliated Hospital and School of Basic Medical Sciences and State Key Laboratory of Transvascular Implantation Devices and Jade Biotechnology, Zhejiang University, Hangzhou, China; 3 Key Laboratory for Biorheological Science and Technology of Ministry of Education, National Local Joint Engineering Laboratory for Vascular Implants, Bioengineering College of Chongqing University, Chongqing, China; 4 Guizhou Gui’an Academy of Precision Medicine, Gui'an, Guizhou, China; 5 Institute of Panvascular Biology, JinFeng Laboratory, Chongqing, China; 6 The Quzhou Affiliated Hospital of Wenzhou Medical University, Quzhou People’s Hospital, Quzhou, Zhejiang, China; 7 Faculty of Pharmacy, Egypt-Japan University of Science and Technology (E-JUST), Alexandria, Egypt; 8 Zheng YuanTang (Tianjin) Biotechnology, Tianjin, China; 9 School of Basic Medical Sciences, Southwest Medical University, Luzhou, Sichuan, China; 10 College of Life Sciences and Medicine, Zhejiang Provincial Key Laboratory of Silkworm Bioreactor and Biomedicine, Zhejiang Sci-Tech University, Hangzhou, China; 11 Guizhou Aerospace Intelligent Agriculture, Guiyang, Guizhou, China; 12 Tianjin Lakeside Powergene Science Development, Tianjin, China; 13 School of Chemistry, Damghan University, Damghan, Iran; 14 University College, Korea University, Seoul, Republic of Korea; 15 Centre for Research Impact and Outcome, Chitkara University, Rajpura, Punjab, India

**Keywords:** androgen receptor, ergosterol, estrogen receptor 1, hormone associated cancer, immunometabolism, nanocarrier, quercetagetin

## Abstract

**Introduction:**

Hormone-associated cancers use steroid metabolism to grow and avoid the immune system, while traditional treatments have to deal with drug resistance and systemic toxicity.

**Methods:**

This study is mainly divided into two parts. First, screening natural active ingredients for anti-lung cancer and anti-liver cancer and their network pharmacology research. Second, target identification and clinical drug design of potential medicinal components focusing on hormone metabolic pathways.

**Results and Discussion:**

We found that ergosterol and quercetagetin are dual-targeting phytochemicals that interfered with hormone metabolism in a potential contributing pathway through androgen receptor (AR) and estrogen receptor 1 (ESR1) and enzymes such as 3*β*HSD/17*β*HSD using network pharmacology, molecular docking, and SMRT sequencing. We used HepG2 and A549 cell lines to do mechanistic studies. Our *in vivo* results were confirmed by mouse models of Lewis lung carcinoma and H22 hepatoma. Ergosterol may stop 3*β*-hydroxysteroid dehydrogenase (3*β*HSD) from working, which stop the conversion of DHEA to androstenedione in HepG2 cells. Quercetagetin may affect 17*β*-hydroxysteroid dehydrogenase (17*β*HSD), which throw off the balance of estradiol and estrone in A549 cells. *In vivo* studies showed that quercetagetin significantly stopped splenomegaly and thymic atrophy (p < 0.001). Pharmacokinetic analysis showed that 8 out of 31 compounds met Lipinski’s criteria. ADMET testing showed that ergosterol had poor solubility (Log S = −6.91) and fully bound to plasma proteins. Quercetagetin didn't pass through membranes well and was sensitive to P-glycoprotein efflux. Tailored nanocarrier systems showed significant improvements in overcoming these limitations: Ergosterol’s solubility increased by 1,000 times, and quercetagetin’s P-gp substrate status was removed. Molecular dynamics simulations showed that drug-nanocarrier interactions were stable, with ergosterol showing very little change (RMSD <0.1 nm). Overall, these findings support a working mechanistic hypothesis and motivate nanodelivery strategies to improve the developability of these phytochemicals for further preclinical evaluation.

## Graphical Abstract

**Figure:**
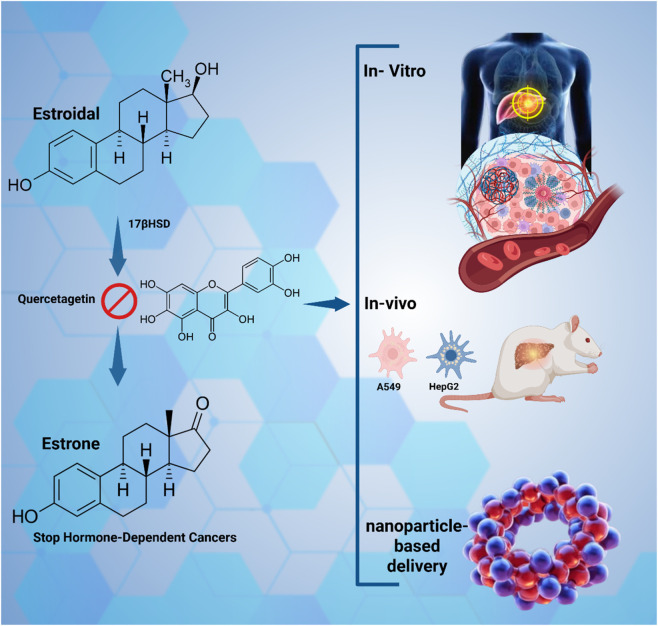

## Highlights

- First-time ID of AR, ESR1, and enzymes 3*β*HSD/17*β*HSD via SMRT & network pharmacology.
- Ergosterol (*C. militaris*) was predicted to engage AR/3*β*HSD-associated pathways and was associated with reduced DHEA→androstenedione conversion in liver cancer cells.
- Quercetagetin (*T. erecta*) was predicted to engage ESR1/17*β*HSD-associated pathways and was associated with altered estradiol/estrone balance in lung cancer cells.
- 31 compounds screened; Hormone-like ergosterol & quercetagetin validated as anti-cancer potential compounds.
- Ergosterol and quercetagetin attenuate tumor-induced splenomegaly and thymic atrophy in murine models.

## Introduction

Contemporary cancer therapeutics heavily depend on synthetic or semi-synthetic agents, such as abiraterone acetate, trabectedin, and immune modulators like lenalidomide, which target specific molecular pathways to disrupt tumor progression. These drugs operate through distinct mechanisms: single-target inhibitors (e.g., erlotinib) (Jensen, 2016), dual-target agents (e.g., lapatinib) (Xu et al., 2021), multi-target kinase inhibitors (e.g., pazopanib) (Choi et al., 2022) and immune modulators (e.g., mifamurtide) (Bastea et al., 2019). However, their clinical utility is often hampered by toxicity, drug resistance, and narrow mechanistic scope, underscoring the urgent need for safer, multi-faceted alternatives.

**SCHEME 1:**
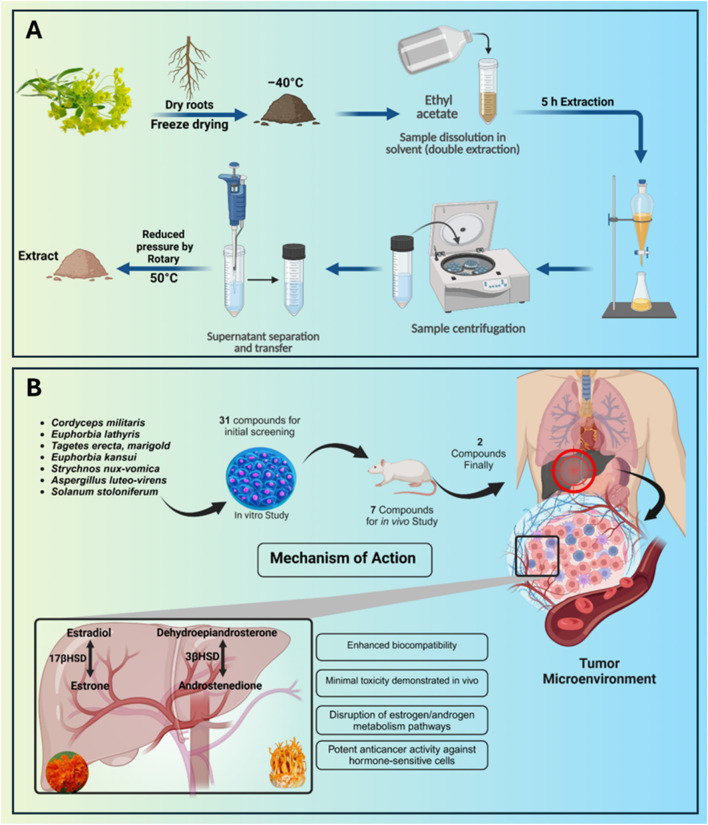
Schematic illustration of key enzymes in hormone metabolism and extraction and concentration methodology. **(A)** Ultrafine grinding at −40 °C followed by ethyl acetate extraction and rotary evaporation concentration for bioactive compound preservation. anticancer drugs natural substances. **(B)** Disrupting hormone metabolism pathways critical for cancer progression.

Natural products, historically pivotal in treating complex diseases, offer a promising yet underexplored resource. Artemisinin (malaria) and arsenic trioxide (leukemia) exemplify their therapeutic potential (Shen et al., 1997; Zhao et al., 2020), while traditional systems like the *Compendium of Materia Medica* document over 1892 natural medicines, including 54+ monomeric anti-cancer compounds (e.g., ginsenoside Rh2, cordycepin) and 60+ bioactive extracts; Despite this legacy, the functional components of many natural substances remain poorly characterized, particularly their mechanisms of action in hormone metabolism—a critical axis in cancers driven by steroid signaling (Luo et al., 2019).

To address this gap, we systematically investigated seven natural sources (*C. militaris*, *T. erecta*, *Euphorbia* spp., etc.) for anti-cancer compounds targeting hormone pathways. Prior studies identified ergosterol from *C. militaris* as a suppressor of lung (A549) and liver (HepG2) tumor growth (Chen Y. Y. et al., 2021), while *T. erecta*-derived quercetagetin and *Euphorbia* alkaloids demonstrated selective cytotoxicity (Sun et al., 2025). However, the molecular targets and pathways governing their efficacy, particularly in steroid hormone regulation, remain unresolved. Leveraging Single Molecule Real-Time (SMRT) sequencing, network pharmacology, and molecular docking, this study investigates the involvement of 3*β*-hydroxysteroid dehydrogenase (3*β*HSD), 17*β*-hydroxysteroid dehydrogenase (17*β*HSD), and their relationship to androgen receptor (AR) and estrogen receptor 1 (ESR1) in mediating the anti-cancer effects of ergosterol and quercetagetin and proposes a testable mechanistic framework for future validation. These findings bridge critical mechanistic gaps in natural product research, positioning phytochemicals as viable candidates for functional foods and targeted therapies against hormone-driven malignancies.

Based on these considerations, we hypothesized that selected sterol- and flavonoid-derived natural compounds exert anti-cancer activity through coordinated modulation of steroidogenic enzymes (3*β*HSD and 17*β*HSD) and dual regulation of AR and ESR1 signaling pathways. We further propose that simultaneous disruption of steroid hormone biosynthesis and receptor activation represents a unified mechanistic strategy to inhibit hormone-associated tumor progression (Schloms and Swart, 2014; Cassetta et al., 2017).

Notably, several identified potential compounds, including ergosterol and quercetagetin, exhibit pharmacokinetic limitations such as poor aqueous solubility, strong plasma protein binding, and potential P-glycoprotein (P-gp)-mediated efflux, which can reduce systemic bioavailability and tumor exposure. To overcome these limitations and improve delivery efficiency, we incorporated a computational nanocarrier modeling strategy to evaluate whether nanoconfinement could enhance structural stability, interaction behavior, and translational potential of these phytochemicals (Batsukh and Tsend-Ayush, 2024; Shivali et al., 2026).

Beside dual-targeting properties demonstrated by ergosterol and quercetagetin show remarkable promise for the formulation of next-generation nanodrug delivery systems to transform hormone-associated cancer treatment. The versatility of nanocarrier platforms, such as liposomes, polymeric nanoparticles, and stimuli-responsive nanosystems, can capitalize on the EPR effect to facilitate targeted tumor accumulation and also offer sustained release of these phytochemicals. Besides, the dual control of steroid metabolism and immune response render these molecules ideal candidates for multifunctional nanodelivery systems. Such systems can potentially facilitate co-delivery of immunotherapeutic agents and bypass the limitations of traditional single-target approaches. This delivery approach, ushered in by advancements in nanotechnology, has the potential to improve bioavailability, reduce systemic toxicity, and facilitate personalized dosing regimens, effectively marrying phytochemical pharmacology to precision oncology.

Biomimetic nano-drug preparation is a new direction for the development of anticancer drugs with high efficiency and low toxicity. A disorder of cholesterol homeostasis is one of the main initiating factors in the progress of atherosclerosis (Zhu et al., 2023), In the early stage, we have accumulated a relatively mature technical system in the targeted delivery system of biomimetic nano-drugs (Li et al., 2024), and the further developed drugs of Atherosclerosis and Ischemic-Related Disease have shown remarkable safety and efficacy (Zeng et al., 2025), which provides a solid scientific basis for the development of biomimetic nano-drug preparations of hormone-like compounds.

## Materials and methods

The overall methodology of this study is primarily divided into two parts (Scheme 1):

1. Screening natural active ingredients for anti-Lung cancer and anti-liver cancer and their network pharmacology research

A) Natural compounds were extracted via solvent extraction and purified using industrial chromatography, identifying active components with significant inhibitory effects on the proliferation of A549 cells (lung cancer model cell) and HepG2 cells (liver cancer model cell). Existing databases on lung and liver cancer drug targets were further compiled to predict potential targets and pathways of these active compounds through network pharmacology methods, providing more references for natural active compounds in cancer drug development. B) Compounds meeting the required sample volume for animal experiments were further selected to conduct *in vivo* efficacy studies (as all the compounds in this study were extracted and isolated from natural raw materials, the trace active ingredients may not necessarily meet the needs of animal experiments. Therefore, only sufficient amounts of active compounds can be selected for animal experiments).

However, due to the substantial workload, this study will focus on screening 1-2 active compounds for proposed anticancer mechanisms and drug design, while the remaining active compounds will be developed progressively in the future.

2. Target identification and clinical drug design of potential medicinal components focusing on hormone metabolic pathways

A) Using A549 and HepG2 cells as model cell lines, we conducted whole transcriptome sequencing via SMRT sequencing to identify mutated enzymes. By integrating literature data, we mapped human hormone metabolites and their corresponding catalytic enzymes, further pinpointing the hormone metabolic catalytic enzymes that had undergone mutations in A549 and HepG2 cells. B) Screening natural active compounds with hormone-like properties to further design clinically efficient and low-toxicity nanomedicines (since hormone-like active components may possess regulatory tendencies toward hormone metabolism).

The detailed method process is as follows:

### Materials of 7 kinds of natural substances

*C. militaris* HN fruiting bodies were provided by Jade Biotechnology & Guizhou Gui’an Precision Medicine Co. Ltd., Gui’an new area, Co., Ltd. *Armillaria luteo-virens* (Aalb.et Schw: Fr.) Sacc. was purchased from Qilian, Qinghai, China. *Euphorbia lathyris* L., *Euphorbia kansui* T. N. Liou ex T. P. Wang, *Strychnos nux-vomica* L., and *Sparganium stoloniferum* Buch.-Ham. were purchased from Anguo, Hebei, China. *Tagetes erecta* L. was provided by Chenguang Biotech Group Co., Ltd. Handan, Hebei, China.

### Materials in extraction and separation

Methanol, acetone, n-hexane, ethyl acetate, dichloromethane, acetonitrile, and ethanol (chromatographic grade) were purchased from Tianjin Concord Technology Co., Ltd. Ultrapure water, Milli-Q level, was self-made in our laboratory (κ = 18.2 MΩ·cm). Dynamic axial columns (DAC) (600 × 250 mm C18 10 µm), DAC (150 × 250 mm silica 10 µm), and DAC (50 × 250 mm Hilic 10 µm) were purchased from Jiangsu Hanbang Technology Co., Ltd. DAC (50 × 250 mm silica 10 µm) and DAC (21.2 × 250 mm silica 10 µm) were purchased from Tianjin Bonaageer Technology Co., Ltd. Kromasil (250 mm × 4.6 mm silica 5 µm 100 Å), Kromasil (250 mm × 4.6 mm silica 5 µm 100 Å), and Kromasil (250 mm × 4.6 mm Hilic 5 µm 100 Å) were purchased from Beijing Zhenxiang Industry and Trade Co., Ltd. Xcharge (4.6 × 250 mm C18 5 μm 100 Å) was purchased from DAISO Co., Ltd., Japan.

### Materials in cells experiments

HepG2 and A549 cells were provided by Shanghai Institute of Biochemical Cells, Chinese Academy of Sciences. Dulbecco’s modified eagle medium (DMEM) serum-free cell culture medium was purchased from Kilton Biotechnology Shanghai Co., Ltd. Fetal bovine serum (FBS) and pancreas protease were purchased from Invitrogen. Penicillin, streptomycin and roswell park memorial institute (RPMI) 1,640 medium were purchased from Life Technologies Corporation.

### Materials in mice experiments

Lewis lung cancer cell lines (6th generation), H22 liver cancer cell lines (4th generation), and cyclophosphamide tablets were purchased from Tianjin Jinshi Pharmaceutical Co., Ltd. Tween 80 was purchased from Beijing Borun Wright Technology Co., Ltd. 1) Materials in Lewis and H22 tumor mice experiments. C57BL/6 and Kunming mice (18–22 g, animal license number: SCXK (Beijing), certificate number: 11400700020779) were purchased from Beijing Weitong Lihua Laboratory Animal Technology Co., Ltd. 2) Materials in acute toxicity mice experiments.Kunming mice (18–25 g, half male and half female. animal license number: SCXK (Jun), certificate number: 11400700020779) and feed (SCXK (Jun) were purchased from the Experimental Animal Center of the Chinese Academy of Military Medical Sciences. The breeding environment of these mice was normal grade and 21 °C (room temperature).

### Extracting and separating compounds from natural substances

The extraction and separation methods of *C. militaris* HN fruiting body, *E. lathyris*, *T. erecta*, and *E. kansui* were performed as described previously (Chen Y. et al., 2021). And the comprehensive extraction and separation method, refer to our authorized Chinese patent (No. ZL202221634792.4).

### Inhibition HepG2 and A549 growth experiments *in vitro*

The xCELLigence system (the sensor impedance technology) was used to detect the change in the electronic impedance sensor. These changes could reflect the state of cell growth.

The test compound was dissolved in dimethylsulfoxide (DMSO), and HepG2/A549 cells were cultured in DMEM/RPMI 1640 (10% FBS +1% penicillin-streptomycin) medium under 5% CO_2_ at 37 °C. Cells in the logarithmic phase were selected and diluted with a culture medium.

A real-time monitor was placed in a saturated humidity incubator (5% CO_2_, 37 °C). First, 150 µL of DMEM/RPMI 1640 medium was added to each well of an 8-well plate. Second, the plate was placed on the monitor to obtain the baseline. Third, after removing the plate, the diluted HepG2/A549 cells suspension of about 4 × 10^4^/2 × 10^4^ cells per well was added. Fourth, the compound was added to the experimental well at a final concentration of 50 μg·mL^-1^ and 1% DMSO was added to the control well. Finally, the plate was placed on the monitor for testing.

### Drug-like analysis of anticancer compounds

The SMILES IDs of active compounds were obtained from the PubChem database (https://pubchem.ncbi.nlm.nih.gov/), and their 3D structures were saved in the mol2 format (Kim et al., 2021). Druglikeness was determined using Lipinski’s rule of 5 (Ro5) and calculated by the Molispiration website (https://www.molinspiration.com/cgi-bin/properties), which specifically included the relative molecular weight (MW), hydrogen bond acceptor number (HBAN), hydrogen bond donor number (HBDN), lipid–water partition coefficient (log P), and the number of rotatable bonds (NROTB).

Substances that comply with Ro5 parameters have better pharmacokinetic properties, higher bioavailability, and can be easily administered orally. MW was less than 500, HBDN was less than 5, HBAN was less than 10, the lipid–water partition coefficient was less than 5, and NROTB was not greater than 10.

### Collection the compound-target network

The target of compounds was predicted by the Swiss Target Prediction platform (Daina et al., 2019). The top 15 targets or targets with a probability greater than 0.5 were selected as positive targets, and the compound-target network was constructed using Cytoscape 3.7.2. In the network, a node represented compounds and targets, and an edge represented the relationship between compounds and their targets. The compound-target network characteristics and potential hub targets were determined using the network analyzer plug-in Cytoscape.

### Collection targets associated with liver and lung cancer

We searched for disease targets in the GeneCard database (https://www.genecards.org/), GenCLiP 3 text mining Tools (http://ci.smu.edu.cn/genclip3/analysis.php), and DisGeNET v7.0 database (https://www.disgenet.org/) using the keywords “lung cancer”, “lung carcinomas”, and “hepatocellular carcinomas” (Wang et al., 2019). Second, duplicate targets were removed after matching with the obtained potential targets. Third, the targets of anti-lung cancer and anti-liver cancer were obtained from the Venn diagram intersection using the “VennDiagram” data package in the R language.

### Building the protein-protein interaction (PPI) network

Based on the obtained anti-lung cancer and anti-liver cancer targets, PPI network analysis was performed. Targets were input into the String 11.0 online database by the gene symbol (Piñero et al., 2020). Second, the target interaction relationship was obtained, saved in the tsv file format, and imported into Cytoscape (“*Homo sapiens*” was selected as the species to ensure high confidence, and the minimum required interaction score was set to “high confidence >0.7”) (Shannon et al., 2003). Third, the topological properties of the intersection target were analyzed using the CytoNCA plug-in (Tang et al., 2015), in which the higher the quantitative value of the central character of the node in the network, the more important the node was in the network, and specifically, degree centrality (DC), betweenness centrality (BC), closeness centrality (CC), and eigenvector centrality (EC). Finally, targets in which four central characteristics were all greater than the median (centrality quantitative values) were selected as key targets in the PPI network. The PPI network diagram of key targets was constructed according to the results.

### Analysis of medicinal targets and pathways

The enriched Kyoto Encyclopedia of Genes and Genomes (KEGG) pathways of the top 20 genes were analyzed. The pathways screened and enriched were related to anti-liver cancer and anti-lung cancer. Finally, the targets were paired with the active compounds, and Cytoscape was used to draw the compound–target network relationship diagram.

The key targets of Gene Ontology (GO) and KEGG pathways were analyzed by the ClusterProfiler package in R language (Yu et al., 2012). The results were visualized using the ggplot2 package. Specifically, the GO enrichment bar graph showed the top 10 biological processes, cellular components, and molecular functions. KEGG enrichment analysis used the first calculation of the *P*-value of each enrichment pathway of the target set (the Bonferroni method was used for correction, *P* < 0.01 was considered significant enrichment). Finally, the KEGG enrichment bubbles were outputted.

### Molecular docking of compounds with their targets

The binding energy of the compound with key targets was verified using AutoDock Vina 1.1.2. First, the 3D structures of the compounds were downloaded from the PubChem database, and the crystal structures of the key targets were obtained from the RCSB database (http://www.rcsb.org/). Second, water molecules and small ligand molecules were removed, and the protein structure as the receptor for docking was separated, and pretreated by AutoDockTools, such as hydrogenation, and both compounds and targets were converted to the pdbqt file format. Lastly, compounds were docked with their targets by AutoDock Vina. The lowest binding energy data (output) was the result of molecular docking. The lower the binding energy, the stronger the binding between compounds and target proteins. When the binding energy of the compound with the target is ≤ -5.0 KJ·mol^-1^, the binding is better (Trott and Olson, 2010).

### Screening high-medicine compounds by H22 and Lewis *in vivo*

The H22/Lewis lung cancer animal model establishment, drug preparation, and observation factors were as follows. (1) General status statistics: the body weight and diet of mice were recorded daily, and the net weight was calculated after tumor tissue removal on the last day (i.e., day 15). (2) Tumor inhibition rate: the right armpit of mice was observed daily. After 24 h on the last day (mice were administered medicine), the tumor tissue was taken out and weighed, and the tumor inhibition rate was calculated according to the formula: tumor inhibition rate = (tumor weight of the model group − tumor weight of the therapy group)/tumor weight of the model group × 100%. (3) Liver/lung tissue weight and cancerous lung/liver metastasis rate: after 24 h on the last day (mice were administered medicine), the liver/lung tissues were taken out, weighed and observed. Finally, the mice with cancer-metastasis lung (Supplementary Table S2) and liver (Supplementary Table S3) were counted.

The selected dose was chosen based on tolerability observed in the pilot dose-escalation experiment. For example, used for euphorbia factor L1, the *in vivo* dose of 54 mg·kg^-1^ was determined using a preliminary pilot toxicity study conducted in our laboratory. Briefly, cohorts of mice received escalating doses of 25, 50, 75, and 100 mg·kg^-1^, and were monitored for 48 h for signs of acute toxicity (including piloerection and reduced motility). The 75 mg·kg^-1^ dose produced visible toxicity within 48 h, whereas 50 mg·kg^-1^ was well tolerated with no significant behavioral or physiological abnormalities. Therefore, 54 mg·kg^-1^ was selected as a high yet tolerable dose for the main *in vivo* efficacy experiment.

### Analysis of the enzymes’ sequence variants in hormone metabolism by transcriptome SMRT sequencing of HepG2 and A549 cells

Total RNA was isolated using the UNIQ-10 column TRIzol total RNA extraction kit (Sangon Biotech) according to the manufacturer’s instructions, followed by treatment with DNase I. The mRNA was purified by poly-T column separation and stored at −80 °C until further analysis. The Iso-Seq library was prepared according to the PacBio Isoform Sequencing protocol (Iso-Seq™). RNA was reverse transcribed using the SMARTer PCR cDNA Synthesis Kit and was PCR amplified using KAPA HiFi PCR kits. These cDNA products were purified using the SMRTbell DNA Template Prep Kit 3.0 for library construction. Libraries were sequenced using P6C4 polymerase and chemistry on a PacBio RS II platform with 240 min movie times.

The standard RS_IsoSeq.1 protocol (SMRT Analysis 2.3.0p5) was used to process the raw sequence data. In brief, the ROI lines were generated and separated into full-length and non-full-length ROIs using “pbtranscript.py classify”. The full-length ROIs were clustered and assembled into consensus sequences by performing isoform-level clustering using an ICE algorithm with estimated cDNA sizes of 1–2 kb. Subsequently, the consensus sequences were polished based on the non-full-length ROIs and categorized as high-quality (above 99% accuracy) or low-quality full-length polished consensus transcripts using Quiver. The sequences of the transcriptome assembly were compared with 5,000 normal enzymes from NCBI, and screening out metabolic enzymes gene mutation in A549 and HepG2 cells by our technology (the metabolic enzyme gene mutation screening method used in this study have been granted Chinese invention patents, with authorization number ZL2021116752639).

### ADMET and physicochemical prediction

To assess the pharmacokinetic properties and drug-likeness of free and nanocarrier-conjugated drug molecules, SwissADME and pkCSM web-based platforms were used. Molecular structures of ergosterol and quercetagetin, both in their native and nanoconjugated forms, were submitted as SMILES inputs. SwissADME predictions included physicochemical descriptors such as molecular weight, lipophilicity (Log P), solubility (Log S), total polar surface area (TPSA), fraction of sp^3^ carbons (Csp^3^), and drug-likeness rule compliance (Lipinski, Ghose, Veber, Egan, and Muegge). pkCSM was used to evaluate absorption, distribution, metabolism, excretion, and toxicity (ADMET) profiles. Parameters analyzed included Caco-2 permeability, intestinal absorption, plasma protein binding, volume of distribution, cytochrome P450 enzyme interactions, total clearance, and potential toxicities such as hERG inhibition, mutagenicity, and hepatotoxicity. These predictions supported the rational design of nanocarrier systems by quantifying improvements in pharmacokinetic profiles after conjugation, guiding the evaluation of drug delivery efficiency and therapeutic viability.

### Molecular dynamics simulations

To evaluate the structural stability and interaction behavior of drug–nanocarrier systems, molecular dynamics (MD) simulations were performed using the GROMACS simulation package. All bonding and non-bonding interactions were modeled using the CHARMM36 all-atom force field. Drug topologies were generated via the SwissParam online server, while the carbon nanotube (CNT) topology was created using the x2top module and CHARMM-GUI server integrated with GROMACS tools.

Carbon nanotubes were employed in this study as a computational nanocarrier as a proof-of-concept *in silico* platform due to their well-defined, rigid, and π-conjugated surface, which enables efficient modeling of hydrophobic and π–π stacking interactions with sterol- and flavonoid-like phytochemicals identified in this work. Their structural simplicity and stability make them particularly suitable for reproducible molecular dynamics simulations, allowing controlled evaluation of adsorption behavior and interaction energies (Rezazade et al., 2024; Zhang et al., 2017; Yuan et al., 2019).

Each system was solvated in a cubic box using TIP3P water molecules, and neutralized by adding the appropriate counterions. Energy minimization was performed using the steepest descent algorithm, followed by two-step equilibration: first under constant volume and temperature (NVT) using a Berendsen thermostat at 310 K, then under constant pressure and temperature (NPT) using a Berendsen barostat at 1 bar for 1 nanosecond.

All bond lengths were constrained using the LINear Constraint Solver (LINCS) algorithm, and long-range electrostatic interactions were computed with the Particle Mesh Ewald (PME) method. A cutoff distance of 1.2 nm was applied for short-range van der Waals interactions. After equilibration, a 100-nanosecond production MD simulation was conducted for each drug–nanocarrier complex to monitor dynamic interactions and stability.

### Statistical analysis

R 4.0.3 was used for statistical analysis, and the experimental results were expressed as (x ± SEM), and differences between groups were compared by student’s *t*-test. The SPSS 19.0 software (IBM Corp., Armonk, NY, United States) was used for statistical analysis, and the measurement data were expressed as (x ± s). One-way analysis of variance was used for comparison between multiple groups. For comparison between groups, the Wilcoxon rank sum test was used (not in line with normality). For the comparison of means between multiple groups, the Dunnett T3 test was used (when the variances were not uniform). Values are expressed as means ± SD. *P* < 0.05 was considered statistically significant, and *P* < 0.01 was considered highly significant.

## Results

*In vitro* and *in vivo* cytotoxic activity: At a concentration of 50 μg·mL^-1^, all 31 tested compounds inhibited the growth of HepG2 cells, and 22 of them were active against A549 cells. Among the seven compounds selected for *in vivo* evaluation, ergosterol exhibited the strongest antitumor effect in the H22 hepatoma model. Notably, quercetagetin demonstrated comparable efficacy in both the Lewis lung carcinoma and H22 hepatoma models. No other compound showed such dual-model *in vivo* activity, making these two candidates uniquely promising for translational development.

Network pharmacology centrality toward AR and ESR1: Within the compound–target–pathway network (108 nodes, 781 edges), both ergosterol (compound 1, degree = 6) and quercetagetin (compound 16, degree = 5) showed direct connectivity to AR (degree = 28) and ESR1 (degree = 28)—two steroid hormone receptors that both ranked among the top 14 hub targets. Importantly, no other compound in the active pool exhibited predicted connectivity to both steroidogenic receptor targets simultaneously.

Molecular docking affinity toward hormone-metabolism targets: Molecular docking revealed the affinity of the compounds for hormone-metabolism targets. Ergosterol (compound 1) exhibited binding energies of −9.2 kcal·mol^-1^ toward EGFR and −11.4 kcal·mol^-1^ toward AKT1, and was also predicted to dock into the steroid-binding domain of AR. The predicted affinity of quercetagetin (compound 16) for ESR1 is consistent with literature reports on the interaction of flavonoids with nuclear receptors. Importantly, both compounds demonstrated favorable binding geometry within the ligand-binding pockets of their primary targets. These *in silico* findings were built upon prior SMRT sequencing data, which confirmed the transcriptional expression of hormone-metabolism-related genes in HepG2 and A549 cells and revealed sequence variants in the corresponding enzymes (3*β*HSD in HepG2; 17*β*HSD in A549).

Hormone-like structural pharmacophore: Of the 31 active compounds, only ergosterol has a steroidal tetracyclic carbon skeleton that is similar to endogenous androgens, and only quercetagetin has a polyhydroxylated flavonoid scaffold with well-known phytoestrogenic properties. This structural hormone-mimicry offered a mechanistically coherent and distinctive justification for the selection of these two compounds to examine steroid-metabolism-associated pathways, which forms the principal hypothesis of the study.

To summarize: Ergosterol (1) and quercetagetin (16) were chosen as the best candidates for further mechanistic research among the 31 active compounds because they met four different criteria. First, both compounds showed consistent anti-tumor activity in two murine cancer models. Ergosterol had the strongest tumor inhibition in the H22 hepatoma model, and quercetagetin had significant efficacy in both the Lewis lung carcinoma and H22 hepatoma models. No other compound in the active pool had a dual-model profile like these two. Second, in the compound–target–pathway network, ergosterol (degree = 6) and quercetagetin (degree = 5) were the only compounds among the 31 candidates that were predicted to connect to both the androgen receptor (AR; degree = 28) and the estrogen receptor 1 (ESR1; degree = 28) at the same time. This means that they are at the crossroads of steroid hormone receptor signaling, which is important to the main idea of this study. Third, molecular docking analyses revealed favorable binding affinities of ergosterol to AR and AKT1 (−11.4 kcal·mol^-1^), and of quercetagetin to ESR1, aligning with documented flavonoid–nuclear receptor interactions. Fourth, and most importantly, ergosterol is the only steroidal compound in the active pool. It has a tetracyclic carbon skeleton that is similar to endogenous androgens. Quercetagetin, on the other hand, is the only polyhydroxylated flavonoid that has been shown to have phytoestrogenic properties. It has a structural hormone-like pharmacophore that is not found in any other active candidate. The convergence of these criteria—in vivo dual-model efficacy, AR/ESR1 network centrality, molecular docking affinity toward steroidogenic targets, and hormone-mimicking structural pharmacophore—uniquely distinguished ergosterol and quercetagetin from the remaining active compounds and justified their selection for subsequent SMRT sequencing, mechanistic, ADMET, and nanocarrier translational analyses. The detailed results are as follows:

### 31 anti-cancer compounds from 7 kinds of natural substances *in vitro*

**FIGURE 1:**
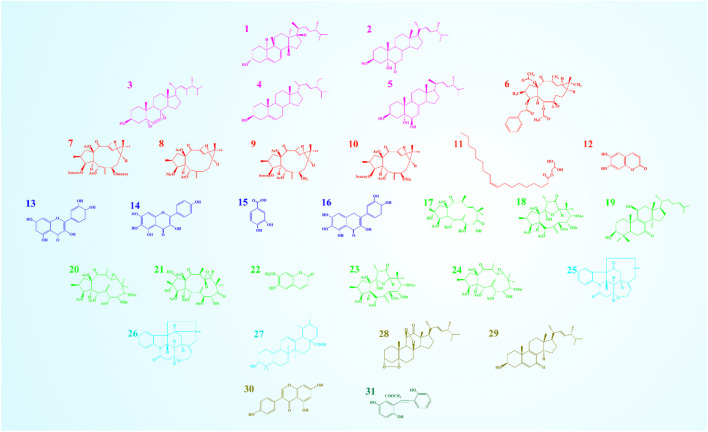
31 anticancer compounds from 7 kinds of natural substances *in vitro*. Same color represent the same origin. Ergosterol (1), Ergosta-7,22-dien-3*β*,5*α*- dihydroxy-6-one (2), 5*α*,8*α*-epidioxy-(22*E*,24*R*)-ergosta-6,22-dien-3*β*-ol (3), (24*S*)-5,22-stigmastadien -3*β*-ol (4) and Ergosta-7,22-diene-3,5,6-triol (5), Euphorbia factor L1 (6), Euphorbia factor L2 (7), Euphorbia factor L3 (8), Euphorbia factor L8 (9), Euphorbia factor L9 (10), Glyceryl monooleate (11) and esculetin (12), Quercetin (13), 6-hydroxykaempfe-rol (14), Protocatechuic acid (15) and Quercetagetin (16), Esulone A (17), Kansuinin A (18), (3*β*, 11*β*)-3, 11-dihydroxylanosta-8, 24-dien-7-one (19), Kansuinin E (20), Kansuinin B (21), Isoscopoletin (22), Kansuinin D (23) and Kansuinin G (24), Icajine (25), Strychnine-N-oxide (26) and Ursolic acid (27), 3*β*-hydroxy-(22*E*, 24*R*)-ergosta-5,8,22-trien-7-one (28), (3*α*, 5*α*), (8*β*, 11*β*)-diepidioxy-ergost-22*E*-en-12- one (29) and Genistein (30), Diphenylacetylene (31).

By using solvent chromatography, 31 compounds were obtained from 7 kinds of natural substance*s*. These compounds were ergosterol (1), ergosta-7,22-dien-3*β*, 5*α*-dihydroxy-6-one (2), 5*α*,8*α*-epidioxy-(22*E*,24*R*)-ergosta-6,22-dien-3*β*-ol (3), (24*S*)-5,22-stigmastadien-3*β*-ol (4), and ergosta-7,22-diene-3,5,6-triol (5) from the *C. militaris* HN. Euphorbia factor L1 (6), Euphorbia factor L2 (7), Euphorbia factor L3 (8), Euphorbia factor L8 (9), Euphorbia factor L9 (10), glyceryl monooleate (11), and esculetin (12) from *E. lathyris*. quercetin (13), 6-hydroxykaempferol (14), protocatechuic acid (15), and quercetagetin (16) from *T. erecta*; esulone A (17), kansuinin A (18), (3*β*, 11*β*)-3,11- dihydroxylanosta-8,24-dien-7-one (19), kansuinin E (20), kansuinin B (21), isoscopoletin (22), kansuinin D (23), and kansuinin G (24) from *E. kansui*. icajine (25), strychnine-N-oxide (26), and ursolic acid (27) from *S. nux-vomica*. 3*β*-hydroxy-(22*E*,24*R*)-ergosta-5,8,22-trien-7-one (28), (3*α*,5*α*), (8*β*,11*β*)-diepidioxy-ergost-22*E*-en-12-one (29), and genistein (30) from *A. luteo-virens*. And diphenylacetylene (31) from *S. stoloniferum* (Figure 1). Detailed data on structures can be downloaded from https://figshare.com/articles/dataset/Anti-cancer_components_from_Traditional_Chinese_Medicine_and_its_mechanism/13670731.

### Drug-like properties of 31 compounds

**FIGURE 2:**
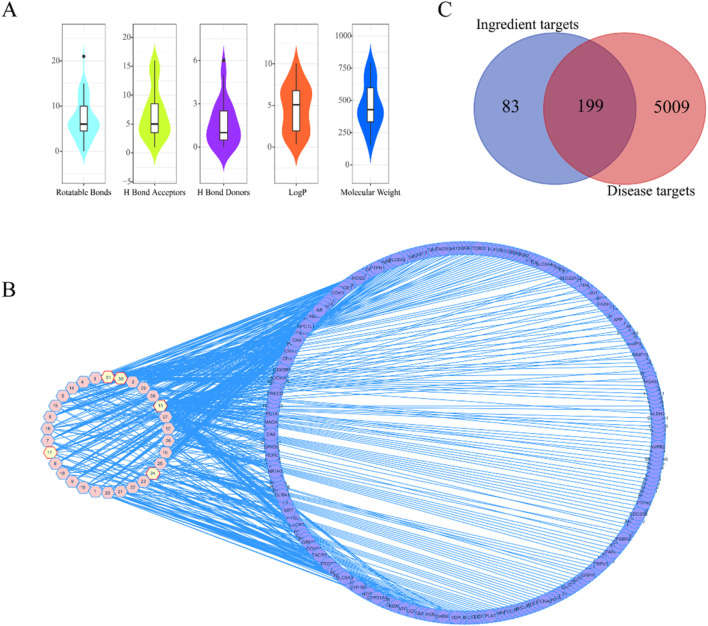
Druggability of 31 compounds and its cancer targets. **(A)** The Lipinski like-drug 5 principles. Distribution from 31 active compounds. the relative molecular mass (MW), the number of hydrogen bond acceptors (HBA), the number of hydrogen bond donors (HBD), the oil-water partition coefficient value (log P), topological polar surface area (TPSA), and the number of rotatable bonds (NROTB). **(B)** The network of 31 active compounds and targets. The nodes of the red hexagon represented compounds, the nodes of the blue circle represented targets, and the edges represented the relationship between compounds and targets. **(C)** The Venn diagram of compounds and targets. Compounds’ 199 mutual targets were obtained from anti-liver cancer and anti-lung cancer.

The results show that 31 compounds (1–31) inhibited HepG2 cells growth (Supplementary Figure S1) and 22 compounds (1–10, 12–16, and 24–31) inhibited A549 cells growth (Supplementary Figure S2) at 50 μg·mL^-1^. Among the 31 compounds, 8 compounds fully complied with Ro5 parameters (compliance rate was 25.8%). In detail, the MW of 11 compounds was greater than 500, log P of 16 compounds was greater than 5, NROTB of 7 compounds exceeded 10, HBAN of 6 compounds exceeded 10, and only compound 16 did not meet the HBDN parameter. Compounds 12, 13, 14, 15, 22, 25, 26, and 30 fully qualified the rule (Figure 2A; Supplementary Table S4).

### Compounds-targets network

Using the Swiss Target Prediction platform, 282 targets were obtained. After importing 31 compounds and 282 targets into Cytoscape, the compound–target network was constructed with 312 nodes and 550 edges, the freedom degree ranged from 1 to 89 (the average freedom degree was 3.526, and the median was 1). As shown in Figure 2B, in the compound–target network, the compounds had a freedom degree greater than average, different targets corresponded to the same compound, and the same target corresponded to different compounds. Compounds with the characteristics of multiple targets included 13 (degree = 89), 17 (degree = 21), 24 (degree = 19), and 30 (degree = 19).

### Key targets of liver and lung cancers

The number of disease targets related to anti-liver cancer and anti-lung cancer activity was 2,713 from GeneCard, 2,679 from GenCLiP 3, and 3,882 from DisGeneT. After removing the duplicates, 5,208 disease targets were obtained (Supplementary Table S1), these and 282 targets from the Swiss Target Prediction platform were input into the VennDiagram software to draw Venn diagrams and find the intersection. As shown in Figure 2C, 199 mutual targets with anti-liver cancer and anti-lung cancer activity were obtained.

### PPI network and 52 key targets

**FIGURE 3:**
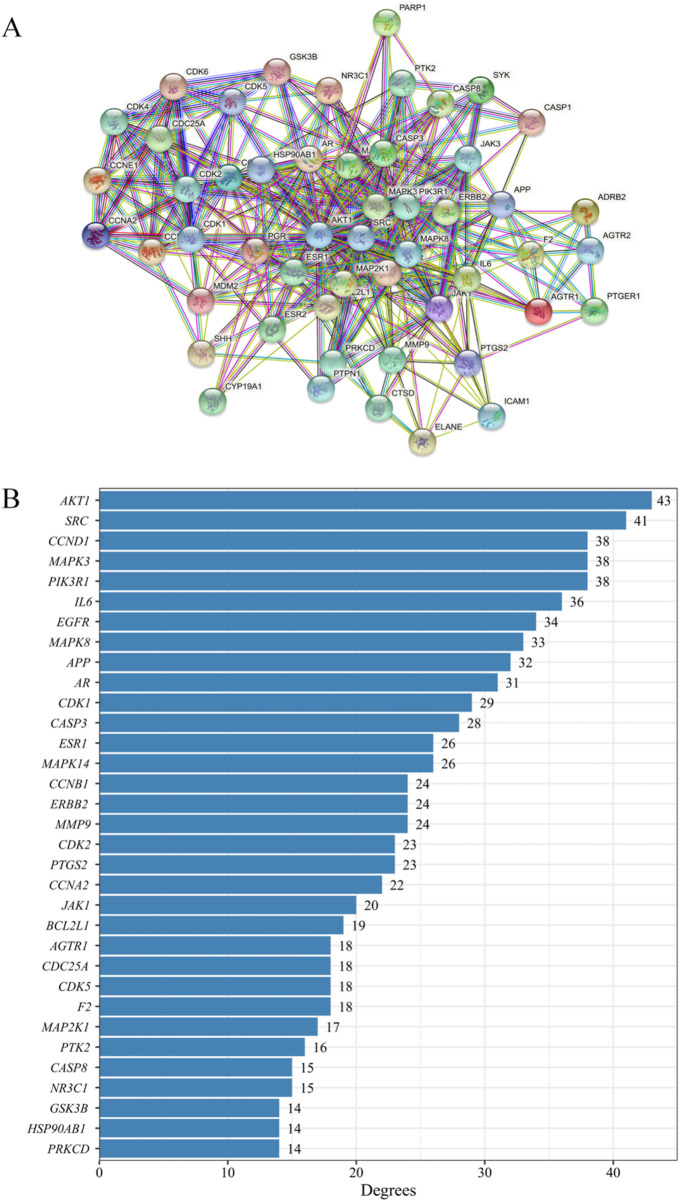
Screening key targets of anti-lung cancer and anti-liver cancer compounds. **(A)** The protein-protein interaction (PPI) network and key targets. There were 52 key targets from the PPI network. **(B)** Key targets. The key targets from the degree value top 33.

As shown in Figure 3A, 199 targets were entered into the String online database. After deleting the targets outside the network, the PPI network was obtained with 173 nodes, 878 edges, and an average degree of 10. Finally, 52 key targets were met (Figure 3B). DC (median = 7), BC (median = 78.4214), CC (median = 0.3517), and EC (median = 0.029), all four items were greater than 1 time of their median, and their first 33 were shown in Figure 3B (Supplementary Table S5). Among these, the top 14 targets with a degree greater than 25 were AKT1, SRC, CCND1, MAPK3, PIK3R1, IL6, EGFR, MAPK8, APP, AR, CDK1, CASP3, ESR1, and MAPK14, and these targets were considered as anti-cancer targets in the liver and lung.

### Screening out key pathways

Using the ClusterProfiler R package, the 52 key targets were analyzed for GO function and KEGG pathway annotation.

**FIGURE 4:**
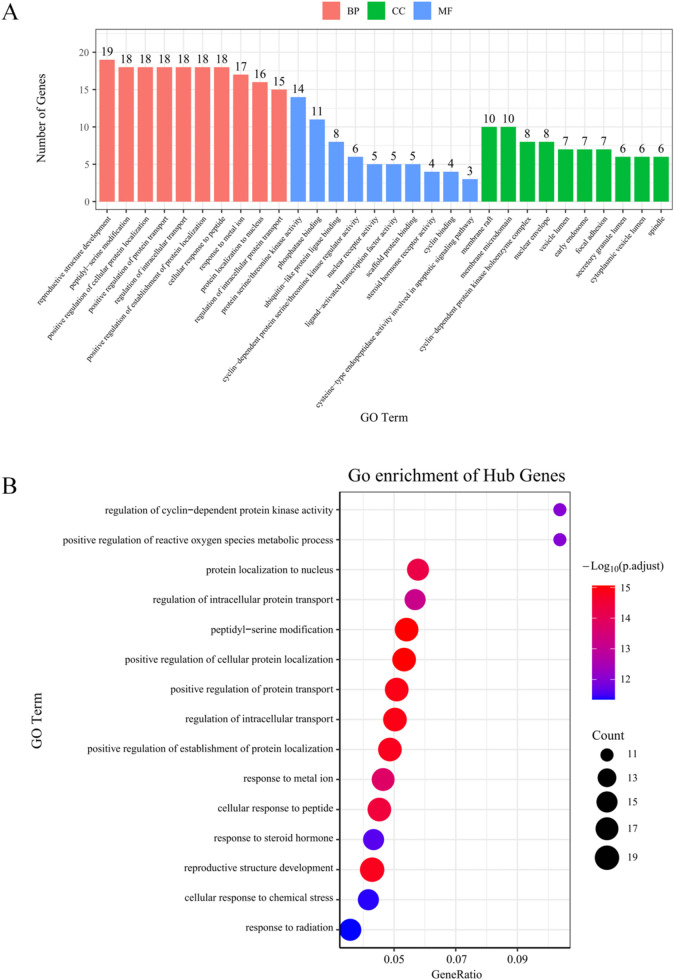
Fifty-two key targets were analyzed by GO function and KEGG pathway annotation. **(A)** GO functions from 52 key targets (the top 10). **(B)** The KEGG bubbles from 52 key targets by analyzing the top 15 (P values).

As a result, 522 GO functions were obtained. Among them, 443 GO functions were related to biological processes, 53 were related to molecular functions, and 26 were related to cellular components. The top 15 biological processes were divided into four categories related to (1) protein transport and protein cell localization regulation, such as positive regulation of cellular protein localization (18 targets), positive regulation of protein transport (18 targets), regulation of intracellular transport (18 targets), cellular response to peptide (18 targets), protein localization to nucleus (16 targets), positive regulation of establishment of protein localization (18 targets), and regulation of intracellular protein transport (15 targets). (2) cell structure reconstruction, such as peptidyl-serine modification (18 targets) and reproductive structure development (19 targets). (3) responding to hormones, chemical substances, and metals, such as response to steroid hormone (15 targets), cellular response to chemical stress (15 targets), positive regulation of reactive oxygen species metabolic process (11 targets), and response to metal ions (17 targets). and (4) cell cycle regulation, such as regulation of cyclin-dependent protein kinase activity (11 targets). GO molecular function enrichment analysis showed phosphatase binding (11 targets), protein serine/threonine kinase activity (14 targets), and cyclin-dependent protein serine/threonine kinase regulator activity (6 targets). The bar graph displayed the biological processes, cellular components, and molecular functions. The top 10 GO results of cellular components and molecular functions are shown in Figure 4A.

For KEGG annotation, 138 KEGG pathways were obtained. The main pathways were Endocrine resistance (16 targets), Human cytomegalovirus infection (19 targets), Kaposi sarcoma-associated herpesvirus infection (18 targets), Hepatitis B (16 targets), PI3K-Akt (20 targets), Pancreatic cancer (12 targets), Viral carcinogenesis (16 targets), and P53 signaling pathway (11 targets). A KEGG bubble chart of the top 15 (*P*-values) is shown in Figure 4B. Synthetically, the hepatitis B, hepatitis C, and PI3K-Akt pathways were closely related and could be used to treat liver cancer.

### Compound–target–pathway network

The compound–target–pathway network was obtained by 108 nodes and 781 edges. Among these, 28 nodes represented compounds, 52 nodes represented targets, and 25 nodes represented pathways. Of the 28 compounds, 23 interacted with more than two key targets per compound, and per target (52) corresponded to more than two pathways. Thus, these compounds had anti-cancer properties due to multiple interactions with targets and pathways.

**FIGURE 5:**
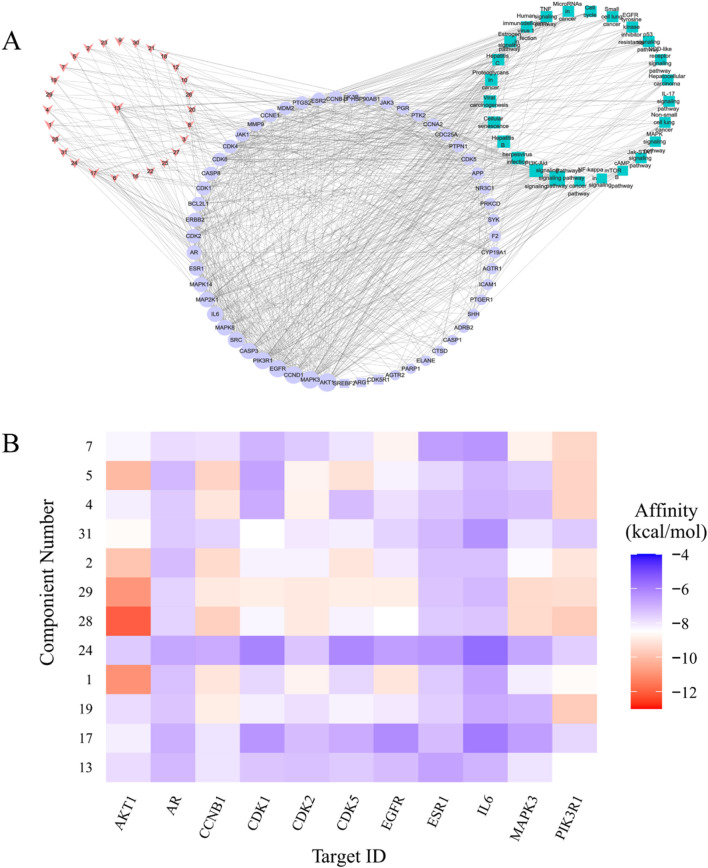
Screening of key medicinal targets. **(A)** The key compound-target-pathway network. The nodes of the red V shape represented active compounds, the nodes of the blue circle represented key targets, the nodes of the light green square represented pathways, and the edges of the gray line represented the interaction. **(B)** Molecular docking. The heat map of binding energy from active compounds and key targets.

According to the network topology analysis, the average degree of compounds was 4.36, and 12 compounds were greater than the average. The average degree of key targets was 21.67, and 24 targets were greater than the average. The average degree of pathways was 12.32, and 9 targets were greater than the average (Figure 5A). Compounds that might be the main active ingredients in lung and liver cancer treatment were 13 (degree = 18), 17 (degree = 10), 24 (degree = 7), 31 (degree = 7), 1 (degree = 6), 28 (degree = 6), 4 (degree = 6), 19 (degree = 5), 29 (degree = 5), 2 (degree = 5), 5 (degree = 5), and 7 (degree = 5). Targets that might be important for treating liver and lung cancer were AKT1 (degree = 48), MAPK3 (degree = 45), CCND1 (degree = 43), EGFR (degree = 42), PIK3R1 (degree = 41), CASP3 (degree = 37), SRC (degree = 36), MAPK8 (degree = 35), IL6 (degree = 34), MAP2K1 (degree = 33), MAPK14 (degree = 31), AR (degree = 28), ESR1 (degree = 28), and CDK2 (degree = 27). Pathways that might be meaningful for treating liver and lung cancer were pathways in cancer (degree = 30), PI3K-Akt signaling pathway (degree = 20), Herpesvirus infection (degree = 18), Cellular senescence (degree = 16), Hepatitis B (degree = 16), Viral carcinogenesis (degree = 16), Proteoglycans in cancer (degree = 15), estrogen signaling pathway (degree = 13), Hepatitis C (degree = 13), TNF signaling pathway (degree = 12), Human immunodeficiency virus 1 infection (degree = 12), and P53 signaling pathway (degree = 11).

### Eleven medicinal targets by molecular docking

The interaction between the compound ligand and protein receptor was simulated by molecular docking, and the best binding position and binding affinity were predicted. According to the degree of connection between the protein and the compound in the compound–target–pathway network, these compounds were 13, 17, 19, 1, 24, 28, 29, 2, 31, 4, 5, and 7 (related to potential targets), and they docked the activity pockets of AKT1, AR, CCNB1, CDK1, CDK2, CDK5, EGFR, ESR1, IL6, MAPK3, PIK3R1. The conformation with the lowest binding energy (the lower the binding energy, the more stable the conformation) was selected as the binding site (Figure 5B), and the crystal structure of the target protein see the (Supplementary Table S6). Case 1: The docking result suggests that compound-AKT1 affinity from high to low (binding energy/score from low to high) were No. 28 (−12.5 kcal·mol^-1^), 1 (−11.4 kcal·mol^-1^), and 29 (−11.3 kcal·mol^-1^), and their scores were close. Case 2: Compound 29 bound to cyclin-dependent kinases (CDK), CDK1 (−8.3 kcal·mol^-1^), CDK2 (−9.1 kcal·mol^-1^), and CDK5 (−8.2 kcal·mol^-1^). Case 3: The key target EGFR could form stable conformations with some compounds, No. 1 (−9.2 kcal·mol^-1^), 29 (−9 kcal·mol^-1^), and 7 (−8.8 kcal·mol^-1^). Case 4: Compound 13 could bind to multiple key targets, such as PIK3R1 (−8.5 kcal·mol^-1^), MAPK3 (−7.9 kcal·mol^-1^), CCNB1 (−7.9 kcal·mol^-1^), and AKT1 (−7.7 kcal·mol^-1^).

### Seven medicinal compounds by Lewis and H22 tumor *in vivo*

**FIGURE 6:**
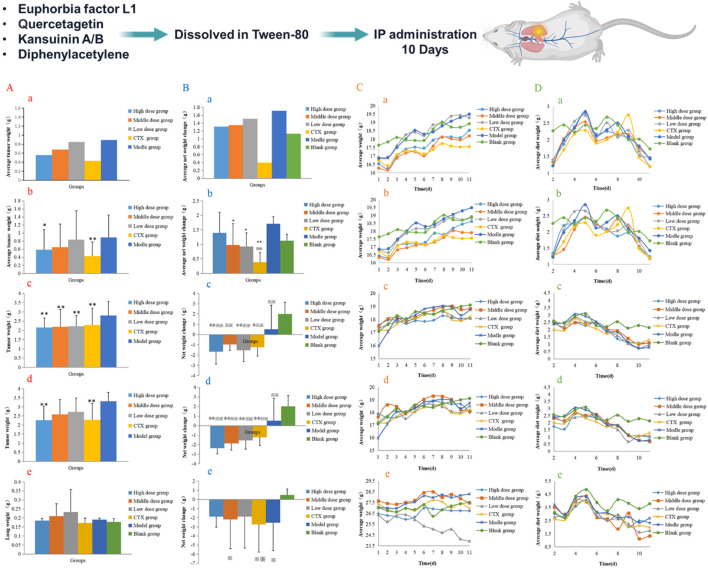
Five compounds had anti-Lewis lung efficacy *in vivo*. **(A)** The average lung tumor weight. a: Euphorbia factor L1 (6), b: Quercetagetin (16), c: Kansuinin A (18), d: Kansuinin B (21), e: Diphenylacetylene (31). **(B)** The average changes of net weight a: Euphorbia factor L1 (6), b: Quercetagetin (16), c: Kansuinin A (18), d: Kansuinin B (21), e: Diphenylacetylene (31. **(C)** The average weight changes a: Euphorbia factor L1 (6), b: Quercetagetin (16), c: Kansuinin A (18), d: Kansuinin B (21), e: Diphenylacetylene (31). **(D)** The average diet changes a: Euphorbia factor L1 (6), b: Quercetagetin (16), c: Kansuinin A (18), d: Kansuinin B (21), e: Diphenylacetylene (31).

The tumor-bearing Lewis mice, as the mice model, were divided into 3 groups, namely, the compound group, the model group (blank group), and the positive group (cyclophosphamide). Compounds were suspended in Tween-80 to promote dissolution and then prepared by adding physiological saline. The positive drug control was prepared according to the same method, and 3 groups were continuously administered for 10 days. When administered at 54 mg·kg^-1^ for 10 days (dose selected based on a preliminary pilot tolerability study described in Materials and Methods), euphorbia factor L1 showed efficacy in Lewis tumor-bearing mice and alleviated splenomegaly. When the dose was 180 mg·kg^-1^ for 10 days, quercetagetin had a certain curative effect on Lewis mice. When the administration dose was 11.11 mg·kg^-1^ for 10 days, kansuinin A had a certain curative effect on Lewis mice, and it can alleviate the splenomegaly. When the administration dose was 99.99 mg·kg^-1^ for 10 days, kansuinin B had a certain curative effect on Lewis mice, and it can alleviate the splenomegaly phenomenon. When the administration dose was 19.2 mg·kg^-1^ for 10 days, diphenylacetylene had a good effect on Lewis mice, mainly by destroying the body’s cellular immune function, thereby inhibiting the growth of tumor cells. The above is shown in Figure 6.

**FIGURE 7:**
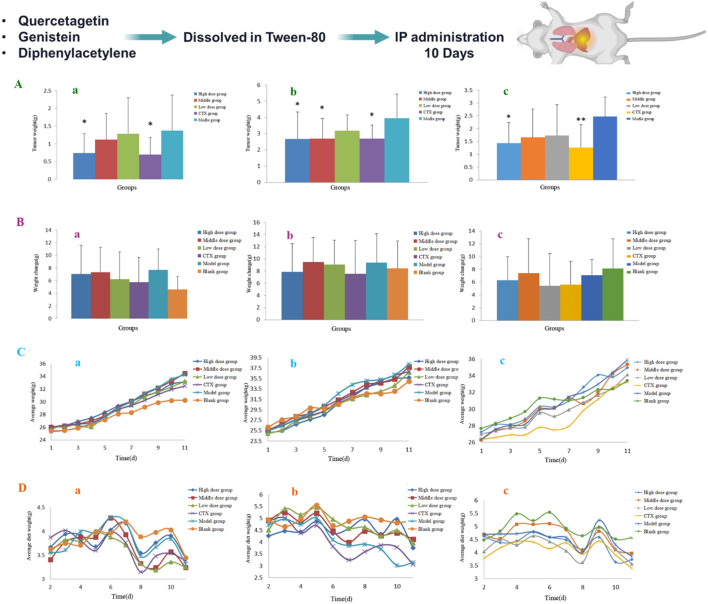
Three compounds had the anti-H22 liver efficacy *in vivo*. **(A)** The average liver tumor weight. a: Quercetagetin (16), b: Genistein (30), c: Diphenylacetylene (31). **(B)** the average changes of net weight a: Quercetagetin (16), b: Genistein (30), c: Diphenylacetylene (31). **(C)** The average weight changes a: Quercetagetin (16), b: Genistein (30), c: Diphenylacetylene (31). **(D)** The average diet changes a: Quercetagetin (16), b: Genistein (30), c: Diphenylacetylene (31).

The tumor-bearing H22 mice, as the mice model, were divided into 3 groups, namely the compound group, the model group (blank group), and the positive group (cyclophosphamide). compounds were suspended in Tween-80 to promote dissolution and then prepared by adding physiological saline. The positive drug control was prepared according to the same method, and 3 groups were continuously administered for 10 days. When the dosage was 240 mg·kg^-1^ for 10 days, these mice were dissected and observed 24 h after the last administration. When the dose was 180 mg·kg^-1^ for 10 days, quercetagetin had a significant curative effect on H22 mice. When the dosage was 73.71 mg·kg^-1^ for 10 days, genistein had a certain curative effect on H22 mice, and it can alleviate the shrinkage of the thymus and splenomegaly. When the dosage was 57.6 mg·kg^-1^ for 10 days, diphenylacetylene had a good effect on H22 mice, mainly by destroying the body’s cellular immune function, thereby inhibiting tumor cell growth. The above is shown in Figure 7.

For the Lewis lung tumor, 5 compounds: euphorbia factor L1, quercetagetin, kansuinin A, kansuinin B, and diphenylacetylene had anti-cancer efficacy. for the H22 liver tumor, 3 compounds: quercetagetin, genistein, and diphenylacetylene had anti-cancer efficacy. Based on previous analyzing the efficacy of active compounds, diphenylacetylene had the strongest effect on lung cancer and ergosterol had the strongest effect on liver cancer (Supplementary Figure S3).

Seven of the active compounds meet the requirements for animal experiments, namely Euphorbia factor L1, Quercetagetin, Kansuinin A, Kansuinin B, Diphenylacetylene, Genistein, Ergosterol.

### Hormone-associated pathway analysis suggests involvement of AR/ESR1-3*β*HSD/17*β*HSD in hormone-like ergosterol/quercetagetin anti-cancer activity

By SMRT sequencing, 45,146 complete cDNA sequences from A549 cells were obtained. The transcriptome size was 59559 kb, and the transcript length ranged from 200 to 7,497 bp with an N50 of 1,328 bp. The average GC content was 48.36%, and the contig N50 was 1,490 bp. Comparing the findings of the transcriptome assembly with 5,000 enzymes (from NCBI), 2,660 enzymes (including 4 mitochondrial and 2,656 non-mitochondrial) were detected, of which 192 were mutated (Supplementary Table S7). And 46,512 complete cDNA sequences were obtained. The transcriptome size was 69,890,247 kb, and the transcript length ranged from 203 to 8,656 bp with N50 of 1,502 bp. The average GC content was 48.37%, and the contig N50 was 1,651 bp. Comparing the findings of the transcriptome assembly with 5,000 enzymes (disease), 2,660 enzymes (including 4 mitochondrial and 2,656 non-mitochondrial) were detected, of which 340 were mutated (Supplementary Table S10). The total sequence alignment data can be downloaded from https://figshare.com/articles/dataset/Anti-cancer_components_from_Traditional_Chinese_Medicine_and_its_mechanism/13670731.

**FIGURE 8:**
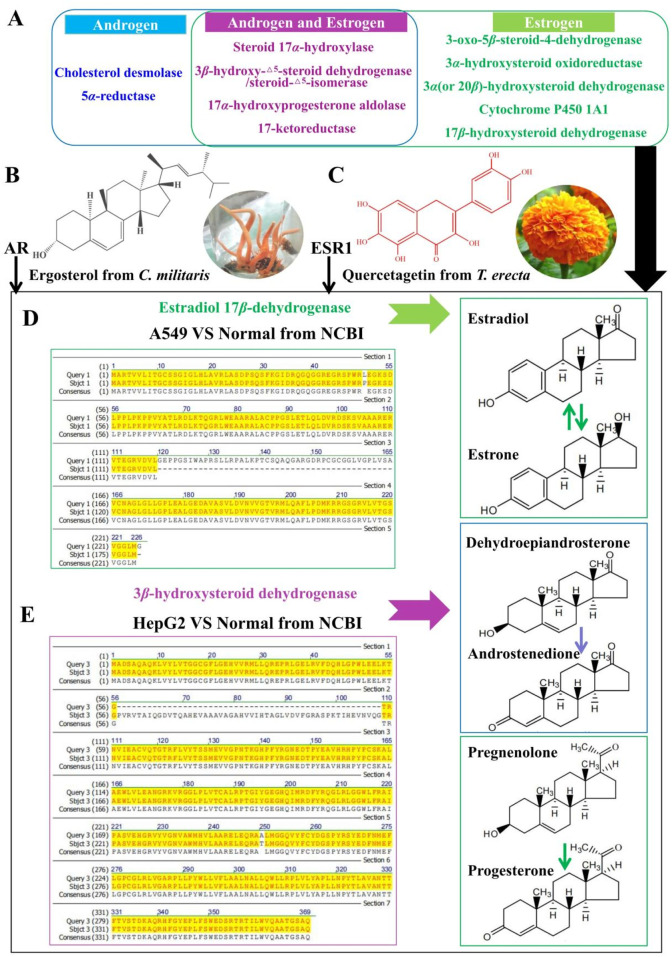
Hormone metabolism-associated mechanism of ergosterol and quercetagetin. **(A)** This study refers to the main catalytic enzymes of human hormone metabolism pathway. Among them, green is the catalytic enzyme of estrogen metabolism, blue is the catalytic enzyme of androgen metabolism, and purple is the catalytic enzyme needed by both. **(B)** Ergosterol, a drug substance anti lung cancer and anti-liver cancer, was obtained through *in vitro* and *in vivo* experiments. It is a natural androgen-like compound from *C. militaris*, and its medicinal target is mainly androgen receptor. **(C)** Quercetagetin, a drug substance anti lung cancer and anti-liver cancer, was obtained through *in vitro* and *in vivo* experiments. It is a natural estrogen-like compound from *T. erecta*, and its medicinal target is mainly Estrogen receptor. **(D)** Comparison with the amino acid sequence of estradiol 17*β*-dehydrogenase (17*β*HSD) from NCBI (Ref. XP_011523034.1), and this enzyme mainly catalyzes the reversible transformation of estradiol and estrone. **(E)** Comparison with the amino acid sequence of 3*β*-hydroxysteroid dehydrogenase from NCBI (Ref. NP_079469.2), and this enzyme not only catalyzes the conversion of dehydroepiandrosterone to androstenedione, but also catalyzes the conversion of pregnenolone to progesterone.

This study focuses on the key enzyme of hormone metabolism in Figure 8A. The catalytic pathways of these enzymes in human body are shown in support information (Supplementary Figures S4, S5). It was found that 2 enzymes were mutated by screening the mutant hormone metabolism enzymes.

Estrogen metabolizing enzyme gene mutation 1/192 in transcriptome of A549 cells (Supplementary Table S7). Specifically, the SMRT transcriptome sequencing results confirmed that 17*β*HSD was mutated in A549 cells (Supplementary Table S8), with 79% identity and gaps of 49 (Ref. XP_011523034.1). This enzyme mainly catalyzes the mutual transformation between estradiol and estrone (Figure 8D), and its amino acid sequence alignment of the mutant metabolic enzyme is shown in the support information (Supplementary Table S9).

On another enzyme, hormone metabolizing enzyme gene mutation 1/340 in transcript me of HepG2 cells (Supplementary Table S10 **)**. Specifically, the SMRT transcriptome sequencing results confirmed that 3*β*HSD was mutated in HepG2 cells (Supplementary Table S11), with 86% identity and gaps of 52 (ref. NP_079469.2). The amino acid sequence alignment of the mutant metabolic enzyme is shown in the support information (Figure 8C; Supplementary Table S12). This enzyme not only catalyzes the conversion of dehydroepiandrosterone to androstenedione in androgen metabolism pathway, but also catalyzes the conversion of pregnenolone to progesterone in estrogen metabolism pathway (Figure 8E).

The purpose of SMRT sequencing in this study was to identify whether key steroid-metabolism enzymes within the androgen/estrogen conversion axis show sequence divergence in the selected models (A549 and HepG2), thereby supporting the biological relevance of steroid-metabolism–associated pathways in these cells. Among the mutated enzymes detected, 17βHSD (A549) and 3βHSD (HepG2) were prioritized because they directly catalyze the estradiol/estrone interconversion and the DHEA/androstenedione conversion, respectively (Figures 8D,E).

To evaluate whether these sequence variants are likely to affect ligand interaction, we compared the mutant sequences against the corresponding reference proteins and mapped the non-identical regions onto structural models used for our *in silico* analysis. This mapping indicated that sequence differences are predominantly located in peripheral/non-catalytic regions, while residues lining the predicted steroid-binding/catalytic pocket remain conserved. Consistent with this, the putative mutation sites are spatially distant (typically >15 Å) from the ligand-binding center in the model, suggesting that the observed sequence divergence is unlikely to abolish ligand binding of sterol-/flavonoid-like compounds. However, because we did not perform direct enzyme activity assays on the variant proteins, the functional impact of these sequence changes should be interpreted as supportive/associative rather than definitive, and will be validated in future work.

In this study, there are 2 kinds of hormone-like compounds in 5 medicinal compounds obtained *in vitro* and *in vivo*, because ergosterol belongs to androgen-like (Figure 8B), while quercetagetin belongs to estrogen-like (Figure 8C). Furthermore, AR and ESR1 were found from 11 medicinal targets excavated from network pharmacology. Taken together, these findings are consistent with the hypothesis that these hormone-like drug compounds, may participate in androgen metabolism targeting AR/ESR1 by regulating 3*β*HSD/17*β*HSD, are related to the conversion of dehydroepiandrosterone to androstenedione and the reversible reaction between estradiol and estrone.

### Pharmacokinetic evaluation of ergosterol and quercetagetin and their nanocarrier design

**FIGURE 9:**
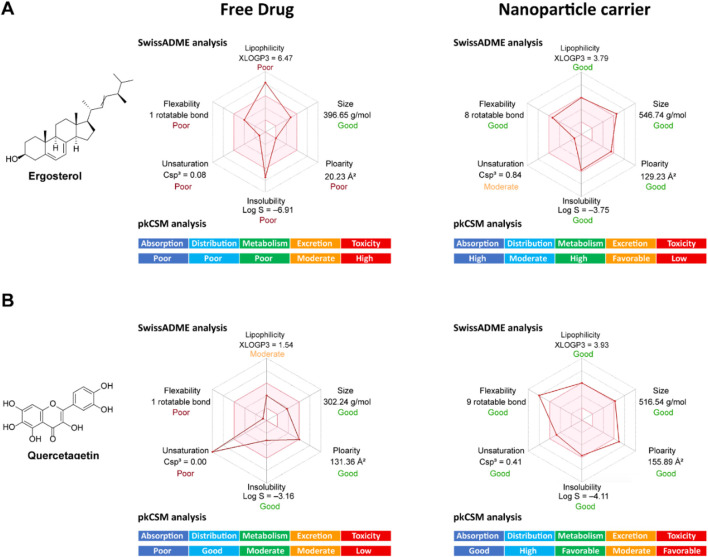
Pharmacokinetic optimization of ergosterol and quercetagetin via nanoparticle carriers. **(A)** Chemical structure and *in silico* pharmacokinetic predictions (SwissADME and pkCSM) of free ergosterol versus ergosterol conjugated to a mimic of cyclodextrin nanostructures. **(B)** Chemical structure and pharmacokinetic predictions of free quercetagetin versus quercetagetin conjugated a mimic of polymeric micelle nanostructures.

Despite their established anticancer potential, both ergosterol and quercetagetin face considerable pharmacokinetic barriers that limit their application in hormone-associated cancer therapy. These limitations necessitate the incorporation of nanodelivery strategies to fully realize their therapeutic potential in the native environment (Eladl, 2025a; Eladl, 2025b). Ergosterol displays potent bioactivity but suffers from extremely poor aqueous solubility (Log S = −6.91) and excessive lipophilicity (XLOGP3 = 6.47), both of which hinder oral bioavailability and membrane transport. Although it complies with drug-likeness filters, its low bioavailability score (0.55) and violations of the Veber, Ghose, Egan, and Muegge rules indicate limited suitability for drug development. Its low polarity (TPSA = 20.23 Å^2^) and low saturation (Fraction Csp^3^ = 0.08) reinforce these concerns. From a pkCSM perspective, ergosterol shows high intestinal absorption (94.4%) and favorable Caco-2 permeability (1.212 log Papp), but its complete plasma protein binding (Fraction unbound = 0.0) means there is no free drug available systemically. As a CYP3A4 substrate, it risks rapid metabolism and potential drug–drug interactions. While its total clearance (log CL = 0.704 mL/min/kg) is moderate, cardiotoxicity is suggested by predicted hERG II inhibition. These findings highlight the need for a nanocarrier system to enhance solubility, reduce lipophilicity, improve distribution, and mitigate toxicity (Figure 9A).

Similarly, quercetagetin holds strong therapeutic potential but presents distinct pharmacokinetic challenges. It has moderate lipophilicity (XLOGP3 = 1.54) and reasonable solubility (Log S = −3.16), with favorable values for molecular weight (302.24 g/mol) and rotatable bonds (1). However, its high polarity (TPSA = 131.36 Å^2^) and zero Csp^3^ fraction (0.00) limit its membrane permeability. According to pkCSM, it exhibits poor Caco-2 permeability (−0.229 log Papp), moderate intestinal absorption (77.2%), and is a P-glycoprotein (P-gp) substrate, raising concerns of efflux-mediated drug resistance. While the fraction unbound (0.206) and tissue distribution (VDss = 1.559 log L/kg) are favorable, its slow clearance (log CL = 0.407 mL/min/kg) and moderate enzyme interaction profile suggest it would benefit from enhanced delivery mechanisms. Toxicity predictions are favorable, with no signs of mutagenicity, hepatotoxicity, or cardiotoxicity. Taken together, although both compounds possess targeting activity and fit within ideal phytochemical frameworks, their suboptimal ADMET profiles limit their standalone development. Ergosterol is constrained by solubility, lipophilicity, plasma binding, and cardiotoxicity, while quercetagetin is hampered by poor permeability, P-gp efflux, and limited absorption (Figure 9B).

To overcome these limitations, we explored nanoparticle-based delivery systems tailored to each molecule. For ergosterol, we designed a glucose-based nanocarrier mimicking cyclodextrin architecture, a widely used approach to increase solubility and bioavailability. SwissADME and pkCSM predictions for the nanoconjugate showed a marked improvement in key parameters. Log P decreased from 6.47 to 3.92, entering the optimal range. Solubility improved over 1000-fold, as ESOL-predicted log S increased from −6.91 to −3.75. The unbound plasma fraction increased from 0.0 to 0.205, enhancing systemic exposure. Importantly, the nanocarrier conjugate maintained a non-toxic profile, showing no AMES mutagenicity, hepatotoxicity, or hERG inhibition. These enhancements significantly restore the pharmacokinetic viability of ergosterol for therapeutic use (Figure 9A).

For quercetagetin, we developed a branched alkyl ester nanocarrier, structurally resembling amphiphilic micelles. This conjugate displayed key improvements: Log P rose from 1.54 to 3.93, indicating enhanced membrane interaction. It was no longer predicted to be a P-gp substrate, suggesting reduced efflux and improved intracellular retention, and skin permeability increased (Log Kp from −8.98 to −5.90 cm/s). The fraction unbound rose from 0.206 to 0.389, enhancing pharmacologically active distribution. The nanoconjugate retained a clean toxicity profile, with no predicted mutagenicity, hepatotoxicity, or cardiotoxicity. In summary, these nanocarrier strategies effectively address the core pharmacokinetic deficits of ergosterol and quercetagetin, enabling their clinical potential as dual-targeting agents in hormone-associated cancers. By improving solubility, permeability, systemic availability, and toxicity profiles, these tailored delivery systems support the further development of both phytochemicals into viable anticancer therapeutics (Figure 9B).

## Discussion

This study verified that *C. militaris* HN fruiting body, *E. lathyris*, *T. erecta*, *E. kansui*, *S. nux-vomica*, *A. luteo-virens*, and *S. stoloniferum* showed anti-cancer activity. The *C. militaris* HN fruiting body is a new food resource. *A. luteo-virens* and *T. erecta* are edible. the medicinal drugs from *S. stoloniferum*, *E. kansui*, *E. lathyris*, and *S. nux-vomica* are contraindicated during pregnancy, and *E. kansui* and *S. nux-vomica* are toxic. Therefore, considering human safety, *C. militaris* HN fruiting body, *T. erecta*, and *A. luteo-virens* could be used to treat liver and lung cancer.

In this study, 31 active compounds were obtained from 7 kinds of natural materials, enriching the library of anti-cancer natural compounds. Importantly, because of hormone-like actions, ergosterol and quercetagetin were selected as potential compounds for further design of nanodrugs, that may be more suitable for clinical treatment for hormone-associated cancer patients.

Further experimental results showed that within a certain concentration range *in vivo*, euphorbia factor L1, quercetagetin, kansuinin A, kansuinin B, and diphenylacetylene had anti-lung cancer effects. Meanwhile, quercetagetin, genistein, and diphenylacetylene had anti-liver cancer effects. Based on the dose-effect relationship, diphenylacetylene had the strongest effect on lung cancer, and ergosterol had the strongest effect on liver cancer (Supplementary Figure S3).

Hormone metabolism is closely related to the occurrence and development of liver cancer (Rasmussen et al., 2013; Muñoz-Fonseca et al., 2021; Mei et al., 2022). And the ergosterol from *C. militaris* HN is a potential compound for treating liver cancer in this study. It is reported that the liver plays an important role in the metabolic process, and steroid metabolism disorder is identified as the potential driving factor of liver pathogenesis, while sterol metabolite accumulation can inhibit the growth of liver cancer cells (Lu et al., 2013). The latest research also found that steroid metabolism pathway reprogramming is directly related to the growth of liver cancer cells (Jiang et al., 2019). Scientifically targeting the enzymes related to hormone metabolism may be a breakthrough in cancer therapy. In this study, the natural ergosterol from *C. militaris* inhibited the growth of liver cancer *in vivo* and *in vitro*. Ergosterol has a sex hormone-like effect (Muñoz-Fonseca et al., 2021), and 3*β*HSD in liver is the key enzyme for steroid hormone production and the conversion between hormone activity and inactivity (Mei et al., 2022). Collectively, our efficacy results, network analysis, and hormone readouts are consistent with a model in which ergosterol influences androgen-related metabolism in HepG2 cells, potentially through AR-associated pathways and modulation of 3*β*HSD, resulting in reduced DHEA→androstenedione conversion. However, direct measurements of 3*β*HSD activity/expression and AR signaling were not performed, and therefore this mechanism should be interpreted as hypothesis-generating and requires targeted validation.

Mechanistic inferences in this study are primarily supported by integrative *in silico* analyses and hormone/metabolite observations; definitive causality will require enzyme activity assays, receptor perturbation (e.g., knockdown/inhibitor studies), and direct binding/biophysical validation.

Importantly, a follow-up study incorporating direct enzymatic measurements (e.g., 3*β*HSD/17*β*HSD activity and expression) together with receptor-defined models (e.g., AR- or ER-signaling–relevant cancer systems) will be essential to substantiate the proposed mechanism and strengthen causal interpretation.

From a translational perspective, biomimetic nanocarriers (including cell membrane–coated and other bioinspired systems) have been widely explored to improve circulation time, biocompatibility, and functional targeting of therapeutics, with potential to enhance efficacy and reduce systemic toxicity (Fang et al., 2023). Therefore, a logical next step is to formulate ergosterol in a biomimetic nano-delivery system and evaluate pharmacokinetics, biodistribution, efficacy, and safety in preclinical liver cancer models.

## Data Availability

The original contributions presented in the study are publicly available. This data can be found here: https://doi.org/10.6084/m9.figshare.13670731.
