# Supplementary material for: Dual-targeting phytochemicals Ergosterol and Quercetagetin implicate steroid metabolism–associated pathways in lung and liver cancer models
Source: Front Pharmacol. 2026 May 1;17:1773130. doi: 10.3389/fphar.2026.1773130 (PMC13176194; doi:10.3389/fphar.2026.1773130)
Supplement: Supplementary file 1 [file Supplementaryfile1.docx]

**Supplementary Materials**

**Dual-Targeting Phytochemicals Ergosterol and Quercetagetin Implicate Steroid Metabolism–Associated Pathways in Lung and Liver Cancer Models**

Yujiao Chen^1,2,3,4,5&*^, Yuqian Wu^4&^, Madineh Moradialvand^6^, Omar Eladl^6,7^, Simiao Du^8^, Shanshan Yang^8^, Xiaomin Liu^8^, Shujun Zhang^9^, Jianqing Chen^10^, Jingwen Zeng^11^, Xiaowei Su^11^, Hong Ma^8^, Hong Lu^8^, Jianhua Feng^12^, Jun Cao^4^, Li Zhong^3^, Guixue Wang^3,5*^, Jun Yang^1,2*^**,** Hasan Karimi Maleh^6,13^, Pooyan Makvandi^6^**^,^**^14,15*^

1 Zhejiang University School of Medicine, Hangzhou, 310058, China

2 Zhejiang University, Department of Physiology, Department of Cardiology of the Second Affiliated Hospital and School of Basic Medical Sciences & State Key Laboratory of Transvascular Implantation Devices & Jade Biotechnology, Hangzhou, 310009, China

3 Key Laboratory for Biorheological Science and Technology of Ministry of Education, National Local Joint Engineering Laboratory for Vascular Implants, Bioengineering College of Chongqing University, Chongqing 400030, China

4 Guizhou Gui’an Academy of Precision Medicine, Gui’an, Guizhou, 561113, China

5 Institute of Panvascular Biology, JinFeng Laboratory, Chongqing, 401329, China

6 The Quzhou Affiliated Hospital of Wenzhou Medical University, Quzhou People’s Hospital, Quzhou, 324000, Zhejiang, China

7 Faculty of Pharmacy, Egypt-Japan University of Science and Technology (E-JUST), Alexandria, Egypt

8Zheng YuanTang (Tianjin) Biotechnology, Tianjin, 300457, China

9 School of Basic Medical Sciences, Southwest Medical University, Luzhou, Sichuan, 646000, China

10 College of Life Sciences and Medicine, Zhejiang Provincial Key Laboratory of Silkworm Bioreactor and Biomedicine, Zhejiang Sci-Tech University, Hangzhou, 310018, China

11 Guizhou Aerospace Intelligent Agriculture, Guiyang, Guizhou, 057250, China

12 Tianjin Lakeside Powergene Science Development, Tianjin, 300309, China

13 School of Chemistry, Damghan University, Damghan, 36716-45667, Iran

14 University College, Korea University, Seoul, 02841, Republic of Korea

15 Centre for Research Impact and Outcome, Chitkara University, Rajpura, 140401, Punjab, India

& Equally contributed to this work

***Corresponding author**

[chenyujiao623198@126.com](mailto:chenyujiao623198@126.com) (Y. Chen); [wanggx@cqu.edu.cn](mailto:wanggx@cqu.edu.cn) (G. Wang); yang_jun@zju.edu.cn (J-Yang); [pooyanmakvandi@gmail.com](mailto:pooyanmakvandi@gmail.com), [Pooyan.makvandi@wmu.edu.cn](mailto:Pooyan.makvandi@wmu.edu.cn) (P. Makvandi)

**1 Established the Lewis lung carcinoma mice model**

The mice in good condition after 8-13 days of tumor inoculation were put to death, and the body surface of the mice was swabbed with 75% alcohol. LLC cells were separated from mice and removed to a sterile plate containing normal saline. Put the LLC cells into the tissue homogenizer, added the physiological salt at a volume ratio of 1:3, and finally, poured the homogenate into a 50 mL centrifuge tube. Injected 0.2 mL LLC cells suspension into the right subaxillary (The process of preparing LLC cells suspension was completed under aseptic conditions; Tumor inoculation needed to be completed within 60 minutes ).

**Table S1. Statistics the related targets of lung and liver cancers from 3 databases**

| **Database** | **Lung** | **Liver** | **Number**  **（After removing the duplication）** |
| --- | --- | --- | --- |
| GeneCard | 2250  （Relevance score ≥ 8.24） | 1854  (Relevance score ≥ 4.07) | 2713 |
| GenCLiP 3 | 1564 | 2054 | 2679 |
| DisGeNET | 2006 | 3303 | 3882 |
| All | 3551 (2010,812,729) | 4063 (1941,1095,1027) | 5208 (2496,1358,1354) |

**Table S2. Experimental method of anti-lung cancer efficacy of active ingredients**

| **Compound** | **Drug preparation method** | **Anti-Lewis lung carcinoma drug effect grouping and administration method** |
| --- | --- | --- |
| Ergosterol from *Cordyceps militaris* | Ergosterol gavage: Weighed the ergosterol sample into the mortar, added 2% Tween-80, added normal saline after thorough grinding, and mixed it thoroughly.  Cyclophosphamide gavage: Weighed the cyclophosphamide sample into the mortar, added normal saline, and mixed it thoroughly. | The 60 SD rats were divided into a low-dose group (ergosterol 8.19mg/kg), a middle-dose group (ergosterol 24.57mg/kg), a high-dose group (ergosterol 73.71mg/kg), a cyclophosphamide group, ⑤a model group,⑥ a blank group, 10 mice in each group, half male and half female. Except for the blank and the model group, the others were administered per day after the injection of tumor suspension, each injection was 0.2 mL. The blank group and the model group were given 2% tween-80 aqueous solution by gavage for the same time, and all were continuously administered for 10 days. |
| Quercetagetin from *Tagetes erecta* L. | Quercetagetin gavage: Weighed the quercetagetin sample into the mortar, added 2% Tween-80, added normal saline after thorough grinding, and mixed it thoroughly.  Cyclophosphamide gavage: After removing the film coat of the cyclophosphamide sheet, weighed the cyclophosphamide sample into the mortar, added normal saline, and mixed it thoroughly. | The C57BL/6 mice were divided into a low-dose group (quercetagetin 20 mg/kg), a middle-dose group (quercetagetin 60mg/kg), a high-dose group (quercetagetin 180mg/kg), a cyclophosphamide group, a model group, a blank group, 10 mice in each group, half male and half female. Except for the blank and the model group, the others were administered per day after the injection of tumor suspension, each injection was 0.2 mL. The blank group and the model group were given 2% tween-80 aqueous solution by gavage for the same time, and all were continuously administered for 10 days. |
| Kansuinine A from *Euphorbia kansui* L. | Kansuinine A gavage: Weighed the kansuinine A sample into the mortar, added 2% Tween-80, added normal saline after thorough grinding, and mixed it thoroughly.  Cyclophosphamide gavage: Weighed the cyclophosphamide sample into the mortar, added normal saline, and mixed it thoroughly. | The 60 C57BL/6 mice were divided into a low-dose group (Kansuinine A 11.11 mg/kg), a middle-dose group (Kansuinine A 33.33 mg/kg), a high-dose group (Kansuinine A 99.99mg/kg), a cyclophosphamide group, a model group, a blank group, 10 mice in each group, half male and half female. Except for the blank and the model group, the others were administered once a day after the injection of tumor suspension, each injection was 0.2 mL. The blank group and the model group were given 2% tween-80 aqueous solution by gavage for the same time, and all were continuously administered for 10 days. |
| Kansuinine B from *Euphorbia kansui* L. | Kansuinine B gavage: Weighed the kansuinine B sample into the mortar, added 2% Tween-80, added normal saline after thorough grinding, and mixed it thoroughly.  Cyclophosphamide gavage: Weighed the cyclophosphamide sample into the mortar, added normal saline, and mixed it thoroughly. | The 60 C57BL/6 mice were divided into a low-dose group (Kansuinine B 11.11 mg/kg), a middle-dose group (Kansuinine B 33.33 mg/kg), a high-dose group (Kansuinine B 99.99mg/kg), a cyclophosphamide group, a model group, a blank group, 10 mice in each group, half male and half female. Except for the blank and the model group, the others were administered per day after the injection of tumor suspension, each injection was 0.2 mL. The blank group and the model group were given 2% tween-80 aqueous solution by gavage for the same time, and all were continuously administered for 10 days. |
| Diphenylacetylene from *Sparganium stolonifeum* Buch.-Ham. | Diphenylacetylene gavage: Weighed the diphenylacetylene sample into the mortar, added 2% Tween-80, added normal saline after thorough grinding, and mixed it thoroughly.  Cyclophosphamide gavage: After removing the film coat of the cyclophosphamide sheet, weighed the cyclophosphamide sample into the mortar, added normal saline, and mixed it thoroughly. | The C57BL/6 mice were divided into a low-dose group (diphenylacetylene 6.4 mg/kg), a middle-dose group (diphenylacetylene 19.2 mg/kg), a high-dose group (diphenylacetylene 57.6 mg/kg), a cyclophosphamide group, a model group, a blank group, 10 mice in each group, half male and half female. Except for the blank and the model group, the others were administered per day after the injection of tumor suspension, each injection was 0.2 mL. The blank group and the model group were given 2% tween-80 aqueous solution by gavage for the same time, and all were continuously administered for 10 days. |
| Euphorbia factor L1 from *Euphorbia lathyris* L. | Euphorbia factor L1 gavage: Weighed the euphorbia factor L1 sample into the mortar, added 2% Tween-80, added normal saline after thorough grinding, and mixed it thoroughly.  Cyclophosphamide gavage: Weighed the cyclophosphamide sample into the mortar, added normal saline, and mixed it thoroughly. | The 60 C57BL/6 mice were divided into a low-dose group (euphorbia factor L1 6.1 mg/kg), a middle-dose group (euphorbia factor L1 18.3 mg/kg), a high-dose group (euphorbia factor L1 54.6mg/kg), a cyclophosphamide group, a model group, a blank group, 10 mice in each group, half male and half female. Except for the blank and the model group, the others were administered per day after the injection of tumor suspension, each injection was 0.2 mL. The blank group and the model group were given 2% tween-80 aqueous solution by gavage for the same time, and all were continuously administered for 10 days. |

**2 Established the H22 hepatocarcinoma mice model**

The mice in good condition after 8-13 days of tumor inoculation were put to death, and the body surface of the mice was swabbed with 75% alcohol. HCC cells were separated from mice and removed to a sterile plate containing normal saline. Put the HCC cells into the tissue homogenizer, added the physiological salt at a volume ratio of 1:3, and finally, poured the homogenate into a 50 mL centrifuge tube. Injected 0.2 mL HCC cell suspension into the right subaxillary. The process of preparing HCC cells suspension was completed under aseptic conditions; Tumor inoculation needed to be completed within 60 minutes.

**Table S3. Experimental method of anti-H22 cancer efficacy of active ingredients**

| **Compound** | **Drug preparation method** | **Anti-H22 hepatocarcinoma drug effect grouping and administration method** |
| --- | --- | --- |
| Ergosterol from *Cordyceps militaris* | Ergosterol gavage: Weighed the ergosterol sample into the mortar, added 2% Tween-80, added normal saline after thorough grinding, and mixed it thoroughly.  Cyclophosphamide gavage: Weighed the cyclophosphamide sample into the mortar, added normal saline, and mixed it thoroughly. | The 60 SD rats were divided into a low-dose group (27 mg/kg), a middle-dose group (80 mg/kg), a high-dose group (240 mg/kg), a cyclophosphamide group, a model group, a blank group, 10 mice in each group, half male and half female. Except for the blank and the model group, the others were administered per day after the injection of tumor suspension, each injection was 0.2 mL. The blank group and the model group were given 2% tween-80 aqueous solution by gavage for the same time, and all were continuously administered for 10 days. |
| Genistein from *Armillaria luteo-virens* (Aalb.et Schw:Fr.)Sacc. | Genistein gavage: Weighed the genistein sample into the mortar, added 2% Tween-80, added normal saline after thorough grinding, and mixed it thoroughly.  Cyclophosphamide gavage: After removing the film coat of the cyclophosphamide sheet, weighed the cyclophosphamide sample into the mortar, added normal saline, and mixed it thoroughly. | The 60 KM mice were divided into a low-dose group (genistein 8.19 mg/kg), a middle-dose group (genistein 24.57mg/kg), a high-dose group (genistein 73.71mg/kg), a cyclophosphamide group, a model group, a blank group, 10 mice in each group, half male and half female. Except for the blank and the model group, the others were administered per day after the injection of tumor suspension, each injection was 0.1ml/10g. The blank group and the model group were given 2% tween-80 aqueous solution by gavage for the same time, and all were continuously administered for 10 days. |
| Quercetagetin from *Tagetes erecta* L. | Quercetagetin gavage: Weighed the quercetagetin sample into the mortar, added 2% Tween-80, added normal saline after thorough grinding, and mixed it thoroughly.  Cyclophosphamide gavage: After removing the film coat of the cyclophosphamide sheet, weighed the cyclophosphamide sample into the mortar, added normal saline, and mixed it thoroughly. | The 60 KM mice were divided into low-dose quercetagetin (20 mg/kg), middle-dose quercetagetin (60mg/kg), high-dose quercetagetin (180mg/kg), a cyclophosphamide group (23mg/kg), a model group, a blank group, 10 mice in each group, half male and half female. Except for the blank and the model group, the others were administered per day after the injection of tumor suspension, each injection was 0.2 mL. The blank group and the model group were given 2% tween-80 aqueous solution by gavage for the same time, and all were continuously administered for 10 days. |
| Diphenylacetylene from *Sparganium stolonifeum* Buch.-Ham. | Diphenylacetylene gavage: Weighed the diphenylacetylene sample into the mortar, added 2% Tween-80, added normal saline after thorough grinding, and mix it thoroughly.  Cyclophosphamide gavage: After removing the film coat of the cyclophosphamide sheet, weighed the cyclophosphamide sample into the mortar, added normal saline, and mixed it thoroughly. | The 60 KM mice were divided into a low-dose group (diphenylacetylene 6.4 mg/kg), a middle-dose group (diphenylacetylene 19.2mg/kg), a high-dose group (diphenylacetylene 57.6mg/kg), a cyclophosphamide group, a model group, a blank group, 10 mice in each group, half male and half female. Except for the blank and the model group, the others were administered per day after the injection of tumor suspension, each injection was 0.1ml/10g. The blank group and the model group were given 2% tween-80 aqueous solution by gavage for the same time, and all were continuously administered for 10 days. |

**
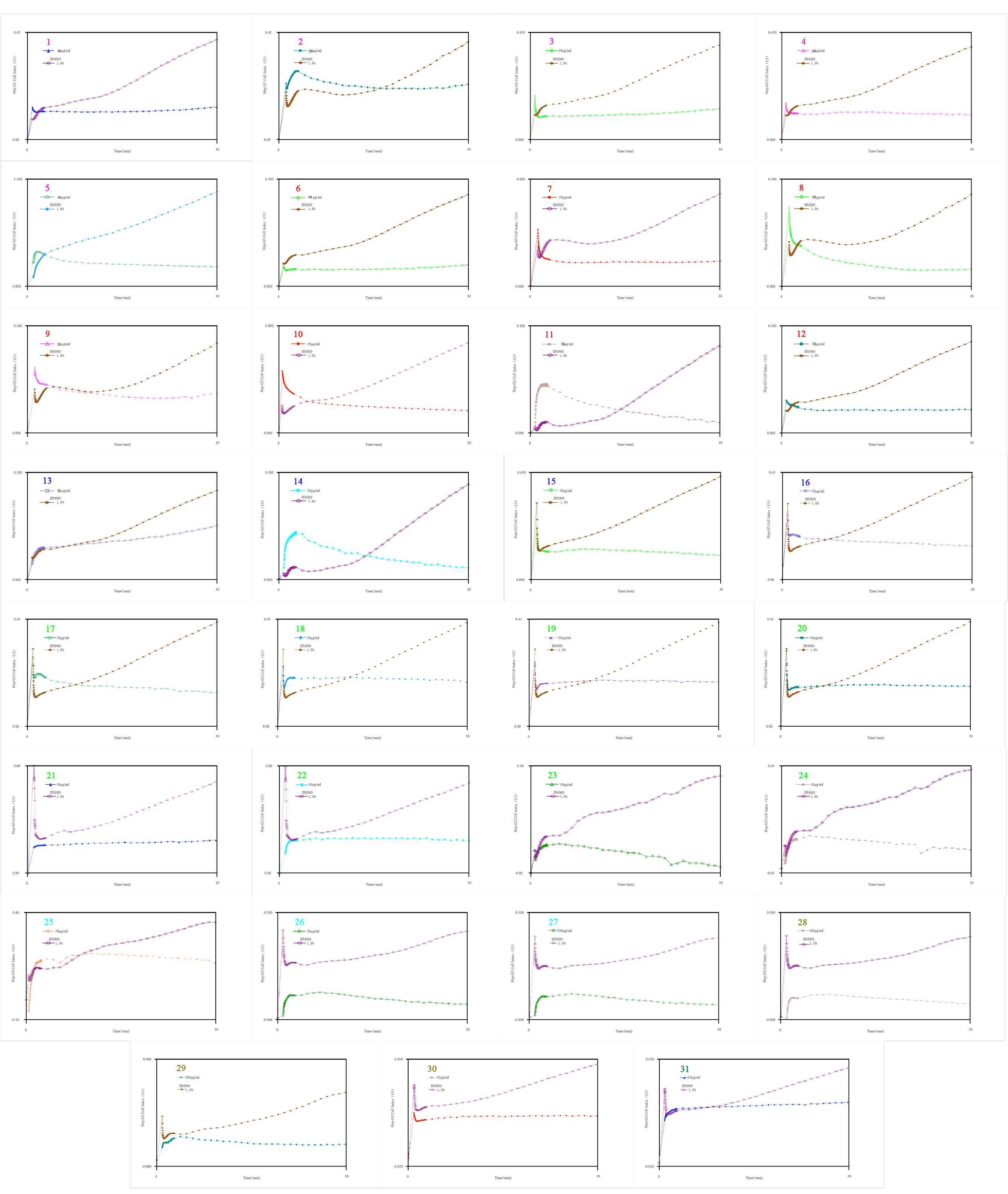
**

**Figure S1.** HepG2 cells growth trends. DMSO as the control group, the growth time was 30 min. The growth of Hep-G2 cells was inhibited by 31 compounds at the concentration of 50 μg/mL.

31 compounds were Ergosterol (**1**), Ergosta-7,22-dien-3*β*,5*α*-dihydroxy-6-one (**2**), 5*α*,8*α*-epidioxy-(22*E*,24*R*)-ergosta-6,22-dien-3*β*-ol (**3**), (24S)-5,22-stigmastadien-3*β*-ol (**4**), Ergosta-7,22-diene-3,5,6-triol (**5**), Euphorbia factor L1 (**6**), Euphorbia factor L2 (**7**), Euphorbia factor L3 (**8**), Euphorbia factor L8 (**9**), Euphorbia factor L9 (**10**), Glyceryl monooleate (**11**), esculetin (**12**), Quercetin (**13**), 6-hydroxykaempfe-rol (**14**), Protocatechuic acid (**15**), Quercetagetin (**16**), Esulone A (**17**), Kansuinin A (**18**), (3*β*, 11*β*)-3, 11-dihydroxylanosta-8, 24-dien-7-one (**19**), Kansuinin E (**20**), Kansuinin B (**21**), Isoscopoletin (**22**), Kansuinin D (**23**), Kansuinin G (**24**), Icajine (**25**), Strychnine-N-oxide (**26**), [Ursolic acid](https://www.chemsrc.com/en/cas/77-52-1_828705.html) (**27**), 3*β*-hydroxy-(22*E*, 24*R*)-ergosta-5,8,22-trien-7-one (**28**), (3*α*, 5*α*), (8*β*, 11*β*)-diepidioxy-ergost-22*E*-en-12- one (**29**), Genistein (**30**), Diphenylacetylene (**31**)


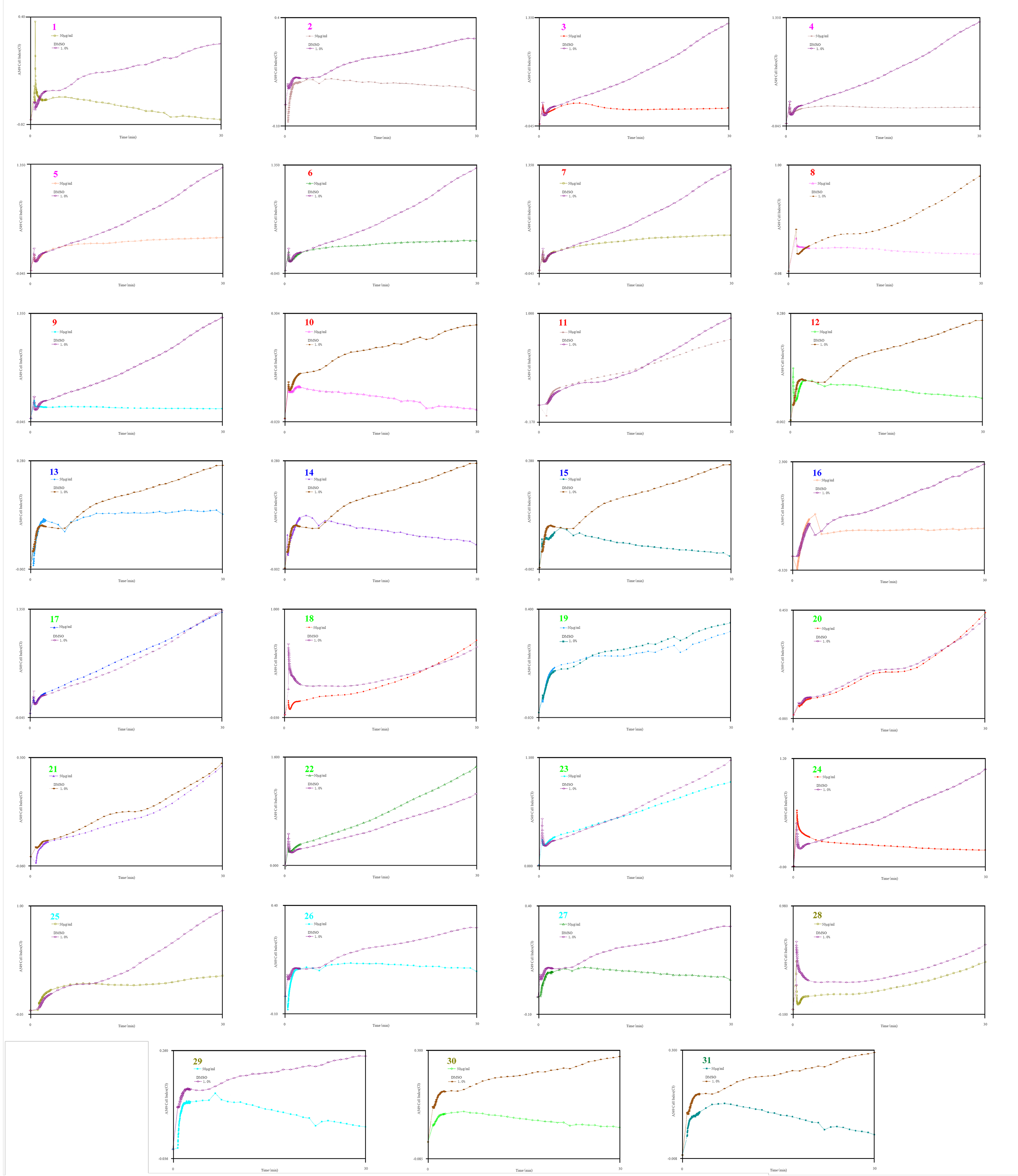


**Figure S2.** A549 cells growth trends. DMSO as the control group, the growth time was 30 min. The growth of A549 cells was inhibited by 22 compounds at the concentration of 50 μg/mL.

22 compounds were Ergosterol (**1**), Ergosta-7,22-dien-3*β*,5*α*-dihydroxy-6-one (**2**), 5*α*,8*α*-epidioxy-(22*E*,24*R*)-ergosta-6,22-dien-3*β*-ol (**3**), (24S)-5,22-stigmastadien-3*β*-ol (**4**), Ergosta-7,22-diene-3,5,6-triol (**5**), Euphorbia factor L1 (**6**), Euphorbia factor L2 (**7**), Euphorbia factor L3 (**8**), Euphorbia factor L8 (**9**), Euphorbia factor L9 (**10**), esculetin (**12**), Quercetin (**13**), 6-hydroxykaempfe-rol (**14**), Protocatechuic acid (**15**), Quercetagetin (**16**), Kansuinin G (**24**), Icajine (**25**), Strychnine-N-oxide (**26**), [Ursolic acid](https://www.chemsrc.com/en/cas/77-52-1_828705.html) (**27**), 3*β*-hydroxy-(22*E*, 24*R*)-ergosta-5,8,22-trien-7-one (**28**), (3*α*, 5*α*), (8*β*, 11*β*)-diepidioxy-ergost-22*E*-en-12- one (**29**), Genistein (**30**),

**Table S4. Drug-like statistics of 31 compounds**

| **No.** | **Name** | **Molecule ID** | **OB (%)** | **DL** | **Intestinal absorption (human)** | **CAS Registry Number** | **Molecular Formula** | **pKa (Predicted)** | **Freely Rotatable Bonds** | **H Acceptors** | **H Donors** | **H Donor/**  **Acceptor Sum** | **logP** | **Molecular Weight** |
| --- | --- | --- | --- | --- | --- | --- | --- | --- | --- | --- | --- | --- | --- | --- |
| 1 | Ergosterol | MOL000298 | 14.29 | 0.72 | 94.17 | 57-87-4 | C_28_H_44_O | 14.91±0.70 | 5 | 1 | 1 | 2 | 9.281±0.371 | 396.65 |
| 2 | Ergosta-7,22-dien-3*β*,5*α*-dihydroxy-6-one | / | / | / | 93.829 | 14858-07-2 | C_28_H_44_O_3_ | 12.35±0.70 | 6 | 3 | 2 | 5 | 6.940±0.442 | 428.65 |
| 3 | 5*α*,8*α*-epidioxy-(22*E*,24*R*)-ergosta-6,22-dien-3*β*-ol | MOL012816 | 42.84 | 0.74 | 93.205 | 2061-64-5 | C_28_H_44_O_3_ | 14.97±0.70 | 5 | 3 | 1 | 4 | 7.508±0.806 | 428.65 |
| 4 | (24*S*)-5,22-stigmastadien-3*β*-ol | MOL000449 | 43.83 | 0.76 | 95.372 | 83-48-7 | C_29_H_48_O | 15.03±0.70 | 6 | 1 | 1 | 2 | 10.072±0.296 | 412.69 |
| 5 | 22*E*,24*R*-ergosta-7,22-diene-3*β*,5*α*,6*β*-triol | */* | */* | */* | *92.86* | *392711-55-6* | *C_28_H_46_O_3_* | 13.52±0.70 | 7 | 3 | 3 | 6 | 6.714±0.406 | 430.66 |
| 6 | Euphorbia factor L1 | / | / | / | 100 | 76376-43-7 | C_32_H_40_O_8_ | 1.23±0.1 | 8 | 8 | 0 | 8 | 5.372±0.543 | 552.66 |
| 7 | Euphorbia factor L2 | / | / | / | 100 | 218916-51-9 | C_38_H_42_O_9_ | -- | 10 | 9 | 0 | 9 | 7.880±0.474 | 642.73 |
| 8 | Euphorbia factor L3 | / | / | / | 100 | 218916-52-0 | C_31_H_18_O_7_ | 1.19±0.1 | 7 | 7 | 0 | 7 | 6.930±0.442 | 522.63 |
| 9 | Euphorbia factor L8 | / | / | / | 100 | 218916-53-1 | C_30_H_37_NO_7_ | 3.10±0.10 | 7 | 8 | 0 | 8 | 5.596±0.448 | 523.62 |
| 10 | Euphorbia factor L9 | / | / | / | 100 | 129393-28-8 | C_37_H_41_NO_9_ | 3.09±0.10 | 10 | 10 | 0 | 10 | 6.546±0.479 | 643.72 |
| 11 | Glyceryl monooleate | MOL002882 | 34.13 | 0.30 | 90.741 | 111-03-5 | C_21_H_40_O_4_ | 13.16±0.20 | 21 | 4 | 2 | 6 | 6.677±0.435 | 356.54 |
| 12 | Esculetin | MOL003837 | 22.97 | 0.07 | 95.351 | 305-01-1 | C_9_H_6_O_4_ | 7.74±0.20 | 2 | 4 | 2 | 6 | 0.884±0.861 | 178.14 |
| 13 | Quercetin | MOL000098 | 46.43 | 0.28 | 75.347 | 117-39-5 | C_15_H_10_O_7_ | 6.31±0.40 | 6 | 7 | 5 | 12 | 1.989±1.075 | 302.24 |
| 14 | 6-hydroxykaempfe-rol | MOL002712 | 62.13 | 0.27 | 70.599 | 4324-55-4 | C_15_H_10_O_7_ | 6.15±0.40 | 6 | 7 | 5 | 12 | 1.930±1.076 | 302.24 |
| 15 | Protocatechuic acid | MOL000105 | 25.37 | 0.04 | 76.227 | 99-50-3 | C_7_H_6_O_4_ | 4.45±0.10 | 3 | 4 | 3 | 7 | 1.010±0.237 | 154.12 |
| 16 | Quercetagetin | MOL002721 | 45.01 | 0.31 | 68.238 | 90-18-6 | C_15_H_10_O_8_ | 6.12±0.40 | 7 | 8 | 6 | 14 | 1.234±1.286 | 318.24 |
| 17 | Esulone A | / | / | / | 95.965 | 100215-74-5 | C_38_H_42_O_12_ | 11.53±0.70 | 12 | 12 | 2 | 14 | 3.953±0.731 | 690.73 |
| 18 | Kansuinin A | MOL002593 | 44.52 | 0.55 | 96.458 | 57701-86-7 | C_37_H_46_O_15_ | 10.09±0.70 | 14 | 15 | 1 | 16 | 3.009±0.600 | 730.75 |
| 19 | (3*β*, 11*β*)-3, 11-dihydroxylanosta-8, 24-dien-7-one | / | / | / | 93.253 | 1000000-05-4 | C_30_H_48_O_3_ | 14.00±0.70 | 6 | 3 | 2 | 5 | 6.635±0.414 | 456.70 |
| 20 | Kansuinin E | / | / | / | 100 | 672945-84-5 | C_41_H_47_NO_14_ | 3.10±0.10 | 14 | 15 | 0 | 15 | 4.213±0.659 | 777.81 |
| 21 | Kansuinin B | / | / | / | 100 | 57685-46-8 | C_38_H_42_O_14_ | 11.50±0.70 | 13 | 14 | 3 | 17 | 2.547±0.795 | 722.73 |
| 22 | Isoscopoletin | MOL000339 | 23.46 | 0.08 | 95.445 | 776-86-3 | C_10_H_8_O_4_ | 8.94±0.20 | 2 | 4 | 1 | 5 | 0.992±0.792 | 192.17 |
| 23 | Kansuinin D | / | / | / | 98.902 | 672945-82-3 | C_41_H_47_NO_15_ | 10.01±0.70 | 15 | 16 | 1 | 17 | 3.862±0.609 | 793.81 |
| 24 | Kansuinin G | / | / | / | 98.41 | 770715-05-4 | C_34_H_43_NO_13_ | 12.77±0.70 | 12 | 14 | 1 | 15 | 1.834±0.643 | 673.70 |
| 25 | Icajine | / | / | / | 72.686 | 22029-96-5 | C_22_H_24_N_2_O_4_ | 12.94±0.20 | 1 | 6 | 1 | 7 | 0.486±0.824 | 380.44 |
| 26 | Strychnine-N-oxide | / | / | / | 99.321 | 7248-28-4 | C_21_H2_2_N_2_O_3_ | 4.97±0.20 | 0 | 5 | 0 | 5 | 0.337±0.887 | 350.41 |
| 27 | [Ursolic Acid](https://www.chemsrc.com/en/cas/77-52-1_828705.html) | MOL000511 | 16.77 | 0.75 | 97.377 | 77-52-1 | C_30_H_48_O_3_ | 4.68±0.70 | 2 | 3 | 2 | 5 | 8.731±0.375 | 456.70 |
| 28 | 3*β*-hydroxy-(22*E*, 24*R*)-ergosta-5,8,22-trien-7-one | / | / | / | 96.022 | 161649-84-9 | C_28_H_42_O_5_ | 1.16±0.1 | 4 | 5 | 0 | 5 | 5.086±0.856 | 458.63 |
| 29 | (3*α*, 5*α*), (8*β*, 11*β*)-diepidioxy-ergost-22*E*-en-12- one | / | / | / | 94.56 | 200942-18-3 | C_28_H_42_O_2_ | 14.57±0.70 | 5 | 2 | 1 | 3 | 6.843±0.407 | 410.63 |
| 30 | Genistein | MOL000481 | 17.93 | 0.21 | 92.67 | 446-72-0 | C_15_H_10_O_5_ | 6.51±0.20 | 4 | 5 | 3 | 8 | 3.114±1.137 | 270.24 |
| 31 | Diphenylacetylene | / | 0.55 | / | 94.268 | 955955-70-1 | C_16_H_12_O_5_ | 8.59±0.50 | 6 | 5 | 3 | 8 | 5.124±0.794 | 284.26 |

**Table S5. Annotations the functions of key targets**

| **GeneID** | **annotation** | **Degrees** |
| --- | --- | --- |
| AKT1 | RAC-alpha serine/threonine-protein kinase | 43 |
| SRC | Proto-oncogene tyrosine-protein kinase Src | 41 |
| CCND1 | G1/S-specific cyclin-D1 | 38 |
| MAPK3 | Mitogen-activated protein kinase 3 | 38 |
| PIK3R1 | Phosphatidylinositol 3-kinase regulatory subunit alpha | 38 |
| IL6 | Interleukin-6 | 36 |
| EGFR | Epidermal growth factor receptor | 34 |
| MAPK8 | Mitogen-activated protein kinase 8 | 33 |
| APP | Amyloid-beta A4 protein | 32 |
| AR | Androgen receptor | 31 |
| CDK1 | Cyclin-dependent kinase 1 | 29 |
| CASP3 | Caspase-3 | 28 |
| ESR1 | Estrogen receptor | 26 |
| MAPK14 | Mitogen-activated protein kinase 14 | 26 |
| CCNB1 | G2/mitotic-specific cyclin-B1 | 24 |
| ERBB2 | Receptor tyrosine-protein kinase erbB-2 | 24 |
| MMP9 | Matrix metalloproteinase-9 | 24 |
| CDK2 | Cyclin-dependent kinase 2 | 23 |
| PTGS2 | Prostaglandin G/H synthase 2 | 23 |
| CCNA2 | Cyclin-A2 | 22 |
| JAK1 | Tyrosine-protein kinase JAK1 | 20 |
| BCL2L1 | Bcl-2-like protein 1 | 19 |
| AGTR1 | Type-1 angiotensin II receptor | 18 |
| CDC25A | M-phase inducer phosphatase 1 | 18 |
| CDK5 | Cyclin-dependent-like kinase 5 | 18 |
| F2 | Prothrombin | 18 |
| MAP2K1 | Dual specificity mitogen-activated protein kinase kinase 1 | 17 |
| PTK2 | Focal adhesion kinase 1 | 16 |
| CASP8 | Caspase-8 | 15 |
| NR3C1 | Glucocorticoid receptor | 15 |
| GSK3B | Glycogen synthase kinase-3 beta | 14 |
| HSP90AB1 | Heat shock protein HSP 90-beta | 14 |
| PRKCD | Protein kinase C delta type | 14 |

**Table S6. The crystal structure information of targets**

|  | **Gene ID** | **PDB ID** | **Positions** | **URL** | **bound ligand** | **Chain** |
| --- | --- | --- | --- | --- | --- | --- |
| 1 | EGFR | 1M17 | 695-1022 | https://www.ebi.ac.uk/pdbe/entry/pdb/1M17 | AQ4 | A |
| 2 | AKT1 | 6S9W | 2-446 | https://www.ebi.ac.uk/pdbe/entry/pdb/6S9W | L1Z | A |
| 3 | AR | 5V8Q | 672-920 | https://www.ebi.ac.uk/pdbe/entry/pdb/5V8Q | 97A | A |
| 4 | MAPK3 | 4QTB | 1-379 | https://www.ebi.ac.uk/pdbe/entry/pdb/4QTB | 38Z | A/B |
| 5 | ESR1 | 1GWQ | 301-548 | https://www.ebi.ac.uk/pdbe/entry/pdb/1GWQ | ZTW | A/B |
| 6 | IL6 | 1ALU | 28-212 | https://www.ebi.ac.uk/pdbe/entry/pdb/1ALU | TLA | A |
| 7 | PIK3R1 | 5M6U | 1-724 | https://www.ebi.ac.uk/pdbe/entry/pdb/5M6U | 7KA | B |
| 8 | CDK1 | 5LQF | 1-297 | https://www.ebi.ac.uk/pdbe/entry/pdb/5LQF | 4SP | A/D |
| 9 | CDK2 | 6Q4H | 1-298 | https://www.ebi.ac.uk/pdbe/entry/pdb/6Q4H | MBP | A |
| 10 | CCNB1 | 6GU2 | 165-433 | https://www.ebi.ac.uk/pdbe/entry/pdb/6GU2 | F9Z | B |
| 11 | CDK5 | 3O0G | 1-292 | https://www.ebi.ac.uk/pdbe/entry/pdb/3O0G | 3O0 | A/B |


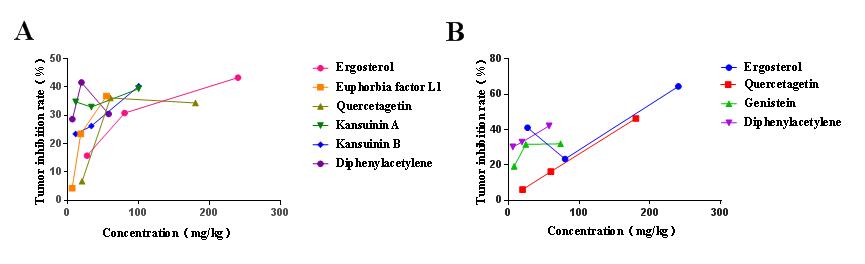


**Figure S3. Comparison the efficacy of active compounds**

**(A)** The inhibitory rate of the compound on Lewis mice. Diphenylacetylene had a relatively strong inhibition rate on Lewis mice, followed by euphorbia factor L1 and ergosterol. the inhibition rate of ergosterol, euphorbia factor L1, and kansuinin B on Lewis mice increased with rising the dose, while the inhibition rate of quercetagetin, kansuinin A, and diphenylacetylene were not affected by the dose. When the inhibition rate was 40%, the dose were diphenylacetylene, euphorbia factor L1, and ergosterol from low to high. (**B)** The inhibitory rate of the compound on H22 mice. Ergosterol had a relatively strong inhibition rate on H22 mice, followed by diphenylacetylene and quercetagetin. When the inhibition rate was 40%, the dose were ergosterol, diphenylacetylene, and quercetagetin from low to high.

**Reference**

[1] Chen Y, Wu Y, Li S, *et al*. Large-scale isolation and antitumor mechanism evaluation of compounds from the traditional Chinese medicine Cordyceps Militaris[J]. *Eur J Med Chem*. 2021;212:113142.

**Table S7. Mutant enzymes in A549 cells**

| **No.** | **cDNA** | **Identities（%）** | **Sequence ID** | **Proteins** |
| --- | --- | --- | --- | --- |
| 1 | Query= c100346/f1p0/1148 | 98 | XP_005273594.1 | PREDICTED: DNA polymerase beta isoform X3 [Homo sapiens] |
| 2 | Query= c100538/f1p2/1601 | 95 | NP_005947.3 | C-1-tetrahydrofolate synthase, cytoplasmic [Homo sapiens] |
| 3 | Query= c100768/f1p7/1118 | 96 | NP_612510.1 | very-long-chain enoyl-CoA reductase isoform 1 [Homo sapiens] |
| 4 | Query= c10249/f1p3/1744 | 93 | NP_008975.1 | katanin p60 ATPase-containing subunit A1 isoform 1 [Homo sapiens] |
| 5 | Query= c10450/f1p2/3051 | 94 | NP_004642.2 | ubiquitin carboxyl-terminal hydrolase 11 [Homo sapiens] |
| 6 | Query= c10602/f1p1/754 | 87 | NP_076869.1 | vitamin K epoxide reductase complex subunit 1 isoform 1 precursor [Homo sapiens] |
| 7 | Query= c106179/f1p35/1320 | 88 | NP_006494.1 | 26S protease regulatory subunit 6B isoform 1 [Homo sapiens] |
| 8 | Query= c10685/f7p11/983 | 98 | XP_008962764.1 | PREDICTED: ADP-ribosylation factor-like protein 6-interacting protein 4 isoform X3 [Pan paniscus] |
|  |  | 87 | AAH15909.2 | ADP-ribosylation-like factor 6 interacting protein 4 [Homo sapiens] |
| 9 | Query= c11248/f2p3/986 | 99 | XP_004060479.1 | PREDICTED: cytochrome b-c1 complex subunit Rieske, mitochondrial-like isoform 2 [Gorilla gorilla gorilla] |
|  |  | 93 | NP_005994.2 | cytochrome b-c1 complex subunit Rieske, mitochondrial [Homo sapiens] |
| 10 | Query= c11639/f1p3/875 | 92 | NP_004641.1 | patatin-like phospholipase domain-containing protein 4 isoform 1 precursor [Homo sapiens] |
| 11 | Query= c13248/f2p2/2548 | 98 | NP_055078.1 | ATP-dependent zinc metalloprotease YME1L1 isoform 3 [Homo sapiens] |
| 12 | Query= c13280/f1p1/1683 | 94 | NP_000174.1 | trifunctional enzyme subunit beta, mitochondrial isoform 1 precursor [Homo sapiens] |
| 13 | Query= c13539/f2p1/2110 | 93 | NP_817092.1 | protein phosphatase 1G [Homo sapiens] |
| 14 | Query= c13575/f3p11/1226 | 93 | AAH50082.1 | TIMM50 protein, partial [Homo sapiens] |
| 15 | Query= c13731/f1p1/2508 | 91 | NP_001075.1 | procollagen-lysine,2-oxoglutarate 5-dioxygenase 3 precursor [Homo sapiens] |
| 16 | Query= c13875/f4p0/956 | 83 | NP_002504.2 | nucleoside diphosphate kinase 3 precursor [Homo sapiens] |
| 17 | Query= c13968/f2p2/1381 | 87 | NP_115725.1 | protein syndesmos isoform 1 [Homo sapiens] |
| 18 | Query= c14046/f1p1/1007 | 98 | XP_012355209.1 | PREDICTED: ubiquitin carboxyl-terminal hydrolase isozyme L1 isoform X6 [Nomascus leucogenys] |
|  |  | 91 | NP_004172.2 | ubiquitin carboxyl-terminal hydrolase isozyme L1 [Homo sapiens] |
| 19 | Query= c14118/f2p2/2217 | 97 | NP_057081.3 | protein RRNAD1 isoform 1 [Homo sapiens] |
| 20 | Query= c14213/f1p1/1321 | 97 | NP_000013.2 | adenosine deaminase isoform 1 [Homo sapiens] |
| 21 | Query= c14225/f1p2/489 | 79 | NP_002487.1 | NADH dehydrogenase [ubiquinone] iron-sulfur protein 8, mitochondrial precursor [Homo sapiens] |
| 22 | Query= c14471/f1p1/561 | 76 | XP_017196591.1 | PREDICTED: NADH dehydrogenase [ubiquinone] 1 alpha subcomplex subunit 2 isoform X1 [Oryctolagus cuniculus] |
|  |  | 72 | NP_002479.1 | NADH dehydrogenase [ubiquinone] 1 alpha subcomplex subunit 2 isoform 1 [Homo sapiens] |
| 23 | Query= c14899/f1p0/1026 | 80 | EAW57572.1 | mitochondrial rRNA methyltransferase 1 homolog (S. cerevisiae), isoform CRA_b [Homo sapiens] |
| 24 | Query= c15011/f1p6/1552 | 91 | AAH11907.1 | Dipeptidyl-peptidase 7 [Homo sapiens] |
| 25 | Query= c15226/f1p4/1213 | 89 | NP_060311.1 | succinate dehydrogenase assembly factor 2, mitochondrial [Homo sapiens] |
| 26 | Query= c15813/f1p5/1900 | 88 | NP_005756.2 | renin receptor precursor [Homo sapiens] |
| 27 | Query= c16077/f1p0/635 | 83 | NP_001193577.1 | A-kinase-interacting protein 1 isoform d [Homo sapiens] |
| 28 | Query= c16205/f1p1/1096 | 86 | XP_008055095.1 | PREDICTED: DNA-directed RNA polymerases I, II, and III subunit RPABC1 [Carlito syrichta] |
|  |  | 77 | AAH34144.1 | Polymerase (RNA) II (DNA directed) polypeptide E, 25kDa [Homo sapiens] |
| 29 | Query= c16355/f2p0/1055 | 76 | NP_001291762.1 | 2-oxoglutarate and iron-dependent oxygenase domain-containing protein 2 Isoform 1 [Homo sapiens] |
| 30 | Query= c16781/f1p6/2322 | 81 | BAD96546.1 | methylene tetrahydrofolate dehydrogenase 2 precursor variant, partial [Homo sapiens] |
| 31 | Query= c16833/f1p4/1691 | 77 | NP_002927.2 | ribonuclease H1 isoform 1 precursor [Homo sapiens] |
| 32 | Query= c16946/f1p2/1079 | 94 | AAI21148.1 | Translocase of inner mitochondrial membrane 50 homolog (S. cerevisiae) [Homo sapiens] |
| 33 | Query= c16949/f1p8/1216 | 92 | NP_001015624.1 | protein arginine N-methyltransferase 1 [Bos taurus] |
|  |  | 92 | AAH19268.2 | PRMT1 protein, partial [Homo sapiens] |
| 34 | Query= c16954/f1p7/1105 | 94 | NP_004993.1 | NADH dehydrogenase [ubiquinone] 1 alpha subcomplex subunit 9, mitochondrial precursor [Homo sapiens] |
| 35 | Query= c16986/f1p8/1179 | 93 | NP_055567.2 | signal peptidase complex subunit 2 [Homo sapiens] |
| 36 | Query= c17203/f1p0/1036 | 85 | NP_036335.1 | glyoxylate reductase/  hydroxypyruvate redu-  ctase [Homo sapiens] |
| 37 | Query= c17910/f1p0/974 | 78 | NP_001185891.1 | enoyl-CoA hydratase domain-containing protein 2, mitochondrial isoform 3 [Homo sapiens] |
| 38 | Query= c17973/f1p0/694 | 100 | XP_009003032.1 | PREDICTED: ribonucl-  ease P/MRP protein subunit POP5 isoform X3 [Callithrix jacchus] |
|  |  | 80 | NP_057002.2 | ribonuclease P/MRP protein subunit POP5 isoform a [Homo sapiens] |
| 39 | Query= c18381/f9p6/1231 | 83 | NP_003304.1 | GDP-L-fucose synthase isoform 2 [Homo sapiens] |
| 40 | Query= c18471/f2p0/709 | 92 | NP_001304983.1 | GTPase HRas isoform 3 precursor [Homo sapiens] |
| 41 | Query= c18668/f9p10/913 | 88 | NP_006432.1 | 5-formyltetrahydrofolate cyclo-ligase isoform a [Homo sapiens] |
| 42 | Query= c18756/f1p3/922 | 84 | NP_004038.1 | V-type proton ATPase 21 kDa proteolipid subunit isoform 1 [Homo sapiens] |
| 43 | Query= c18854/f1p7/1932 | 90 | AAT08032.1 | growth-inhibiting protein 18 [Homo sapiens] |
| 44 | Query= c19037/f1p0/1192 | 89 | NP_001288719.1 | methyltransferase-like protein 6 isoform 2 [Homo sapiens] |
| 45 | Query= c19055/f5p1/1433 | 100 | XP_008961373.1 | PREDICTED: UDP-glucose 4-epimerase isoform X2 [Pan paniscus] |
|  |  | 92 | NP_001008217.1 | UDP-glucose 4-epim-erase [Homo sapiens] |
| 46 | Query= c19203/f2p1/1042 | 82 | NP_115648.2 | repressor of RNA polymerase III transcription MAF1 homolog [Homo  sapiens] |
| 47 | Query= c19208/f1p1/1196 | 87 | NP_000145.1 | galactokinase [Homo sapiens] |
| 48 | Query= c19566/f1p2/1822 | 79 | ABD90542.1 | retinol dehydrogenase 11, partial [Homo sapiens] |
| 49 | Query= c19683/f2p1/1967 | 91 | NP_001136026.1 | tRNA (guanine(26)-N(2))-dimethyltransferase isoform 2 [Homo sapiens] |
| 50 | Query= c19784/f1p0/614 | 93 | NP_004539.1 | NADH dehydrogenase [ubiquinone] 1 beta subcomplex subunit 10 [Homo sapiens] |
| 51 | Query= c2152/f4p3/935 | 99 | XP_008954755.1 | PREDICTED: ADP-ribosylation factor-like protein 16 isoform X2 [Pan paniscus] |
|  |  | 76 | EAW89671.1 | hCG1991579, isoform CRA_a, partial [Homo sapiens] |
| 52 | Query= c21811/f2p4/1677 | 95 | NP_000300.1 | protoporphyrinogen oxidase [Homo sapiens] |
| 53 | Query= c21845/f1p4/2061 | 86 | NP_001288130.1 | tribbles homolog 3 isoform 2 [Homo sapiens] |
| 54 | Query= c21848/f1p0/1094 | 91 | XP_005257303.1 | PREDICTED: endonuclease V isoform X14 [Homo sapiens] |
| 55 | Query= c21921/f2p5/1550 | 100 | XP_004034156.1 | PREDICTED: inosine-5'-monophosphate dehydrogenase 2 isoform 2 [Gorilla gorilla gorilla] |
|  |  | 95 | NP_000875.2 | inosine-5'-monophosphate dehydrogenase 2 [Homo sapiens] |
| 56 | Query= c22055/f2p2/2293 | 79 | XP_011523034.1 | PREDICTED: estradiol 17-beta-dehydrogenase 1 isoform X8 [Homo sapiens] |
| 57 | Query= c22620/f1p0/1783 | 82 | XP_011534689.1 | PREDICTED: trans-3-hydroxy-L-proline dehydratase isoform X2 [Homo sapiens] |
| 58 | Query= c22848/f1p7/1052 | 62 | XP_011537401.1 | PREDICTED: mitochondrial import inner membrane translocase subunit Tim23 isoform X2 [Homo sapiens] |
| 59 | Query= c22877/f1p8/550 | 60 | NP_001335.1 | dolichyl-diphosphooligosaccharide--protein glycosyltransferase subunit DAD1 [Homo sapiens] |
| 60 | Query= c23192/f2p0/502 | 83 | NP_006224.1 | DNA-directed RNA polymerase II subunit RPB9 [Homo sapiens] |
| 61 | Query= c23204/f2p2/831 | 87 | NP_009193.2 | protein deglycase DJ-1 [Homo sapiens] |
| 62 | Query= c23293/f1p4/880 | 84 | NP_066996.3 | DNA polymerase delta subunit 4 isoform 1 [Homo sapiens] |
| 63 | Query= c23898/f1p2/558 | 67 | XP_009426340.1 | PREDICTED: cytochrome c oxidase assembly protein COX16 homolog, mitochondrial isoform X1 [Pan troglodytes] |
|  |  | 56 | NP_057552.1 | cytochrome c oxidase assembly protein COX16 homolog, mitochondrial isoform 1 precursor [Homo sapiens] |
| 64 | Query= c24295/f1p1/1728 | 86 | EAW65568.1 | three prime histone mRNA exonuclease 1, isoform CRA_a [Homo sapiens] |
| 65 | Query= c24379/f2p1/1588 | 92 | XP_005266862.1 | PREDICTED: katanin p60 ATPase-containing subunit A1 isoform X2 [Homo sapiens] |
| 66 | Query= c24557/f1p2/1646 | 96 | XP_015797082.1 | PREDICTED: serine/threonine-protein phosphatase PP2A-like [Nothobranchius furzeri] |
|  |  | 73 | XP_010638022.1 | PREDICTED: serine/threonine-protein phosphatase 2A catalytic subunit alpha isoform [Fukomys damarensis] |
| 67 | Query= c24647/f1p0/810 | 93 | XP_011526530.1 | PREDICTED: thimet oligopeptidase isoform X1 [Homo sapiens] |
| 68 | Query= c24879/f1p2/2651 | 93 | NP_001182666.1 | prostamide/prostaglandin F synthase isoform c [Homo sapiens] |
| 69 | Query= c24946/f1p2/2112 | 88 | NP_001309316.1 | ectonucleoside triphosphate diphosphohydrolase 6 isoform 9 [Homo sapiens] |
| 70 | Query= c25098/f1p3/2082 | 79 | NP_036238.1 | ADP-ribosylation factor-like protein 2-binding protein [Homo sapiens] |
| 71 | Query= c25583/f1p8/1154 | 100 | XP_006050557.1 | PREDICTED: DNA-directed RNA polymerases I, II, and III subunit RPABC1 isoform X2 [Bubalus bubalis] |
|  |  | 86 | NP_002686.2 | DNA-directed RNA polymerases I, II, and III subunit RPABC1 isoform a [Homo sapiens] |
| 72 | Query= c26020/f1p0/1989 | 91 | NP_061896.1 | protein arginine N-methyltransferase 7 isoform 1 [Homo sapiens] |
| 73 | Query= c26040/f1p1/1298 | 84 | NP_000128.1 | fumarylacetoacetase [Homo sapiens] |
| 74 | Query= c26163/f2p2/1367 | 99 | XP_009183818.1 | PREDICTED: CAAX prenyl protease 2 isoform X2 [Papio anubis] |
|  |  | 93 | NP_005124.1 | CAAX prenyl protease 2 isoform 1 [Homo sapiens] |
| 75 | Query= c26288/f2p3/1499 | 89 | NP_057095.3 | trans-2-enoyl-CoA reductase, mitochondrial isoform a [Homo sapiens] |
| 76 | Query= c2661/f1p3/925 | 72 | EAW96364.1 | microsomal glutathione S-transferase 1, isoform CRA_c [Homo sapiens] |
| 77 | Query= c26787/f1p2/1350 | 93 | AAH00723.1 | Carnitine acetyltransferase [Homo sapiens] |
| 78 | Query=c29904/f6p53/758 | 98 | EAW68357.1 | zinc finger, DHHC-type containing 13, isoform CRA_a [Homo sapiens] |
| 79 | Query=c30500/f4p18/1266 | 95 | BAA07919.1 | 26S proteasome subunit p45 [Homo sapiens] |
| 80 | Query=c30743/f1p0/1430 | 97 | NP_005795.2 | ATP-dependent RNA helicase DDX39A [Homo sapiens] |
| 81 | Query=c31022/f1p2/894 | 89 | XP_015001672.1 | PREDICTED: repressor of RNA polymerase III transcription MAF1 homolog isoform X1 [Macaca mulatta] |
|  |  | 76 | NP_115648.2 | repressor of RNA polymerase III transcription MAF1 homolog [Homo sapiens] |
| 82 | Query=c31090/f1p0/1241 | 99 | XP_008957719.1 | PREDICTED: alpha-tubulin N-acetyltransferase 1 isoform X3 [Pan paniscus] |
|  |  | 93 | NP_001026892.1 | alpha-tubulin N-acetyltransferase 1 isoform 1 precursor [Homo sapiens] |
| 83 | Query=c31104/f1p0/2590 | 82 | AAF81404.1 | phosphatidylinositol polyphosphate 5-phosphatase type IV [Homo sapiens] |
| 84 | Query=c31464/f1p6/676 | 79 | NP_001274271.1 | 14 kDa phosphohistidine phosphatase isoform 4 [Homo sapiens] |
| 85 | Query=c31600/f1p0/2020 | 81 | NP_659489.1 | uracil phosphoribosyltransferase homolog isoform 1 [Homo sapiens] |
| 86 | Query=c31922/f1p4/962 | 86 | NP_658985.2 | NAD(P)H-hydrate epimerase precursor [Homo sapiens] |
| 87 | Query=c3207/f1p0/562 | 71 | AAA02882.1 | Miller-Dieker lissencephaly protein [Homo sapiens] |
| 88 | Query=c32171/f1p1/872 | 100 | XP_014197370.1 | PREDICTED: peroxisomal coenzyme A diphosphatase NUDT7 isoform X2 [Pan paniscus] |
|  |  | 97 | NP_001230590.1 | peroxisomal coenzyme A diphosphatase NUDT7 isoform 2 [Homo sapiens] |
| 89 | Query=c32402/f1p3/2231 | 89 | NP_001339.1 | death-associated protein kinase 3 [Homo sapiens] |
| 90 | Query=c32406/f1p1/2695 | 84 | XP_005270960.1 | PREDICTED: nardilysin isoform X3 [Homo sapiens] |
| 91 | Query=c32710/f1p0/1861 | 95 | NP_036369.2 | NAD-dependent protein deacetylase sirtuin-2 isoform 1 [Homo sapiens] |
| 92 | Query=c32971/f1p1/1511 | 94 | NP_001124313.1 | RNA 3'-terminal phosphate cyclase isoform a [Homo sapiens] |
| 93 | Query=c33337/f1p4/1265 | 90 | NP_065108.1 | fructose-2,6-bisphosphatase TIGAR [Homo sapiens] |
| 94 | Query=c34030/f1p0/1942 | 96 | NP_001138497.1 | ER degradation-enhancing alpha-mannosidase-like protein 2 isoform 2 precursor [Homo sapiens] |
| 95 | Query=c34084/f1p0/1287 | 99 | XP_011726190.1 | PREDICTED: integrin-linked kinase-associated serine/threonine phosphatase 2C isoform X2 [Macaca nemestrina] |
|  |  | 89 | NP_110395.1 | integrin-linked kinase-associated serine/threonine phosphatase 2C [Homo sapiens] |
| 96 | Query=c34212/f1p1/2333 | 95 | XP_011536994.1 | PREDICTED: acetoacetyl-CoA synthetase isoform X1 [Homo sapiens] |
| 97 | Query=c34777/f1p3/1790 | 82 | NP_061982.3 | chitobiosyldiphosphodolichol beta-mannosyltransferase isoform 1 [Homo sapiens] |
| 98 | Query=c34854/f1p1/1605 | 94 | NP_065715.1 | peroxisomal 2,4-dienoyl-CoA reductase [Homo sapiens] |
| 99 | Query=c35460/f1p7/2100 | 91 | NP_005539.1 | lysine--tRNA ligase isoform 2 [Homo sapiens] |
| 100 | Query=c35470/f1p5/1085 | 86 | XP_011515135.1 | PREDICTED: NADH dehydrogenase (ubiquinone) complex I, assembly factor 6 isoform X1 [Homo sapiens] |
| 101 | Query=c35500/f1p2/1246 | 89 | NP_660160.2 | dehydrogenase/reductase SDR family member on chromosome X precursor [Homo sapiens] |
| 102 | Query=c3824/f1p4/771 | 76 | NP_001025189.1 | adenine phosphoribosyltransferase isoform b [Homo sapiens] |
| 103 | Query=c38696/f1p4/1879 | 91 | NP_001268441.1 | trifunctional enzyme subunit beta, mitochondrial isoform 2 precursor [Homo sapiens] |
| 104 | Query=c38967/f1p2/1199 | 87 | NP_060277.1 | probable tRNA N6-adenosine threonylcarbamoyltransferase [Homo sapiens] |
| 105 | Query=c39336/f1p5/1298 | 94 | AAA35518.1 | aldehyde dehydrogenase I, partial [Homo sapiens] |
| 106 | Query=c39945/f1p3/1405 | 94 | NP_001310965.1 | uroporphyrinogen-III synthase isoform 1 [Homo sapiens] |
| 107 | Query=c4001/f1p1/1926 | 94 | NP_073597.2 | PC-esterase domain-containing protein 1A isoform 1 [Homo sapiens] |
| 108 | Query=c40254/f1p2/1853 | 94 | NP_002807.1 | 26S proteasome non-ATPase regulatory subunit 12 isoform 1 [Homo sapiens] |
| 109 | Query=c40389/f1p9/1891 | 86 | NP_079483.3 | FAD synthase isoform 1 [Homo sapiens] |
| 110 | Query=c40446/f1p4/630 | 82 | XP_001152516.2 | PREDICTED: glutathione S-transferase P [Pan troglodytes] |
|  |  | 82 | NP_000843.1 | glutathione S-transferase P [Homo sapiens] |
| 111 | Query=c40609/f1p6/1191 | 100 | XP_004028199.1 | PREDICTED: mitochondrial import inner membrane translocase subunit Tim17-A [Gorilla gorilla gorilla] |
|  |  | 75 | CAD29856.1 | TPA: mitochondrial inner membrane translocase [Homo sapiens] |
| 112 | Query=c40920/f1p1/1924 | 94 | CAA52291.1 | Lon protease-like protein [Homo sapiens] |
| 113 | Query=c4130/f1p0/2072 | 97 | XP_006064761.1 | PREDICTED: caseinolytic peptidase B protein homolog isoform X3 [Bubalus bubalis] |
|  |  | 95 | NP_001245322.1 | caseinolytic peptidase B protein homolog isoform 3 [Homo sapiens] |
| 114 | Query=c41594/f1p3/1482 | 83 | NP_001304712.1 | GDP-L-fucose synthase isoform 1 [Homo sapiens] |
| 115 | Query=c42046/f1p2/1034 | 96 | NP_001313535.1 | aminoacyl tRNA synthase complex-interacting multifunctional protein 2 isoform b [Homo sapiens] |
| 116 | Query=c42604/f1p0/1443 | 94 | XP_016870338.1 | PREDICTED: methylglutaconyl-CoA hydratase, mitochondrial isoform X3 [Homo sapiens] |
| 117 | Query=c43460/f1p0/1258 | 91 | XP_009242598.1 | PREDICTED: galactose-1-phosphate uridylyltransferase isoform X2 [Pongo abelii] |
|  |  | 86 | EAW58430.1 | hCG2040046, isoform CRA_c [Homo sapiens] |
| 118 | Query=c44122/f1p0/776 | 83 | NP_006404.1 | ribonuclease P protein subunit p30 isoform b [Homo sapiens] |
| 119 | Query=c44569/f2p0/2122 | 86 | NP_003792.1 | glycosylphosphatidylinositol anchor attachment 1 protein [Homo sapiens] |
| 120 | Query=c44642/f1p12/1075 | 96 | EAW53696.1 | guanine nucleotide binding protein (G protein), beta polypeptide 2-like 1, isoform CRA_e [Homo sapiens] |
| 121 | Query=c48737/f1p3/747 | 66 | NP_057560.8 | anaphase-promoting complex subunit 11 isoform 2 [Homo sapiens] |
| 122 | Query=c49020/f1p3/1712 | 91 | XP_011521882.1 | PREDICTED: NAD-dependent protein deacetylase sirtuin-7 isoform X1 [Homo sapiens] |
| 123 | Query=c49291/f1p4/724 | 76 | NP_006423.1 | epididymal secretory protein E1 precursor [Homo sapiens] |
| 124 | Query=c49869/f1p1/881 | 88 | NP_001165459.1 | N(4)-(beta-N-acetylglucosaminyl)-L-asparaginase isoform 2 precursor [Homo sapiens] |
| 125 | Query=c50074/f1p0/1810 | 100 | XP_011891135.1 | PREDICTED: cyclin-dependent kinase-like 5 isoform X2 [Cercocebus atys] |
|  |  | 96 | NP_001310218.1 | cyclin-dependent kinase-like 5 isoform 2 [Homo sapiens] |
| 126 | Query=c50188/f1p2/1792 | 91 | NP_116246.2 | serine beta-lactamase-like protein LACTB, mitochondrial isoform a precursor [Homo sapiens] |
| 127 | Query=c50935/f2p3/1356 | 93 | XP_008573609.1 | PREDICTED: protein arginine N-methyltransferase 1 isoform X2 [Galeopterus variegatus] |
|  |  | 93 | AAH19268.2 | PRMT1 protein, partial [Homo sapiens] |
| 12 | Query=c50987/f2p2/600 | 84 | NP_783313.1 | NADH dehydrogenase [ubiquinone] 1 alpha subcomplex subunit 11 isoform 1 [Homo sapiens] |
| 129 | Query=c51075/f1p2/1006 | 87 | XP_011515182.1 | PREDICTED: 2,4-dienoyl-CoA reductase, mitochondrial isoform X2 [Homo sapiens] |
| 130 | Query=c51125/f1p2/779 | 64 | NP_036592.1 | mitochondrial import inner membrane translocase subunit Tim9 isoform a [Homo sapiens] |
| 131 | Query=c51139/f1p1/1237 | 92 | XP_006721900.1 | PREDICTED: endonuclease V isoform X9 [Homo sapiens] |
| 132 | Query=c53316/f2p1/959 | 87 | EAW89243.1 | 5', 3'-nucleotidase, cytosolic, isoform CRA_c [Homo sapiens] |
| 133 | Query=c53699/f3p5/966 | 100 | XP_008954755.1 | PREDICTED: ADP-ribosylation factor-like protein 16 isoform X2 [Pan paniscus] |
|  |  | 79 | NP_001035114.1 | ADP-ribosylation factor-like protein 16 isoform 1 [Homo sapiens] |
| 134 | Query=c56793/f1p3/1474 | 99 | XP_007963073.1 | PREDICTED: kynureninase isoform X2 [Chlorocebus sabaeus] |
|  |  | 89 | NP_003928.1 | kynureninase isoform a [Homo sapiens] |
| 135 | Query=c57074/f1p1/1267 | 99 | XP_007483275.1 | PREDICTED: CTD nuclear envelope phosphatase 1 isoform X2 [Monodelphis domestica] |
|  |  | 90 | NP_056158.2 | CTD nuclear envelope phosphatase 1 [Homo sapiens] |
| 136 | Query=c57311/f1p3/468 | 78 | NP_002406.1 | macrophage migration inhibitory factor [Homo sapiens] |
| 137 | Query=c57314/f1p11/3381 | 68 | NP_001304699.1 | mediator of RNA polymerase II transcription subunit 29 isoform 2 [Homo sapiens] |
| 138 | Query=c57718/f1p3/2548 | 92 | NP_065116.3 | xaa-Pro aminopeptidase 1 isoform 1 [Homo sapiens] |
| 139 | Query=c58954/f1p7/1119 | 93 | NP_003730.4 | aldo-keto reductase family 1 member C3 isoform 1 [Homo sapiens] |
| 140 | Query=c59542/f1p1/720 | 80 | NP_005993.1 | ubiquitin carboxyl-terminal hydrolase isozyme L3 isoform 2 [Homo sapiens] |
| 141 | Query=c60669/f2p0/1345 | 99 | XP_008957719.1 | PREDICTED: alpha-tubulin N-acetyltransferase 1 isoform X3 [Pan paniscus] |
|  |  | 93 | NP_001026892.1 | alpha-tubulin N-acetyltransferase 1 isoform 1 precursor [Homo sapiens] |
| 142 | Query=c60997/f1p6/1242 | 67 | NP_057673.2 | complex I assembly factor TIMMDC1, mitochondrial precursor [Homo sapiens] |
| 143 | Query=c65789/f3p2/1065 | 77 | EAW89691.1 | procollagen-proline, 2-oxoglutarate 4-dioxygenase (proline 4-hydroxylase), beta polypeptide, isoform CRA_b [Homo sapiens] |
| 144 | Query=c66004/f1p2/1240 | 87 | XP_011785139.1 | PREDICTED: 7-methylguanosine phosphate-specific 5'-nucleotidase isoform X1 [Colobus angolensis palliatus] |
|  |  | 80 | NP_443167.4 | 7-methylguanosine phosphate-specific 5'-nucleotidase [Homo sapiens] |
| 145 | Query=c67390/f1p0/912 | 91 | AAA36435.1 | phosphofructokinase, partial [Homo sapiens] |
| 146 | Query=c67599/f4p1/1310 | 97 | XP_008961373.1 | PREDICTED: UDP-glucose 4-epimerase isoform X2 [Pan paniscus] |
|  |  | 89 | NP_001008217.1 | UDP-glucose 4-epimerase [Homo sapiens] |
| 147 | Query=c68304/f1p0/1106 | 91 | XP_011543554.1 | PREDICTED: probable dolichyl pyrophosphate Glc1Man9GlcNAc2 alpha-1,3-glucosyltransferase isoform X3 [Homo sapiens] |
| 148 | Query=c68366/f1p4/1350 | 90 | XP_012365838.1 | PREDICTED: kunitz-type protease inhibitor 2 isoform X3 [Nomascus leucogenys] |
|  |  | 84 | NP_066925.1 | kunitz-type protease inhibitor 2 isoform a precursor [Homo sapiens] |
| 149 | Query=c68865/f1p2/1634 | 96 | NP_001161819.1 | probable 28S rRNA (cytosine-C(5))-methyltransferase isoform 3 [Homo sapiens] |
| 150 | Query=c68914/f1p0/1161 | 84 | AAA61169.1 | thyroid hormone binding protein precursor [Homo sapiens] |
| 151 | Query=c69259/f1p1/2198 | 94 | XP_016861362.1 | PREDICTED: 5-aminolevulinate synthase, nonspecific, mitochondrial isoform X3 [Homo sapiens] |
| 152 | Query=c69459/f1p1/515 | 93 | NP_005166.1 | ATP synthase F(0) complex subunit C1, mitochondrial precursor [Homo sapiens] |
| 153 | Query=c69609/f1p0/1249 | 96 | NP_997078.1 | disintegrin and metalloproteinase domain-containing protein 15 isoform 4 preproprotein [Homo sapiens] |
| 154 | Query=c69895/f1p0/1248 | 83 | NP_056494.1 | probable tRNA pseudouridine synthase 2 isoform 1 [Homo sapiens] |
| 155 | Query=c70185/f1p1/1544 | 93 | NP_001159631.1 | NADH dehydrogenase [ubiquinone] iron-sulfur protein 2, mitochondrial isoform 2 precursor [Homo sapiens] |
| 156 | Query=c70504/f1p3/1246 | 87 | NP_006294.2 | aminoacyl tRNA synthase complex-interacting multifunctional protein 2 isoform a [Homo sapiens] |
| 157 | Query=c70852/f2p13/1398 | 93 | NP_055629.1 | 26S proteasome non-ATPase regulatory subunit 6 isoform 2 [Homo sapiens] |
| 158 | Query=c7232/f1p2/884 | 89 | NP_001129674.1 | isochorismatase domain-containing protein 2 isoform 3 [Homo sapiens] |
| 159 | Query=c75778/f1p2/1125 | 87 | NP_775956.1 | E3 SUMO-protein ligase NSE2 [Homo sapiens] |
| 160 | Query=c7583/f1p3/2067 | 84 | NP_004554.3 | phosphoenolpyruvate carboxykinase [GTP], mitochondrial isoform 1 precursor [Homo sapiens] |
| 161 | Query=c76047/f1p0/1984 | 81 | XP_006719021.1 | PREDICTED: chromodomain-helicase-DNA-binding protein 4 isoform X1 [Homo sapiens] |
| 162 | Query=c76569/f1p1/2069 | 87 | EMP23777.1 | Phosphoribosyl pyrophosphate synthase-associated protein 1 [Chelonia mydas] |
| 163 | Query=c76625/f1p2/1241 | 93 | NP_291028.3 | TP53-regulating kinase [Homo sapiens] |
| 164 | Query=c76731/f1p0/1196 | 97 | XP_009232493.1 | PREDICTED: LOW QUALITY PROTEIN: serine/threonine-protein kinase Chk2 [Pongo abelii] |
|  |  | 92 | AAS58464.1 | protein kinase Chk2 transcript variant del2-3 [Homo sapiens] |
| 165 | Query=c77384/f1p0/942 | 93 | XP_010814296.1 | PREDICTED: arachidonate 12-lipoxygenase, 12R-type isoform X1 [Bos taurus] |
|  |  | 75 | NP_001130.1 | arachidonate 12-lipoxygenase, 12R-type [Homo sapiens] |
| 166 | Query=c78105/f1p0/883 | 95 | NP_001910.2 | enoyl-CoA delta isomerase 1, mitochondrial isoform 1 precursor [Homo sapiens] |
| 167 | Query=c78505/f1p3/2526 | 90 | NP_001245367.1 | threonine--tRNA ligase, cytoplasmic isoform 2 [Homo sapiens] |
| 168 | Query=c78677/f1p2/1542 | 94 | XP_015001672.1 | PREDICTED: repressor of RNA polymerase III transcription MAF1 homolog isoform X1 [Macaca mulatta] |
|  |  | 94 | XP_009197183.1 | PREDICTED: repressor of RNA polymerase III transcription MAF1 homolog isoform X1 [Papio anubis] |
| 169 | Query=c78680/f1p0/1186 | 74 | XP_012354678.1 | PREDICTED: LOW QUALITY PROTEIN: isoaspartyl peptidase/L-asparaginase [Nomascus leucogenys] |
|  |  | 73 | NP_079356.3 | isoaspartyl peptidase/L-asparaginase [Homo sapiens] |
| 170 | Query=c78963/f1p2/1685 | 79 | NP_066981.2 | tribbles homolog 3 isoform 1 [Homo sapiens] |
| 171 | Query=c79220/f1p2/1939 | 95 | NP_006627.2 | bifunctional methylenetetrahydrofolate dehydrogenase/cyclohydrolase, mitochondrial precursor [Homo sapiens] |
| 172 | Query=c79242/f1p5/1204 | 85 | NP_001527.3 | protein arginine N-methyltransferase 1 isoform 1 [Homo sapiens] |
| 173 | Query=c79256/f1p10/1228 | 98 | XP_014384124.1 | PREDICTED: 26S proteasome non-ATPase regulatory subunit 4 isoform X4 [Myotis brandtii] |
|  |  | 93 | NP_002801.1 | 26S proteasome non-ATPase regulatory subunit 4 isoform 2 [Homo sapiens] |
| 174 | Query=c80468/f1p2/1835 | 86 | NP_002622.2 | 6-phosphogluconate dehydrogenase, decarboxylating isoform 1 [Homo sapiens] |
| 175 | Query=c81306/f1p5/961 | 82 | NP_001304007.1 | mediator of RNA polymerase II transcription subunit 19 isoform 1 [Homo sapiens] |
| 176 | Query=c86600/f1p0/684 | 73 | NP_054761.1 | anaphase-promoting complex subunit 15 isoform b [Homo sapiens] |
| 177 | Query=c87887/f1p4/1307 | 95 | NP_057042.2 | diphthine methyl ester synthase isoform a [Homo sapiens] |
| 178 | Query=c88922/f1p1/1847 | 77 | XP_011813447.1 | PREDICTED: phospholipid scramblase 1 isoform X1 [Colobus angolensis palliatus] |
|  |  | 62 | NP_066928.1 | phospholipid scramblase 1 [Homo sapiens] |
| 179 | Query=c89347/f1p0/941 | 84 | EAW50982.1 | succinate dehydrogenase complex, subunit A, flavoprotein (Fp), isoform CRA_a [Homo sapiens] |
| 180 | Query=c89471/f1p2/1298 | 79 | NP_002800.2 | 26S proteasome non-ATPase regulatory subunit 3 [Homo sapiens] |
| 181 | Query=c89745/f1p0/887 | 80 | XP_011735684.1 | PREDICTED: sentrin-specific protease 6 isoform X1 [Macaca nemestrina] |
|  |  | 79 | NP_001291721.1 | sentrin-specific protease 6 isoform 3 [Homo sapiens] |
| 182 | Query=c8990/f1p3/1532 | 95 | XP_011785139.1 | PREDICTED: 7-methylguanosine phosphate-specific 5'-nucleotidase isoform X1 [Colobus angolensis palliatus] |
|  |  | 89 | AAH14132.2 | 5'-nucleotidase, cytosolic III-like [Homo sapiens] |
| 183 | Query=c91368/f1p1/1340 | 95 | NP_001216.1 | caspase-4 isoform alpha precursor [Homo sapiens] |
| 184 | Query=c91475/f1p3/862 | 100 | XP_004060478.1 | PREDICTED: cytochrome b-c1 complex subunit Rieske, mitochondrial-like isoform 1 [Gorilla gorilla gorilla] |
|  |  | 83 | NP_005994.2 | cytochrome b-c1 complex subunit Rieske, mitochondrial [Homo sapiens] |
| 185 | Query=c91605/f1p12/912 | 83 | NP_000933.1 | peptidyl-prolyl cis-trans isomerase B precursor [Homo sapiens] |
| 186 | Query=c9368/f1p1/2914 | 85 | XP_006719669.1 | PREDICTED: N-alpha-acetyltransferase 25, NatB auxiliary subunit isoform X1 [Homo sapiens] |
| 187 | Query=c97718/f1p2/1457 | 93 | NP_004631.1 | spliceosome RNA helicase DDX39B [Homo sapiens] |
| 188 | Query=c97742/f1p1/1581 | 91 | NP_071439.3 | tRNA-dihydrouridine(16/17) synthase [NAD(P)(+)]-like [Homo sapiens] |
| 189 | Query=c98313/f1p0/1483 | 88 | XP_005245271.1 | PREDICTED: nitrilase homolog 1 isoform X2 [Homo sapiens] |
| 190 | Query=c98955/f1p0/785 | 70 | NP_001035218.1 | serine protease inhibitor Kazal-type 13 precursor [Homo sapiens] |
| 191 | Query=c99410/f1p7/1325 | 92 | NP_001243330.1 | elongation of very long chain fatty acids protein 1 isoform 2 [Homo sapiens] |
| 192 | Query=c99717/f1p0/1457 | 89 | NP_000146.2 | galactose-1-phosphate uridylyltransferase isoform 1 [Homo sapiens] |

**The detail of a hormone metabolic enzyme in A549 cells：**

**Table S8. The mutation of estradiol 17*β-*dehydrogenase**

| cDNA | Identity% | Sequence ID(Ref.) | Predicited Protein |
| --- | --- | --- | --- |
| Query= c22055/f2p2/2293 | 79 | XP_011523034.1 | Estradiol 17*β*-dehydrogenase 1 isoform X8 [Homo sapiens] |

**The amino acid sequence of estradiol 17*β-*dehydrogenase was compared with the NCBI database:**

>Query=c22055/f2p2/2293
MARTVVLITGCSSGIGLHLAVRLASDPSQSFKGIDRQGQGGREGRSPWRLEGKSDLPPLPKPPVYATLRDLKTQGRLWEAARALACPPGSLETLQLDVRDSKSVAAARERVTEGRVDVLGEPPGSIWAPRSLLRPALKPTCSQAQGARGDRPC*g*c*gglvgplVSAVCNaglgllgplealgEDAVASVLDVNVVGTVRMLQAFLPDMKRRGSGRVLVTGSVGGLMG

PREDICTED: estradiol 17-beta-dehydrogenase 1 isoform X8 [Homo sapiens]

Sequence ID: [XP_011523034.1](https://www.ncbi.nlm.nih.gov/protein/767994581?report=genbank&log$=protalign&blast_rank=1&RID=YPCWEB5J01R)Length: 180Number of Matches: 1

Related Information

[Gene](https://www.ncbi.nlm.nih.gov/gene?term=767994581%5bPUID%5d&RID=YPCWEB5J01R&log$=genealign&blast_rank=1)-associated gene details

Range 1: 1 to 179[GenPept](https://www.ncbi.nlm.nih.gov/protein/767994581?report=genbank&log$=protalign&blast_rank=1&RID=YPCWEB5J01R&from=1&to=179)[Graphics](https://www.ncbi.nlm.nih.gov/protein/767994581?report=graph&rid=YPCWEB5J01R%5b767994581%5d&tracks=%5bkey:sequence_track,name:Sequence,display_name:Sequence,id:STD1,category:Sequence,annots:Sequence,ShowLabel:true%5d%5bkey:gene_model_track,CDSProductFeats:false%5d%5bkey:alignment_track,name:other%20alignments,annots:NG%20Alignments|Refseq%20Alignments|Gnomon%20Alignments|Unnamed,shown:false%5d&v=0:187&appname=ncbiblast&link_loc=fromHSP)Next MatchPrevious Match

**Table S9. Alignment statistics for match #1**

| **Score** | **Expect** | **Method** | **Identities** | **Positives** | **Gaps** |
| --- | --- | --- | --- | --- | --- |
| 322 bits(826) | 3e-110 | Compositional matrix adjust. | 178/228(78%) | 178/228(78%) | 49/228(21%) |

Query 1 MARTVVLITGCSSGIGLHLAVRLASDPSQSFKGIDRQGQGGREGRSPWRLEGKSDLPPLP 60 MARTVVLITGCSSGIGLHLAVRLASDPSQSFKGIDRQGQGGREGRSPWR EGKSDLPPLP

Sbjct 1 MARTVVLITGCSSGIGLHLAVRLASDPSQSFKGIDRQGQGGREGRSPWRPEGKSDLPPLP 60

Query 61 KPPVYATLRDLKTQGRLWEAARALACPPGSLETLQLDVRDSKSVAAARERVTEGRVDVLG 120 KPPVYATLRDLKTQGRLWEAARALACPPGSLETLQLDVRDSKSVAAARERVTEGRVDVL

Sbjct 61 KPPVYATLRDLKTQGRLWEAARALACPPGSLETLQLDVRDSKSVAAARERVTEGRVDVL- 119

Query 121 EPPGSIWAPRSLLRPALKPTCSQAQGARGDRPC*G*C*GGLVGPLVSAVCNAGLGLLGPL 180 VCNAGLGLLGPL

Sbjct 120 ------------------------------------------------VCNAGLGLLGPL 131

Query 181 EALGEDAVASVLDVNVVGTVRMLQAFLPDMKRRGSGRVLVTGSVGGLM 228 EALGEDAVASVLDVNVVGTVRMLQAFLPDMKRRGSGRVLVTGSVGGLM

Sbjct 132 EALGEDAVASVLDVNVVGTVRMLQAFLPDMKRRGSGRVLVTGSVGGLM 179

**Table S10. Mutant enzymes in HepG2 cells**

| **No.** | **cDNA** | **Identities**  **(%)** | **Sequence ID** | **Proteins** |
| --- | --- | --- | --- | --- |
| 1 | Query=c102202/f2p1/1262 | 82 | [gb\|EAX01712.1\|](http://www.ncbi.nlm.nih.gov/protein/119622117?report=genbank&log$=protalign&blast_rank=2&RID=JKXW81WV014) | enolase superfamily member 1,isoform CRA_b [Homo sapiens] |
| 2 | Query=c102511/f1p1/878 | 83 | [gb\|AAH06199.3\|](http://www.ncbi.nlm.nih.gov/protein/37588925?report=genbank&log$=protalign&blast_rank=4&RID=JKZGR23M014) | LAP3 protein, partial [Homo sapiens] |
| 3 | Query=c102764/f1p0/2522 | 80 | [gb\|AAH35132.1\|](http://www.ncbi.nlm.nih.gov/protein/23243453?report=genbank&log$=protalign&blast_rank=1&RID=JM0TS8V601R) | Threonine synthase-like 1 (S. cerevisiae) [Homo sapiens] |
| 4 | Query=c102989/f1p6/3551 | 98 | ref\|NP_115587.6\| | lysyl oxidase homolog 4 precursor [Homo sapiens] |
| 5 | Query=c103270/f1p2/1948 | 91 | ref\|NP_006750.3\| | UTP--glucose-1-phosphate uridylyltransferase isoform a [Homo sapiens] |
| 6 | Query=c103708/f1p3/1807 | 99 | ref\|XP_016800098.1\| | PREDICTED: NF-kappa-B essential modulator isoform X4 [Pan troglodytes] |
|  |  | 88 | ref\|NP_003630.1\| | NF-kappa-B essential modulator isoform a [Homo sapiens] |
| 7 | Query=c104045/f1p1/2805 | 92 | [gb\|AAH92466.1\|](http://www.ncbi.nlm.nih.gov/protein/62201619?report=genbank&log$=protalign&blast_rank=3&RID=JMEKCFWB015) | TTC3 protein,partial [Homo sapiens] |
| 8 | Query=c104189/f1p1/1604 | 91 | [ref\|XP_003279945.1\|](http://www.ncbi.nlm.nih.gov/protein/332261781?report=genbank&log$=protalign&blast_rank=1&RID=JMF7AH3M014) | dolichyl-diphosphooligosaccharide--protein glycosyltransferase subunit 1 [Nomascus leucogenys] |
|  |  | 90 | [ref\|NP_002941.1\|](http://www.ncbi.nlm.nih.gov/protein/4506675?report=genbank&log$=protalign&blast_rank=10&RID=JMF7AH3M014) | dolichyl-diphosphooligosaccharide--protein glycosyltransferase subunit 1 precursor [Homo sapiens] |
| 9 | Query=c104410/f1p5/1232 | 92 | gb\|AAX41013.1 | PTEN induced putative kinase 1 [synthetic construct] |
|  |  | 92 | ref\|NP_115785.1 | serine/threonine-protein kinase PINK1,mitochondrial precursor [Homo sapiens] |
| 10 | Query=c105341/f1p0/21 | 85 | [gb\|EAW65377.1\|](http://www.ncbi.nlm.nih.gov/protein/119585781?report=genbank&log$=protalign&blast_rank=1&RID=JMJGE14M01R) | acyl-Coenzyme A oxidase 2,branched chain,isoform CRA_c [Homo sapiens] |
| 11 | Query=c105675/f1p4/2813 | 84 | [ref\|NP_079103.2\|](http://www.ncbi.nlm.nih.gov/protein/217272877?report=genbank&log$=protalign&blast_rank=1&RID=JMKACSRP015) | histone deacetylase 11 isoform 1 [Homo sapiens] |
| 10 | Query=c105905/f1p0/1495 | 98 | [ref\|NP_570844.1\|](http://www.ncbi.nlm.nih.gov/protein/18860831?report=genbank&log$=protalign&blast_rank=1&RID=JMM1FFXM014) | dynamin-like 120 kDa protein,mitochondrial isoform 2 [Homo sapiens] |
| 13 | Query=c105947/f1p0/1336 | 90 | [ref\|XP_011512535.1\|](http://www.ncbi.nlm.nih.gov/protein/767939082?report=genbank&log$=protalign&blast_rank=1&RID=JMMNFV06015) | adenylyl cyclase-associated protein 2 isoform X1 [Homo sapiens] |
| 14 | Query=c106006/f1p1/1431 | 87 | [ref\|XP_011535277.1\|](http://www.ncbi.nlm.nih.gov/protein/767981059?report=genbank&log$=protalign&blast_rank=1&RID=JMNWXT5H014) | epimerase family protein SDR39U1 isoform X3 [Homo sapiens] |
| 15 | Query=c106145/f1p0/91 | 81 | gb\|AAD29855.1\|AF083068_1 | NAD+ADP-ribosyltransferase 3 [Homo sapiens] |
| 16 | Query=c106191/f1p6/107 | 85 | [ref\|XP_007122332.1\|](http://www.ncbi.nlm.nih.gov/protein/593767694?report=genbank&log$=protalign&blast_rank=1&RID=JMPS3PPE014) | peptidyl-prolyl cis-trans isomerase G-like isoform X1 [Physeter catodon] |
|  |  | 85 | [gb\|EAX11266.1\|](http://www.ncbi.nlm.nih.gov/protein/119631671?report=genbank&log$=protalign&blast_rank=6&RID=JMPS3PPE014) | peptidylprolyl isomerase G (cyclophilin G),isoform CRA_a,partial [Homo sapiens] |
| 17 | Query=c106452/f1p1/2210 | 95 | gb\|ABA10576.1\| | Dha kinase/FMN cyclase [Homo sapiens] |
| 19 | Query=c106889/f1p2/1763 | 83 | NP_002620.1 | phosphoglycerate mutase 1 isoform 1 [Homo sapiens] |
| 20 | Query=c107162/f1p0/3314 | 83 | NP_001035918.1 | serine/threonine-protein kinase 26 isoform 2 [Homo sapiens] |
| 21 | Query=c107201/f1p6/2342 | 98 | EAW50264.1 | hCG1994151， isoform CRA_e [Homo sapiens] |
| 22 | Query=c107718/f1p3/995 | 91 | ref\|XP_011527536.1\| | PREDICTED: inosine triphosphate pyrophosphatase isoform X3 [Homo sapiens] |
| 23 | Query=c107987/f1p11/1719 | 100 | ref\|XP_016780893.1\| | PREDICTED: LIM domain only protein 7 isoform X22 [Pan troglodytes] |
|  |  | 80 | ref\|NP_005993.1\| | ubiquitin carboxyl-terminal hydrolase isozyme L3 isoform 2 [Homo sapiens] |
| 24 | Query=c108086/f1p1/2363 | 93 | AAD27766.1 | putative ATP-dependent RNA helicase ROK1 [Homo sapiens] |
| 25 | Query=c108109/f1p10/2280 | 89 | gb\|EAW77886.1\| | pyruvate kinase， muscle， isoform CRA_c [Homo sapiens] |
| 26 | Query=c109027/f1p0/2291 | 76 | XP_012657798.1 | lipid phosphate phosphatase-related protein type 1 isoform X2 [Otolemurgarnettii] |
|  |  | 76 | EAW58941.1 | plasticity related gene 3， isoform CRA_b [Homo sapiens] |
| 27 | Query=c11195/f1p9/3413 | 96 | NP_060105.3 | histone-lysine N-methyltransferase KMT5B isoform 1 [Homo sapiens] |
| 28 | Query=c11216/f4p54/985 | 95 | XP_003278677.1 | V-type proton ATPase 21 kDa proteolipid subunit isoform X2 [Nomascusleucogenys] |
|  |  | 92 | NP_004038.1 | V-type proton ATPase 21 kDa proteolipid subunit isoform 1 [Homo sapiens] |
| 29 | Query=c11379/f1p4/3029 | 94 | AKI72625.1 | atypical kinase ADCK3,partial [synthetic construct] |
|  |  | 94 | XP_005273258.1 | chaperone activity of bc1 complex-like,mitochondrial isoform X1 [Homo sapiens] |
| 30 | Query=c11386/f1p2/1427 | 79 | XP_005245293.1 | protein RRNAD1 isoform X4 [Homo sapiens] |
| 31 | Query=c11464/f1p1/790 | 79 | XP_004028900.1 | rho-associated protein kinase 2 [Gorilla gorilla gorilla] |
|  |  | 79 | NP_001308572.1 | rho-associated protein kinase 2 isoform 2 [Homo sapiens] |
| 32 | Query=c188636/f11p81/1190 | 97 | AFF79606.1 | NADH dehydrogenase subunit 4 (mitochondrion) [Homo sapiens] |
| 33 | Query=c121754/f2p49/1351 | 96 | dbj\|BAE09988.1 | cytochrome b[Homo sapiens] |
| 34 | Query=c122025/f1p11/1178 | 99 | [ref\|XP_010381777.1\|](https://www.ncbi.nlm.nih.gov/protein/724923581?report=genbank&log$=protalign&blast_rank=1&RID=UNP6TBMW01R) | PREDICTED: uroporphyrinogen decarboxylase isoform X2 [Rhinopithecus roxellana] |
|  |  | 94 | [gb\|AAC50482.1\|](https://www.ncbi.nlm.nih.gov/protein/1322019?report=genbank&log$=protalign&blast_rank=4&RID=UNP6TBMW01R) | uroporphyrinogen decarboxylase [Homo sapiens] |
| 35 | Query=c122348/f1p6/1036 | 91 | gb\|AAC41939.1 | cytoplasmic antiproteinase 2[Homo sapiens] |
| 36 | Query=c123167/f1p4/1617 | 98 | ref\|NP_006102.2 | 3-ketoacyl-CoA thiolase[Homo sapiens] |
| 37 | Query=c123212/f1p1/2815 | 79 | NP_001257356.1 | 26S proteasome non-ATPase regulatory subunit 5 isoform 2[Homo sapiens] |
| 38 | Query=c123812/f1p2/1219 | 90 | ref\|NP_005362.3 | tRNA (guanine-N(7)-)-methyltransferase isoform a[Homo sapiens] |
| 39 | Query=c123946/f1p1/2467 | 76 | gb\|AAH44575.1 | Tryptophanyl tRNA synthetase 2[Homo sapiens] |
| 40 | Query=c124401/f1p2/2141 | 98 | [ref\|XP_016878169.1\|](https://www.ncbi.nlm.nih.gov/protein/1034592178?report=genbank&log$=protalign&blast_rank=5&RID=17D4ZT80014) | PREDICTED: signal peptide peptidase-like 2A isoform X2 [Homo sapiens] |
| 41 | Query=c124416/f1p1/1614 | 87 | ref\|NP_006801.1 | protein disulfide-isomerase A5 precursor[Homo sapiens] |
| 42 | Query=c124468/f2p5/1202 | 95 | gb\|AIH15150.1 | cytochrome c oxidase subunit I[Homo sapiens] |
| 43 | Query=c124474/f1p5/1115 | 84 | ref\|NP_004308.2 | ATPase ASNA1[Homo sapiens] |
| 44 | Query=c124621/f1p3/2151 | 93 | ref\|NP_005759.4 | lysophospholipid acyltransferase 5[Homo sapiens] |
| 45 | Query=c124700/f1p0/1521 | 99 | [ref\|XP_016787435.1\|](https://www.ncbi.nlm.nih.gov/protein/1034121138?report=genbank&log$=protalign&blast_rank=1&RID=UNU31M6S014) | PREDICTED: probable ATP-dependent RNA helicase DDX52 isoform X3 [Pan troglodytes] |
|  |  | 91 | [ref\|NP_008941.3\|](https://www.ncbi.nlm.nih.gov/protein/612407837?report=genbank&log$=protalign&blast_rank=9&RID=UNU31M6S014) | probable ATP-dependent RNA helicase DDX52 isoform 1 [Homo sapiens] |
| 46 | Query=c125084/f1p1/1837 | 93 | dbj\|BAG70035.1\| | mitogen-activated protein kinase 13 [Homo sapiens] |
| 47 | Query=c125166/f2p7/1801 | 87 | ref\|NP_001035937.1 | monoacylglycerol lipase ABHD12 isoform a[Homo sapiens] |
| 48 | Query=c125616/f2p0/2847 | 93 | ref\|NP_060327.3 | protein phosphatase Slingshot homolog 3[Homo sapiens] |
| 49 | Query=c125998/f2p2/1909 | 96 | ref\|NP_001229815.1\| | aromatic-L-amino-acid decarboxylase isoform 2[Homo sapiens] |
| 50 | Query=c126321/f1p6/1081 | 95 | ref\|NP_001975.1 | S-formylglutathione hydrolase[Homo sapiens] |
| 51 | Query=c127295/f1p2/2797 | 84 | [gb\|AAC41949.1\|](https://www.ncbi.nlm.nih.gov/protein/1220313?report=genbank&log$=protalign&blast_rank=8&RID=UNYMXPXW016) | interleukin-1 receptor-associated kinase [Homo sapiens] |
| 52 | Query=c127486/f1p10/1120 | 83 | ref\|NP_001001973.1 | ATP synthase subunit gamma， mitochondrial isoform L (liver) precursor[Homo sapiens] |
| 53 | Query=c127533/f1p3/1919 | 98 | dbj\|BAD92552.1\| | serine/threonine kinase 4 variant, partial [Homo sapiens] |
| 54 | Query=c127640/f1p10/4230 | 84 | gb\|AAH16657.1 | Procollagen-lysine 1,2-oxoglutarate 5-dioxygenase 1[Homo sapiens] |
| 55 | Query=c127644/f1p3/4219 | 93 | ref\|XP_006712164.1 | CAD protein isoform X4[Homo sapiens] |
| 56 | Query=c127747/f1p2/1603 | 84 | dbj\|BAA33714.1 | NIK[Homo sapiens] |
| 57 | Query=c127828/f1p1/1012 | 87 | ref\|NP_550438.1 | deoxyguanosine kinase， mitochondrial precursor isoform a[Homo sapiens] |
| 58 | Query=c127883/f1p2/2061 | 85 | ref\|NP_001164554.1 | sphingomyelin phosphodiesterase 4 isoform 3  [Homo sapiens] |
| 59 | Query=c127892/f1p2/1463 | 86 | ref\|NP_001138497.1\| | ER degradation-enhancing alpha-mannosidase-like protein 2 isoform 2 precursor [Homo sapiens] |
| 60 | Query=c127931/f1p8/4429 | 78 | ref\|NP_000268.1\| | phenylalanine-4-hydroxylase [Homo sapiens] |
| 61 | Query=c128332/f1p10/2831 | 92 | ref\|XP_011528595.1\| | PREDICTED: ran GTPase-activating protein 1 isoform X4 [Homo sapiens] |
| 62 | Query=c128516/f1p1/1269 | 84 | gb\| ABD77315.1 | succinate dehydrogenase complex subunit A,partial [Homo sapiens] |
| 63 | Query=c128687/f1p2/1372 | 99 | [ref\|XP_008966148.1\|](https://www.ncbi.nlm.nih.gov/protein/675730959?report=genbank&log$=protalign&blast_rank=1&RID=URMFB248014) | PREDICTED: enoyl-CoA hydratase domain-  containing protein 2,mitochondrial isoform X6 [Pan paniscus] |
|  |  | 91 | [ref\|NP_060751.2\|](https://www.ncbi.nlm.nih.gov/protein/150378541?report=genbank&log$=protalign&blast_rank=4&RID=URMFB248014) | enoyl-CoA hydratase domain-containing protein 2,mitochondrial isoform 2 [Homo sapiens] |
| 64 | Query=c128736/f1p4/1509 | 93 | gb\|AAH21707.1 | Tubulin tyrosine ligase-like family,member 4 [Homo sapiens] |
| 65 | Query=c128968/f1p3/3146 | 87 | gb\|AAW65983.1\| | HECTD1 [Homo sapiens] |
| 66 | Query=c142519/f2p2/1552 | 98 | ref\|XP_009001624.1 | alpha-N-acetyl-neuraminyl-2,3-beta-galactosyl-1,3-N-acetyl-galactosaminide alpha-2,6-sialyltransferase isoform X2 [Callithrix jacchus] |
|  |  | 88 | ref\|NP_778204.1 | alpha-N-acetyl-neuraminyl-2,3-beta-galactosyl-1,3-N-acetyl-galactosaminide alpha-2,6-sialyltransferase isoform a [Homo sapiens] |
| 67 | Query=c14254/f1p4/1758 | 89 | ref\|NP_060666.1 | trimethyllysine dioxygenase,mitochondrial isoform 1 precursor [Homo sapiens] |
| 68 | Query=c142589/f1p1/1778 | 99 | ref\|XP_012305912.1 | glutaminase liver isoform,mitochondrial isoform X1 [Aotus nancymaae] |
|  |  | 96 | ref\|NP_037399.2 | glutaminase liver isoform,mitochondrial isoform 1 precursor [Homo sapiens] |
| 69 | Query=c143242/f1p2/1908 | 96 | ref\|NP_055660.1 | polyphosphoinositide phosphatase [Homo sapiens] |
| 70 | Query=c143399/f2p0/1134 | 97 | ref\|XP_005273594.1 | DNA polymerase beta isoform X3 [Homo sapiens] |
| 71 | Query=c143596/f1p0/991 | 100 | ref\|XP_010378061.1 | glutaminase kidney isoform,mitochondrial isoform X3 [Rhinopithecus roxellana] |
|  |  | 95 | ref\|XP_006712498.1 | glutaminase kidney isoform,mitochondrial isoform X1 [Homo sapiens] |
| 72 | Query=c143870/f1p1/1918 | 79 | gb\|AAA35650.1\| | carboxylesterase, partial [Homo sapiens] |
| 73 | Query=c144026/f1p2/2049 | 98 | gb\|ALQ33737.1 | protein kinase C substrate 80K-H isoform 2, partial [Homo sapiens] |
| 74 | Query=c144268/f1p0/1485 | 93 | ref\|XP_011528042.1 | E3 ubiquitin-protein ligase TTC3 isoform X5 [Homo sapiens] |
| 75 | Query=c144321/f1p0/1388 | 91 | XP_016860862.1 | PREDICTED: mitogen-activated protein kinase kinase kinase kinase 4 isoform X42 [Homo sapiens] |
| 76 | Query=c14480/f1p2/1633 | 84 | gb\|ABD77215.1 | isocitrate dehydrogenase 1 [Homo sapiens] |
| 77 | Query=c144920/f1p2/1787 | 84 | ref\|XP_011543978.1 | monofunctional C1-tetrahydrofolate synthase,mitochondrial-like isoform X1 [Homo sapiens] |
| 78 | Query=c144984/f1p0/2428 | 84 | ref\|NP_001278388.1 | TBC1 domain family member 3F-like [Homo sapiens] |
| 79 | Query=c144995/f1p2/1990 | 94 | ref\|XP_009191055.1 | Ferrochelatase,mitochondrial isoform X3 [Papio anubis] |
|  |  | 93 | dbj\|BAA00628.1 | ferrochelatase precursor [Homo sapiens] |
| 80 | Query=c145114/f1p1/2285 | 95 | ref\|NP_001883.4 | casein kinase I isoform alpha isoform 2 [Homo sapiens] |
| 81 | Query=c145119/f1p1/1077 | 97 | ref\|XP_016868281.1 | serine/threonine-protein kinase 17A isoform X1 [Homo sapiens] |
| 82 | Query=c145127/f1p3/2105 | 85 | ref\|XP_016311365.1 | chromodomain-helicase-DNA-binding protein 4-like [Sinocyclocheilus anshuiensis] |
|  |  | 72 | ref\|NP_001284482.1 | chromodomain-helicase-DNA-binding protein 4 isoform 2 [Homo sapiens] |
| 83 | Query=c145153/f1p3/3613 | 95 | ref\|NP_001287993.1 | RNA polymerase I-specific transcription initiation factor RRN3 isoform 2 [Homo sapiens] |
| 84 | Query=c145446/f1p3/2473 | 89 | [gb\|EAW71677.1](http://www.ncbi.nlm.nih.gov/protein/119592083?report=genbank&log$=protalign&blast_rank=1&RID=K5VKK1KH01R) | exosome component 10， isoform CRA_a,partial [Homo sapiens] |
| 85 | Query=c145484/f1p1/1832 | 82 | [gb\|EAW89696.1](http://www.ncbi.nlm.nih.gov/protein/119610102?report=genbank&log$=protalign&blast_rank=2&RID=K5XCJPJ701R) | procollagen-proline,2-oxoglutarate 4-dioxygenase (proline 4-hydroxylase),beta polypeptide,isoform CRA_d [Homo sapiens] |
| 86 | Query=c145490/f7p81/1613 | 84 | gb\|AAP48010.1\| | NADH dehydrogenase subunit 1 (mitochondrion) [Homo sapiens] |
| 87 | Query=c145545/f1p1/2625 | 94 | [pdb\|2C35\|A](http://www.ncbi.nlm.nih.gov/protein/83754528?report=genbank&log$=protalign&blast_rank=1&RID=K607J6SJ015) | Chain A,Subunits Rpb4 And Rpb7 Of Human Rna Polymerase Ii |
| 88 | Query=c145775/f1p2/1311 | 81 | ref\|NP_004308.2 | ATPase ASNA1 [Homo sapiens] |
| 89 | Query=c146093/f1p3/1655 | 84 | ref\|NP_001161820.1 | probable 28S rRNA (cytosine-C(5))-methyltransferase isoform 4 [Homo sapiens] |
| 90 | Query=c146220/f1p2/3536 | 93 | ref\|XP_011539823.1 | nardilysin isoform X1 [Homo sapiens] |
| 91 | Query=c146467/f1p0/1660 | 90 | ref\|NP_003792.1 | glycosylphosphatidylinositol anchor attachment 1 protein [Homo sapiens] |
| 92 | Query=c146686/f1p0/1065 | 53 | ref\|XP_008836154.1 | O-acetyl-ADP-ribose deacetylase 1 [Nannospalax galili] |
|  |  | 53 | ref\|NP_659500.1 | O-acetyl-ADP-ribose deacetylase 1 [Homo sapiens] |
| 93 | Query=c147031/f1p3/3751 | 82 | ref\|XP_012914357.1 | ADP-ribosylation factor-like protein 5B isoform X2 [Mustela putorius furo] |
|  |  | 82 | ref\|NP_848930.1 | ADP-ribosylation factor-like protein 5B [Homo sapiens] |
| 94 | Query=c147091/f1p0/1211 | 75 | ref\|NP_001275765.1 | serine/threonine-protein kinase VRK2 isoform 4 [Homo sapiens] |
| 95 | Query=c14710/f1p5/1736 | 73 | [gb\|AAA83932.1\|](http://www.ncbi.nlm.nih.gov/protein/187029?report=genbank&log$=protalign&blast_rank=3&RID=KBY0R8U0015) | Carboxylesterase,partial [Homo sapiens] |
| 96 | Query=c147575/f1p3/2095 | 87 | [ref\|NP_004981.2\|](http://www.ncbi.nlm.nih.gov/protein/14043022?report=genbank&log$=protalign&blast_rank=1&RID=KC0281KE014) | methionine--tRNA ligase, cytoplasmic [Homo sapiens] |
| 97 | Query=c147696/f1p1/1072 | 94 | [gb\|AAH46205.1\|](http://www.ncbi.nlm.nih.gov/protein/28374296?report=genbank&log$=protalign&blast_rank=2&RID=KC11MKEP015) | USP9X protein,partial [Homo sapiens] |
| 98 | Query=c147815/f1p0/2222 | 89 | [ref\|NP_001311381.1\|](http://www.ncbi.nlm.nih.gov/protein/1025812241?report=genbank&log$=protalign&blast_rank=1&RID=KC2R2N2Z01R) | tRNA-specific adenosine deaminase 1 isoform e [Homo sapiens] |
| 99 | Query=c147822/f1p5/2230 | 88 | ref\|NP_001308086.1 | methyltransferase-like protein 8 isoform 2 precursor [Homo sapiens] |
| 100 | Query=c147952/f1p0/2249 | 95 | ref\|XP_005273204.1 | serine/threonine-protein kinase Nek2 isoform X1 [Homo sapiens] |
| 101 | Query=c148191/f1p2/2187 | 91 | [ref\|NP_001229921.1\|](http://www.ncbi.nlm.nih.gov/protein/339715201?report=genbank&log$=protalign&blast_rank=4&RID=KC4RBPVB01R) | poly(A)-specific ribonuclease PARN isoform 3 [Homo sapiens] |
| 102 | Query=c148581/f1p0/1238 | 93 | [ref\|XP_008837881.1](http://www.ncbi.nlm.nih.gov/protein/532114740?report=genbank&log$=protalign&blast_rank=1&RID=KC6KWTHC01R) | 2-oxoglutarate and iron-dependent oxygenase domain-containing protein 3 isoform X2 [Nannospalax galili] |
|  |  | 75 | [ref\|NP_078924.1\|](http://www.ncbi.nlm.nih.gov/protein/13375891?report=genbank&log$=protalign&blast_rank=6&RID=KC6KWTHC01R) | 2-oxoglutarate and iron-dependent oxygenase domain-containing protein 3 isoform 1 [Homo sapiens] |
| 103 | Query=c148778/f1p1/1617 | 87 | [ref\|NP_065987.1\|](http://www.ncbi.nlm.nih.gov/protein/41327779?report=genbank&log$=protalign&blast_rank=1&RID=KC7ZBW8501R) | ATP-dependent RNA helicase DDX55 [Homo sapiens] |
| 104 | Query=c149258/f1p0/1046 | 88 | [ref\|NP_001157412.1\|](http://www.ncbi.nlm.nih.gov/protein/255653002?report=genbank&log$=protalign&blast_rank=7&RID=KC8MJ1CD01R) | glycogen phosphorylase， liver form isoform 2 [Homo sapiens] |
| 105 | Query=c149270/f1p3/2987 | 96 | ref\|NP_073153.1\| | anaphase-promoting complex subunit 1 [Homo sapiens] |
| 106 | Query=c149734/f3p15/1582 | 96 | [gb\|ABR93038.1\|](http://www.ncbi.nlm.nih.gov/protein/151327759?report=genbank&log$=protalign&blast_rank=1&RID=KCA446Z501R) | cytochrome c oxidase subunit I (mitochondrion) [Homo sapiens] |
| 107 | Query=c15145/f1p3/2464 | 93 | [ref\|NP_005560.1\|](http://www.ncbi.nlm.nih.gov/protein/5031869?report=genbank&log$=protalign&blast_rank=9&RID=KCBAG9W701R) | LIM domain kinase 2 isoform 2a [Homo sapiens] |
| 108 | Query=c15181/f1p6/2638 | 91 | [ref\|XP_005272325.1\|](http://www.ncbi.nlm.nih.gov/protein/530427333?report=genbank&log$=protalign&blast_rank=1&RID=KCBTYG8A01R) | cyclin-G-associated kinase isoform X5 [Homo sapiens] |
| 109 | Query=c15709/f1p2/1156 | 86 | [ref\|NP_001009905.1\|](http://www.ncbi.nlm.nih.gov/protein/57770468?report=genbank&log$=protalign&blast_rank=2&RID=KCDUP0UZ01R) | UDP-GlcNAc:betaGal beta-1,3-N-acetylglucosaminyltransferase-like protein 1 isoform a [Homo sapiens] |
| 110 | Query=c16257/f1p1/1511 | 83 | [ref\|NP_077014.3\|](http://www.ncbi.nlm.nih.gov/protein/971825950?report=genbank&log$=protalign&blast_rank=5&RID=KCJ5276C01R) | methyltransferase-like protein 22 [Homo sapiens] |
| 111 | Query=c16332/f1p6/2056 | 90 | [ref\|NP_060387.2\|](http://www.ncbi.nlm.nih.gov/protein/31542242?report=genbank&log$=protalign&blast_rank=1&RID=KCK6EEVP01R) | serine/threonine-protein phosphatase 2A regulatory subunit B'' subunit gamma isoform 1 [Homo sapiens] |
| 112 | Query=c16374/f1p7/994 | 98 | [ref\|XP_004060479.1\|](http://www.ncbi.nlm.nih.gov/protein/426388083?report=genbank&log$=protalign&blast_rank=1&RID=KCNE7EWK01R) | cytochrome b-c1 complex subunit Rieske,  mitochondrial-like isoform 2 [Gorilla gorilla gorilla] |
|  |  | 92 | [ref\|NP_005994.2](https://www.ncbi.nlm.nih.gov/protein/163644321?report=genbank&log$=protalign&blast_rank=4&RID=UN49YGCU014) | cytochrome b-c1 complex subunit Rieske,mitochondrial [Homo sapiens] |
| 113 | Query=c163976/f1p1/1634 | 77 | [gb\|EAW71137.1\|](http://www.ncbi.nlm.nih.gov/protein/119591543?report=genbank&log$=protalign&blast_rank=5&RID=KCP4BZR401R) | selenocysteine lyase， isoform CRA_c [Homo sapiens] |
| 114 | Query=c164016/f1p2/1402 | 83 | [ref\|NP_003701.1\|](http://www.ncbi.nlm.nih.gov/protein/4504329?report=genbank&log$=protalign&blast_rank=2&RID=KCPRECMV01R) | kunitz-type protease inhibitor 1 isoform 2 precursor [Homo sapiens] |
| 115 | Query=c164033/f1p3/1967 | 91 | [gb\|AAD17527.1\|](http://www.ncbi.nlm.nih.gov/protein/4335941?report=genbank&log$=protalign&blast_rank=2&RID=KDGG7KPH015) | leucine aminopeptidase [Homo sapiens] |
| 116 | Query=c164130/f1p0/2664 | 91 | [dbj\|BAA20846.2\|](http://www.ncbi.nlm.nih.gov/protein/6683697?report=genbank&log$=protalign&blast_rank=2&RID=KDH9RN31014) | KIAA0393 protein [Homo sapiens] |
| 117 | Query=c164504/f1p3/2638 | 79 | [ref\|NP_001154976.1\|](http://www.ncbi.nlm.nih.gov/protein/238859541?report=genbank&log$=protalign&blast_rank=2&RID=KDJG7K1J014) | delta-1-pyrroline-5-carboxylate dehydrogenase,  mitochondrial isoform b [Homo sapiens] |
| 118 | Query=c165388/f1p6/2519 | 87 | ref\|XP_006720852.1 | ubiquitin carboxyl-terminal hydrolase 3 isoform X1[Homo sapiens] |
| 119 | Query=c165487/f1p4/2500 | 94 | ref\|NP_079406.3 | putative hexokinase HKDC1[Homo sapiens] |
| 120 | Query=c165610/f1p2/1397 | 87 | gb\|AAC17469.1 | aldose reductase-like peptide[Homo sapiens] |
| 121 | Query=c165642/f1p1/2677 | 85 | ref\|NP_001154818.1 | E3 ubiquitin-protein ligase CHFR isoform 3[Homo sapiens] |
| 122 | Query=c165675/f1p2/1567 | 97 | gb\|AAA20046.1 | ubiquinol-cytochrome c reductase core[Homo sapiens] |
| 123 | Query=c165707/f1p4/3164 | 91 | ref\|XP_011529027.1\| | serine/threonine-protein phosphatase 6 regulatory subunit 2 isoform X6 [Homo sapiens] |
| 124 | Query=c165750/f1p1/1564 | 100 | ref\|XP_008972489.1 | NADH-ubiquinone oxidoreductase 75 kDa subunit, mitochondrial isoform X2 [Pan paniscus] |
|  |  | 90 | ref\|XP_016859677.1 | NADH-ubiquinone oxidoreductase 75 kDa subunit,mitochondrial isoform X1 [Homo sapiens] |
| 125 | Query=c165854/f1p2/2007 | 98 | ref\|XP_009428955.1 | 3 beta-hydroxysteroid dehydrogenase type 7 isoform X2 [Pongo abelii] |
|  |  | 86 | NP_079469.2 | 3 beta-hydroxysteroid dehydrogenase type 7 isoform a [Homo sapiens] |
| 126 | Query=c166183/f1p4/2490 | 96 | ref\|NP_055078.1 | ATP-dependent zinc metalloprotease YME1L1 isoform 3 [Homo sapiens] |
| 127 | Query=c166399/f1p0/2486 | 90 | ref\|NP_003492.2\| | peroxisomal acyl-coenzyme A oxidase 3 isoform a [Homo sapiens] |
| 128 | Query=c16654/f1p247/645 | 98 | gb\|ADV35242.1 | cytochrome c oxidase subunit II [Homo sapiens] |
| 129 | Query=c166597/f1p4/1175 | 98 | ref\|NP_001027563.1 | leucine carboxyl methyltransferase 1 isoform b [Homo sapiens] |
| 130 | Query=c16668/f2p0/1071 | 87 | ref\|NP_060182.1 | pyroglutamyl-peptidase 1 isoform 1 [Homo sapiens] |
| 131 | Query=c167129/f1p3/3363 | 95 | ref\|NP_001290198.1 | DNA-directed RNA polymerase II subunit RPB2 isoform 3[Homo sapiens] |
| 132 | Query=c167513/f1p2/2078 | 89 | ref\|NP_000472.2 | aminomethyltransferase， mitochondrial isoform 1 precursor[Homo sapiens] |
| 133 | Query=c167628/f1p4/1199 | 84 | gb\|AAH01778.1 | Uroporphyrinogen decarboxylase[Homo sapiens] |
| 134 | Query=c167961/f1p1/1857 | 78 | gb\|EAW50100.1 | hCG2040215[Homo sapiens] |
| 135 | Query=c167983/f1p8/1967 | 94 | ref\|NP_002942.2 | dolichyl-diphosphooligosaccharide--protein glycosyltransferase subunit 2 isoform 1 precursor[Homo sapiens] |
| 136 | Query=c168289/f1p0/1705 | 87 | ref\|XP_005266942.1\| | lactation elevated protein 1 isoform X1 [Homo sapiens] |
| 137 | Query=c168974/f1p0/2350 | 77 | gb\|AHW56613.1 | FANCD2/FANCI-associated nuclease 1 isoform A,partial[Homo sapiens] |
| 138 | Query=c169055/f1p3/2542 | 94 | NP_054733.2 | U5 small nuclear ribonucleoprotein 200 kDa helicase[Homo sapiens] |
| 139 | Query=c169079/f1p2/2195 | 73 | gb\|AAF36816.1\|AF175767_1 | putative selenocysteine lyase[Homo sapiens] |
| 140 | Query=c169413/f1p3/1992 | 93 | ref\|NP_000231.1 | amine oxidase [flavin-containing] A isoform 1 [Homo sapiens] |
| 141 | Query=c169424/f1p6/2349 | 96 | gb\|EAW63793.1 | N-acylsphingosine amidohydrolase (acid ceramidase) 1,isoform CRA_c [Homo sapiens] |
| 142 | Query=c169428/f1p4/2510 | 82 | ref\|NP_954592.1 | histone-arginine methyltransferase CARM1 |
| 143 | Query=c170148/f1p2/1893 | 94 | ref\|XP_011932844.1 | N6-adenosine-methyltransferase 70 kDa subunit isoform X1 [Cercocebus atys] |
|  |  | 90 | ref\|NP_062826.2 | N6-adenosine-methyltransferase 70 kDa subunit [Homo sapiens] |
| 144 | Query=c170206/f1p8/1431 | 89 | ref\|NP_002797.3 | 26S protease regulatory subunit 10B [Homo sapiens] |
| 145 | Query=c170260/f1p9/4192 | 94 | ref\|XP_011507753.1 | ceramide synthase 2 isoform X1 [Homo sapiens] |
| 146 | Query=c170418/f1p1/1722 | 93 | ref\|NP_000916.2 | pyruvate dehydrogenase E1 component subunit beta,  mitochondrial isoform 1 precursor [Homo sapiens] |
| 147 | Query=c170483/f1p13/1374 | 89 | ref\|NP_071898.2\| | E3 ubiquitin-protein ligase RNF25[Homo sapiens] |
| 148 | Query=c17050/f1p0/1916 | 91 | ref\|NP_079140.2\| | rRNA methyltransferase 1,mitochondrial precursor  [Homo sapiens] |
| 149 | Query=c17206/f1p3/913 | 90 | gb\|AAB60498.1 | [3-methyl-2-oxobutanoate dehydrogenase [lipoamide]] kinase,  mitochondrial isoform a precursor [Homo sapiens] |
| 150 | Query=c172487/f2p43/863 | 96 | gb\|ACA22445.1 | NADH dehydrogenase subunit 4 (mitochondrion) [Homo sapiens] |
| 151 | Query=c173139/f1p4/1252 | 97 | gb\|EAW66011.1 | chromosome 14 open reading frame 124,isoform CRA_c,partial[Homo sapiens] |
| 152 | Query=c17346/f1p6/1606 | 96 | [gb\|EAX07050.1\|](http://www.ncbi.nlm.nih.gov/protein/119627455?report=genbank&log$=protalign&blast_rank=1&RID=KGJ7RB7901R) | DNA methyltransferase 1 associated protein 1,isoform CRA_c,partial [Homo sapiens] |
| 153 | Query=c173707/f1p4/1384 | 95 | [ref\|NP_066964.1\|](http://www.ncbi.nlm.nih.gov/protein/10863945?report=genbank&log$=protalign&blast_rank=2&RID=KH561A4201R) | X-ray repair cross-complementing protein 5 [Homo sapiens] |
| 154 | Query=c174868/f1p5/1054 | 97 | [ref\|NP_001243728.1\|](http://www.ncbi.nlm.nih.gov/protein/378404908?report=genbank&log$=protalign&blast_rank=1&RID=KH6WFTVD01R) | glyceraldehyde-3-phosphate dehydrogenase isoform 2 [Homo sapiens] |
| 155 | Query=c17551/f1p3/2558 | 95 | [emb\|CAB72433.1\|](http://www.ncbi.nlm.nih.gov/protein/6966967?report=genbank&log$=protalign&blast_rank=1&RID=KH79T1KR01R) | dipeptidyl-peptidase III [Homo sapiens] |
| 156 | Query=c17680/f1p2/1557 | 71 | [pdb\|2BK4\|A](http://www.ncbi.nlm.nih.gov/protein/61680916?report=genbank&log$=protalign&blast_rank=4&RID=KHA0UJG601R)L | Chain A,Human Monoamine Oxidase B: I199f Mutant In Complex With Rasagiline |
| 157 | Query=c17775/f2p12/1066 | 95 | [gb\|ABR10799.1\|](http://www.ncbi.nlm.nih.gov/protein/148767727?report=genbank&log$=protalign&blast_rank=1&RID=KHB55PJM01R) | cytochrome c oxidase subunit I [Homo sapiens] |
| 158 | Query=c17849/f6p3/1070 | 92 | [gb\|AAB48437.1\|](http://www.ncbi.nlm.nih.gov/protein/1857419?report=genbank&log$=protalign&blast_rank=19&RID=KHDF2XZD01R) | protein arginine N-methyltransferase 2 [Homo sapiens] |
| 159 | Query=c17986/f1p2/3086 | 97 | NP_003472.2 | ubiquitin carboxyl-terminal hydrolase 5 isoform 2 [Homo sapiens] |
| 160 | Query=c181034/f1p3/1512 | 92 | [ref\|NP_001153706.1\|](http://www.ncbi.nlm.nih.gov/protein/237681111?report=genbank&log$=protalign&blast_rank=5&RID=KHGWWDYW01R) | sodium/potassium-transporting ATPase subunit alpha-1 isoform d [Homo sapiens] |
| 161 | Query=c181673/f6p26/770 | 96 | [gb\|AIT96835.1\|](http://www.ncbi.nlm.nih.gov/protein/698352614?report=genbank&log$=protalign&blast_rank=1&RID=KHJ91HKX01R) | cytochrome b,partial (mitochondrion) [Homo sapiens] |
| 162 | Query=c18200/f1p1/922 |  | [ref\|NP_001305855.1\|](http://www.ncbi.nlm.nih.gov/protein/974576755?report=genbank&log$=protalign&blast_rank=1&RID=KHK3U1X701R) | ribulose-phosphate 3-epimerase isoform 6 [Homo sapiens] |
| 163 | Query=c18225/f1p14/1328 | 88 | [ref\|NP_071898.2\|](http://www.ncbi.nlm.nih.gov/protein/34878787?report=genbank&log$=protalign&blast_rank=1&RID=KHKN8KKH01R) | E3 ubiquitin-protein ligase RNF25 [Homo sapiens] |
| 164 | Query=c18243/f2p11/905 | 95 | [ref\|NP_037543.1\|](http://www.ncbi.nlm.nih.gov/protein/7524346?report=genbank&log$=protalign&blast_rank=4&RID=KHM5026S01R) | adenylate kinase 2,  mitochondrial isoform b [Homo sapiens] |
| 165 | Query=c184433/f49p46/1139 | 96 | [dbj\|BAE09988.1\|](http://www.ncbi.nlm.nih.gov/protein/71012349?report=genbank&log$=protalign&blast_rank=1&RID=KHN3DW9J01R) | cytochrome b [Homo sapiens] |
| 166 | Query=c185039/f4p80/1504 | 85 | [gb\|AKI69719.1\|](http://www.ncbi.nlm.nih.gov/protein/823669014?report=genbank&log$=protalign&blast_rank=1&RID=KHR9E9GB01R) | ALDOA,partial [synthetic construct] |
| 167 | Query=c185044/f5p44/1470 | 89 | [ref\|NP_000025.1\|](http://www.ncbi.nlm.nih.gov/protein/4557305?report=genbank&log$=protalign&blast_rank=3&RID=KHRPCWWM01R) | fructose-bisphosphate aldolase A isoform 1 [Homo sapiens] |
| 168 | Query=c185664/f1p2/2241 | 83 | [ref\|NP_001240.1\|](http://www.ncbi.nlm.nih.gov/protein/4557427?report=genbank&log$=protalign&blast_rank=1&RID=KHSY0HFR01R) | ectonucleoside triphosphate diphosphohydrolase 5 isoform 1 precursor [Homo sapiens] |
| 169 | Query=c185771/f1p1/2257 | 84 | [ref\|NP_001243266.1\|](http://www.ncbi.nlm.nih.gov/protein/373432620?report=genbank&log$=protalign&blast_rank=4&RID=KNU2605W014) | chromodomain-helicase-DNA-binding protein 1-like isoform 4 [Homo sapiens] |
| 170 | Query=c185907/f1p1/2088 | 88 | [ref\|NP_777582.4\|](http://www.ncbi.nlm.nih.gov/protein/525507394?report=genbank&log$=protalign&blast_rank=7&RID=KNUV36W6015) | uncharacterized aarF domain-containing protein kinase 5 [Homo sapiens] |
| 171 | Query=c186517/f1p10/1949 | 93 | [ref\|NP_000389.1\|](http://www.ncbi.nlm.nih.gov/protein/4503327?report=genbank&log$=protalign&blast_rank=1&RID=KPGW7T9701R) | NADH-cytochrome b5 reductase 3 isoform 1 [Homo sapiens] |
| 172 | Query=c186998/f1p7/3234 | 95 | [ref\|NP_079406.3\|](http://www.ncbi.nlm.nih.gov/protein/156151420?report=genbank&log$=protalign&blast_rank=1&RID=KS123E5J015) | putative hexokinase HKDC1 [Homo sapiens] |
| 173 | Query=c187003/f1p0/2509 | 87 | [ref\|NP_078782.3\|](http://www.ncbi.nlm.nih.gov/protein/40255043?report=genbank&log$=protalign&blast_rank=2&RID=KS1GBR7F014) | beta-galactosidase-1-like protein isoform 1 precursor [Homo sapiens] |
| 174 | Query=c187168/f1p1/1441 | 99 | ref\|XP_009455726.2 | phytanoyl-CoA dioxygenase domain-  containing protein 1 isoform X6 [Pan troglodytes] |
|  |  | 86 | ref\|NP_001094346.1 | phytanoyl-CoA dioxygenase domain-containing protein 1 isoform a [Homo sapiens] |
| 175 | Query=c187431/f1p0/1476 | 88 | ref\|XP_010370213.1 | DNA methyltransferase 1-associated protein 1 isoform X1 [Rhinopithecus roxellana] |
|  |  | 83 | gb\|EAX07050.1 | DNA methyltransferase 1 associated protein 1, isoform CRA_c, partial [Homo sapiens] |
| 176 | Query=c18747/f1p6/2442 | 93 | [ref\|NP_004448.2\|](http://www.ncbi.nlm.nih.gov/protein/42794752?report=genbank&log$=protalign&blast_rank=2&RID=KS7NX3VS014) | long-chain-fatty-acid--CoA ligase 3 [Homo sapiens] |
| 177 | Query=c187487/f1p0/3275 | 98 | [ref\|NP_963861.1\|](http://www.ncbi.nlm.nih.gov/protein/42490760?report=genbank&log$=protalign&blast_rank=1&RID=KSA285V9014) | M-phase inducer phosphatase 1 isoform b [Homo sapiens] |
| 178 | Query=c187884/f1p1/2490 | 97 | [ref\|XP_009246185.1\|](http://www.ncbi.nlm.nih.gov/protein/686741280?report=genbank&log$=protalign&blast_rank=5&RID=KSAN15GC014) | glutaminase liver isoform， mitochondrial isoform X3 [Pongo abelii] |
|  |  | 96 | ref\|NP_001267727.1 | glutaminase liver isoform， mitochondrial isoform 4 [Homo sapiens] |
| 179 | Query=c187918/f1p1/1760 | 81 | ref\|XP_016878946.1 | chitobiosyldiphosphodolichol beta-  mannosyltransferase isoform X1 [Homo sapiens] |
| 180 | Query=c188058/f1p0/2086 | 90 | XP_011540435.1 | epidermal growth factor receptor kinase substrate 8-like protein 3 isoform X7 [Homo sapiens] |
| 181 | Query=c188178/f1p1/1101 | 86 | NP_068806.1 | mannose-1-phosphate guanyltransferase beta isoform 2 [Homo sapiens] |
| 182 | Query=c188321/f1p0/2006 | 77 | NP_002758.1 | phosphoribosyl pyrophosphate synthase-associated protein 2 isoform 1 [Homo sapiens] |
| 183 | Query=c188375/f1p6/1572 | 98 | ELW66816.1 | Adenylate kinase isoenzyme 4,mitochondrial [Tupaiachinensis] |
|  |  | 82 | gb\|AAH40224.1 | Adenylate kinase 3-like 1 [Homo sapiens] |
| 184 | Query=c188521/f1p9/3283 | 96 | ref\|NP_004530.1\| | asparagine--tRNA ligase, cytoplasmic [Homo sapiens] |
| 185 | Query=c188556/f1p2/975 | 92 | NP_001303965.1 | dolichol-phosphate mannosyltransferase subunit 1 isoform 4 [Homo sapiens] |
| 186 | Query=c188713/f1p2/2454 | 96 | NP_001281261.1 | succinate dehydrogenase [ubiquinone] flavoprotein subunit,mitochondrial isoform 2 [Homo sapiens] |
| 187 | Query=c188799/f1p1/3021 | 84 | NP_001596.2 | alanine--tRNA ligase,  cytoplasmic [Homo sapiens] |
| 188 | Query=c188872/f1p2/2411 | 75 | XP_006719461.1 | methionine--tRNA ligase,  cytoplasmic isoform X1 [Homo sapiens] |
| 189 | Query=c188874/f1p4/1821 | 96 | NP_000158.1 | glycerol kinase isoform b [Homo sapiens] |
| 190 | Query=c189217/f1p7/3064 | 90 | NP_848934.1 | glycerol-3-phosphate acyltransferase 4 [Homo sapiens] |
| 191 | Query=c189514/f1p4/2175 | 92 | NP_060767.2 | peptide-N(4)-(N-acetyl-beta-glucosaminyl)asparagine amidase isoform 1 [Homo sapiens] |
| 192 | Query=c189604/f1p25/2823 | 89 | NP_055365.2 | caspase recruitment domain-containing protein 10 [Homo sapiens] |
| 193 | Query=c189722/f1p3/1289 | 90 | gb\|AAQ74775.1 | putative glycosyltransferase [Homo sapiens] |
| 194 | Query=c18998/f1p0/2585 | 77 | NP_003791.3 | mRNA-capping enzyme isoform a [Homo sapiens] |
| 195 | Query=c190121/f1p2/3428 | 83 | NP_003174.3 | disintegrin and metalloproteinase domain-containing protein 17 preproprotein [Homo sapiens] |
| 196 | Query=c190273/f1p9/1855 | 95 | ref\|NP_001305754.1 | beta-hexosaminidase subunit alpha isoform 1 precursor [Homo sapiens] |
| 197 | Query=c19042/f2p4/1456 | 91 | CAA88733.1 | helicase [Homo sapiens] |
| 198 | Query=c190926/f1p5/3408 | 98 | EAW55598.1 | hypothetical protein FLJ20297,isoform CRA_b [Homo sapiens] |
| 199 | Query=c193281/f1p12/899 | 82 | ALC78884.1 | fructose-bisphosphate aldolase A,partial [Homo sapiens] |
| 200 | Query=c19430/f1p4/2002 | 98 | NP_115497.4 | magnesium transporter protein 1 [Homo sapiens] |
| 201 | Query=c19676/f1p13/1874 | 96 | NP_002100.2 | histidine--tRNA ligase,  cytoplasmic isoform 1 [Homo sapiens] |
| 202 | Query=c2002/f7p6/959 | 88 | NP_006432.1 | 5-formyltetrahydrofolate cyclo-ligase isoform a [Homo sapiens] |
| 203 | Query=c20148/f1p0/2423 | 86 | XP_011544194.1 | DNA-directed RNA polymerase III subunit RPC5 isoform X2 [Homo sapiens] |
| 204 | Query=c20177/f1p1/1187 | 98 | XP_009245103.1 | proteasomal ATPase-associated factor 1 isoform X3 [Pongo abelii] |
|  |  | 75 | NP_001254732.1 | proteasomal ATPase-associated factor 1 isoform 1 [Homo sapiens] |
| 205 | Query=c202380/f19p53/1402 | 94 | AAP48010.1 | NADH dehydrogenase subunit 1 [Homo sapiens] |
| 206 | Query=c203757/f1p3/1467 | 85 | NP_001269705.1 | DNA-directed RNA polymerase I subunit RPA2 isoform 5 [Homo sapiens] |
| 207 | Query=c205119/f1p3/2190 | 80 | NP_001159828.1 | serine hydroxymethyltransferase,mitochondrial isoform 2 precursor [Homo sapiens] |
| 208 | Query=c20712/f8p9/1030 | 90 | NP_057576.2 | ran guanine nucleotide release factor isoform A [Homo sapiens] |
| 209 | Query=c217183/f1p210/578 | 98 | ABB79160.1 | cytochrome c oxidase subunit II (mitochondrion) [Homo sapiens] |
| 210 | Query=c217774/f1p8/579 | 98 | AIU60013.1 | ATP synthase F0 subunit 6 (mitochondrion) [Homo sapiens] |
| 211 | Query=c219110/f1p1/580 | 77 | BAA95608.1 | phenylalanyl tRNAsynthetase [Homo sapiens] |
| 212 | Query=c24196/f4p8/866 | 80 | 1IZ2 | Chain A, Interactions Causing The Kinetic Trap In Serpin Protein Folding |
|  |  | 80 | 1HP7 | Chain A, A 2.1 Angstrom Structure Of An Uncleaved Alpha-1-Antitrypsin Shows Variability Of The Reactive Center And Other Loops |
|  |  | 80 | AGW25372.1 | alpha-1-proteinase inhibitor F51L, partial [synthetic construct] |
| 213 | Query=c24693/f5p5/1559 | 81 | NP_001243572.1 | leukotriene A-4 hydrolase isoform 2 [Homo sapiens] |
| 218 | Query=c25038/f1p6/1818 | 82 | NP_004175.2 | tryptophan--tRNA ligase, cytoplasmic isoform a [Homo sapiens] |
| 214 | Query=c25424/f1p3/1090 | 86 | NP_001036017.1 | putative deoxyribonuclease TATDN3 isoform 1 [Homo sapiens] |
| 215 | Query=c25805/f1p1/786 | 92 | AIH15156.1 | NADH dehydrogenase subunit 5, partial (mitochondrion) [Homo sapiens] |
| 216 | Query=c26473/f3p1/809 | 87 | NP_001370.1 | DNA (cytosine-5)-methyltransferase 1 isoform b [Homo sapiens] |
| 217 | Query=c26499/f1p1/1717 | 74 | AAQ88943.1 | C20orf31 [Homo sapiens] |
| 218 | Query=c27100/f1p3/2408 | 94 | NP_001036068.1 | lysine-specific demethylase 5A [Homo sapiens] |
| 219 | Query=c13783/f25p9/2152 | 92 | Dbj\|BAD92022.1 | phosphoribosylglycinamide formyltransferase,phosphoribosylglycinamide synthetase, phosphoribosylaminoimidazole synthetase isoform 1 variant, partial [Homo sapiens] |
| 220 | Query=c13842/f5p22/1826 | 96 | ref\|XP_013972311.1 | 14-3-3 protein epsilon isoform X1 [Canis lupus familiaris] |
|  |  | 95 | ref\|NP_006752.1 | 14-3-3 protein epsilon [Homo sapiens] |
| 221 | Query=c13936/f1p2/1906 | 97 | ref\|NP_000301.1 | palmitoyl-protein thioesterase 1 isoform 1 precursor [Homo sapiens] |
| 222 | Query=c14196/f2p0/1989 | 100 | ref\|XP_008969934.1 | E3 ubiquitin-protein ligase ARIH2 isoform X2 [Pan paniscus] |
|  |  | 94 | ref\|XP_011531572.1 | E3 ubiquitin-protein ligase ARIH2 isoform X3 [Homo sapiens] |
| 223 | Query=c142497/f2p4/2021 | 99 | ref\|XP_005577299.1 | histone acetyltransferase KAT5 isoform X3 [Macaca fascicularis] |
|  |  | 98 | ref\|NP_006379.2 | histone acetyltransferase KAT5 isoform 2 [Homo sapiens] |
| 224 | Query=c143137/f1p5/1999 | 98 | ref\|NP_001645.1 | serine/threonine-protein kinase A-Raf isoform 1 [Homo sapiens] |
| 225 | Query=c143719/f1p3/2121 | 97 | ref\|NP_857593.1 | kunitz-type protease inhibitor 1 isoform 1 precursor [Homo sapiens] |
| 226 | Query=c144623/f1p0/1677 | 100 | ref\|XP_004052189.1 | beta,beta-carotene 9',10'-oxygenase-like [Gorilla gorilla gorilla] |
|  |  | 97 | ref\|NP_002993.1 | succinate dehydrogenase [ubiquinone] cytochrome b small subunit, mitochondrial isoform a precursor [Homo sapiens] |
| 227 | Query=c145269/f1p3/1594 | 79 | emb\|CAO78188.1 | protein disulfide isomerase family A, member 2 [Homo sapiens] |
| 228 | Query=c145428/f1p1/1145 | 100 | ref\|XP_010371888.1 | UDP-GlcNAc:betaGal beta-1,3-N-acetylglucosaminyltransferase-like protein 1 [Rhinopithecus roxellana] |
|  |  | 96 | ref\|XP_016879694.1 | UDP-GlcNAc:betaGal beta-1,3-N-acetylglucosaminyltransferase-like protein 1 isoform X3 [Homo sapiens] |
| 229 | Query=c145560/f1p2/1667 | 94 | ref\|NP_001309278.1 | exosome complex exonuclease RRP44 isoform d [Homo sapiens] |
| 230 | Query=c145905/f2p8/1472 | 99 | ref\|XP_008963854.2 | cysteine protease ATG4B isoform X2 [Pan paniscus] |
|  |  | 91 | ref\|NP_037457.3 | cysteine protease ATG4B isoform a [Homo sapiens] |
| 231 | Query=c145974/f1p5/1300 | 85 | dbj\|BAG09639.1 | tubulin--tyrosine ligase-like protein 4, partial [synthetic construct] |
|  |  | 85 | gb\|AAH21707.1 | Tubulin tyrosine ligase-like family, member 4 [Homo sapiens] |
| 232 | Query=c146442/f3p11/2420 | 100 | ref\|XP_003952385.1 | anaphase-promoting complex subunit 5 isoform X4 [Pan troglodytes] |
|  |  | 98 | ref\|NP_057321.2 | anaphase-promoting complex subunit 5 isoform a [Homo sapiens] |
| 233 | Query=c146713/f1p12/1120 | 95 | ref\|XP_011526744.1 | very-long-chain enoyl-CoA reductase isoform X2 [Homo sapiens] |
| 234 | Query=c146776/f1p0/2168 | 94 | gb\|EAX10316.1 | taspase, threonine aspartase, 1, isoform CRA_c [Homo sapiens] |
|  |  | 89 | ref\|XP_016883419.1 | threonine aspartase 1 isoform X3 [Homo sapiens] |
| 235 | Query=c147866/f1p3/1854 | 96 | gb\|EAW77285.1 | sorbitol dehydrogenase, isoform CRA_b [Homo sapiens] |
| 236 | Query=c148269/f1p0/1087 | 90 | ref\|NP_000021.1 | serine--pyruvate aminotransferase [Homo sapiens] |
| 237 | Query=c148663/f1p1/2567 | 97 | ref\|NP_057314.2 | cytochrome b5 reductase 4 [Homo sapiens] |
| 238 | Query=c148671/f1p3/1120 | 99 | ref\|XP_008962762.1 | 2-oxoglutarate and iron-dependent oxygenase domain-containing protein 2 isoform X2 [Pan paniscus] |
|  |  | 94 | ref\|NP_001291762.1 | 2-oxoglutarate and iron-dependent oxygenase domain-containing protein 2 Isoform 1 [Homo sapiens] |
| 239 | Query=c149155/f1p2/1082 | 100 | ref\|XP_009190228.1 | GDP-Man:Man(3)GlcNAc(2)-PP-Dol alpha-1,2-  mannosyltransferase isoform X2 [Papio anubis] |
|  |  | 97 | ref\|NP_001004127.2 | GDP-Man:Man(3)GlcNAc(2)-PP-Dol alpha-1,2-  mannosyltransferase [Homo sapiens] |
| 240 | Query=c149518/f1p2/1069 | 97 | ref\|NP_001274076.1 | 2-acylglycerol O-acyltransferase 3 isoform b [Homo sapiens] |
|  |  | 94 | ref\|XP_011514429.1 | 2-acylglycerol O-acyltransferase 3 isoform X3 [Homo sapiens] |
| 241 | Query=c15045/f4p16/1331 | 97 | ref\|XP_005245255.1 | antithrombin-III isoform X1 [Homo sapiens] |
| 242 | Query=c15280/f2p3/2569 | 78 | gb\|AAF81422.1 | teratoma-associated tyrosine kinase, partial [Homo sapiens] |
| 243 | Query=c15398/f2p5/1270 | 96 | [ref\|XP_005276400.1](https://www.ncbi.nlm.nih.gov/protein/530436290?report=genbank&log$=protalign&blast_rank=1&RID=UNYVTCWH014) | monofunctional C1-tetrahydrofolate synthase, mitochondrial-like isoform X3 [Homo sapiens] |
| 244 | Query=c15617/f3p8/1467 | 96 | ref\|XP_008996025.1 | phosphoribosyl pyrophosphate synthase-associated protein 1 isoform X2 [Callithrix jacchus] |
|  |  | 96 | ref\|NP_002757.2 | phosphoribosyl pyrophosphate synthase-associated protein 1 [Homo sapiens] |
| 245 | Query=c16024/f1p3/690 | 96 | gb\|AIH15148.1 | NADH dehydrogenase subunit 1, partial (mitochondrion) [Homo sapiens] |
| 246 | Query=c16074/f1p6/2253 | 98 | emb\|CAK18174.1 | fatty acid coenzyme A ligase 5 [Homo sapiens] |
|  |  | 96 | gb\|EAW49534.1 | acyl-CoA synthetase long-chain family member 5, isoform CRA_c [Homo sapiens] |
| 247 | Query=c16105/f1p6/1908 | 88 | ref\|XP_011527516.1 | monoacylglycerol lipase ABHD12 isoform X2 [Homo sapiens] |
| 248 | Query=c16117/f1p0/1061 | 96 | ref\|XP_011506904.1 | sulfotransferase 1A3 isoform X5 [Homo sapiens] |
| 254 | Query=c10014/f1p2/1726 | 88 | gb\|AAO84339.1\| | tafazzin short form [Homo sapiens] |
| 249 | Query=c102057/f1p0/1318 | 96 | ref\|NP_036346.1\| | alpha-1,3-mannosyl-glycoprotein 4-beta-N-acetylglucosaminyltransferase A isoform 1 [Homo sapiens] |
| 250 | Query=c102238/f1p9/1056 | 98 | ref\|NP_002703.1\| | protein phosphatase 1 regulatory subunit 7 isoform 1 [Homo sapiens] |
| 251 | Query=c102849/f2p2/2258 | 96 | dbj\|BAG70114.1\| | MAP kinase interacting serine/threonine kinase 1 [Homo sapiens] |
| 252 | Query=c103415/f1p1/1217 | 98 | ref\|XP_005254779.1\| | PREDICTED: signal peptide peptidase-like 2A isoform X1 [Homo sapiens] |
| 253 | Query=c103975/f1p0/1382 | 98 | ref\|XP_011900618.1\| | PREDICTED: serine/threonine-protein kinase WNK2 isoform X7 [Cercocebus atys] |
|  |  | 82 | gb\|AAH37965.1\| | WNK2 protein, partial [Homo sapiens] |
| 254 | Query=c104123/f2p1/1934 | 99 | ref\|XP_008972096.1\| | PREDICTED: A-kinase anchor protein 13 isoform X5 [Pan paniscus] |
|  |  | 97 | dbj\|BAB62913.1\| | guanine nucleotide exchange factor Lbc [Homo sapiens] |
| 255 | Query=c104376/f1p1/1536 | 92 | ref\|NP_775891.2\| | hexosaminidase D [Homo sapiens] |
| 256 | Query=c104785/f1p4/2498 | 87 | ref\|XP_006722669.1\| | PREDICTED: ATP-dependent RNA helicase DDX39A isoform X2 [Homo sapiens] |
| 257 | Query=c105305/f1p3/1029 | 86 | ref\|NP_054891.2\| | 14 kDa phosphohistidine phosphatase isoform 3 [Homo sapiens] |
| 258 | Query=c105390/f1p6/750 | 95 | ref\|XP_004025446.1\| | PREDICTED: adenylate kinase 2, mitochondrial [Gorilla gorilla gorilla] |
|  |  | 94 | ref\|NP_037543.1\| | adenylate kinase 2, mitochondrial isoform b [Homo sapiens] |
| 259 | Query=c105494/f1p0/1279 | 97 | ref\|NP_004530.1\| | asparagine--tRNA ligase, cytoplasmic [Homo sapiens] |
| 260 | Query=c107648/f1p1/1406 | 92 | ref\|XP_009001624.1\| | PREDICTED:alpha-N-acetyl-neuraminyl-2,3-beta-galactosyl-1,3-N-acetyl-galactosaminide alpha-2,6-sialyltransferase isoform X2 [Callithrix jacchus] |
|  |  | 83 | ref\|NP_778204.1\| | alpha-N-acetyl-neuraminyl-2,3-beta-galactosyl-1,3-N-acetyl-galactosaminide alpha-2,6-sialyltransferase isoform a [Homo sapiens] |
| 261 | Query=c11319/f1p8/1115 | 96 | ref\|XP_012327468.1\| | PREDICTED: ubiquitin carboxyl-terminal hydrolase isozyme L3 isoform X2 [Aotus nancymaae] |
|  |  | 95 | ref\|NP_005993.1\| | ubiquitin carboxyl-terminal hydrolase isozyme L3 isoform 2 [Homo sapiens] |
| 262 | Query=c122901/f1p2/1041 | 91 | [ref\|NP_000886.1\|](https://www.ncbi.nlm.nih.gov/protein/4505029?report=genbank&log$=protalign&blast_rank=2&RID=UNPPWECA016) | leukotriene A-4 hydrolase isoform 1 [Homo sapiens] |
| 263 | Query=c123446/f1p0/1342 | 94 | [ref\|XP_009199749.1\|](https://www.ncbi.nlm.nih.gov/protein/685526757?report=genbank&log$=protalign&blast_rank=2&RID=UNRKYAV1014) | PREDICTED: GMP synthase [glutamine-hydrolyzing] isoform X3 [Papio anubis] |
| 264 | Query=c123650/f1p5/1864 | 91 | [ref\|NP_000169.1\|](https://www.ncbi.nlm.nih.gov/protein/4504169?report=genbank&log$=protalign&blast_rank=2&RID=UNRVF88K01R) | glutathione synthetase [Homo sapiens] |
| 265 | Query=c123885/f1p2/1386 | 97 | [ref\|XP_011523855.1\|](https://www.ncbi.nlm.nih.gov/protein/767996724?report=genbank&log$=protalign&blast_rank=1&RID=UNSDBP1D01R) | PREDICTED: mediator of RNA polymerase II transcription subunit 13 isoform X3 [Homo sapiens] |
| 266 | Query=c123905/f1p3/1066 | 96 | [ref\|NP_995583.1\|](https://www.ncbi.nlm.nih.gov/protein/45580738?report=genbank&log$=protalign&blast_rank=1&RID=UNSJ3DZU01R) | dehydrodolichyl diphosphate synthase complex subunit DHDDS isoform 1 [Homo sapiens] |
| 267 | Query=c123974/f1p4/1218 | 93 | [gb\|AAG09682.1\|AF183413_1](https://www.ncbi.nlm.nih.gov/protein/9963765?report=genbank&log$=protalign&blast_rank=2&RID=UNSP8RM2014) | dolichyl-phosphate beta-glucosyltransferase [Homo sapiens] |
| 268 | Query=c124105/f1p4/1458 | 96 | [pdb\|3ECR\|A](https://www.ncbi.nlm.nih.gov/protein/208435725?report=genbank&log$=protalign&blast_rank=1&RID=UNSUW76F014) | Chain A, Structure Of Human Porphobilinogen Deaminase |
| 269 | Query=c124380/f1p1/1654 | 98 | [gb\|EAW61662.1\|](https://www.ncbi.nlm.nih.gov/protein/119582066?report=genbank&log$=protalign&blast_rank=1&RID=UNSYYVCP016) | Ras-GTPase-activating protein SH3-domain-binding protein, isoform CRA_c [Homo sapiens] |
| 270 | Query=c125641/f1p21/1285 | 90 | [gb\|AAC03787.1\|](https://www.ncbi.nlm.nih.gov/protein/2906146?report=genbank&log$=protalign&blast_rank=1&RID=UNV7C7Y001R) | malate dehydrogenase precursor [Homo sapiens] |
| 271 | Query=c125972/f1p0/1071 | 95 | r[ef\|NP_001676.2\|](https://www.ncbi.nlm.nih.gov/protein/18644883?report=genbank&log$=protalign&blast_rank=2&RID=UNVAW8PF01R) | ATP synthase-coupling factor 6, mitochondrial isoform a precursor [Homo sapiens] |
| 272 | Query=c126319/f2p2/1405 | 84 | ref\|NP_835470.1\| | 2-acylglycerol O-acyltransferase 3 isoform a [Homo sapiens] |
| 273 | Query=c126616/f1p2/3519 | 94 | ref\|XP_006712675.1\| | ubiquitin carboxyl-terminal hydrolase 40 isoform X2 [Homo sapiens] |
| 274 | Query=c127451/f1p1/1131 | 98 | [gb\|AAP36671.1\|](https://www.ncbi.nlm.nih.gov/protein/30584837?report=genbank&log$=protalign&blast_rank=2&RID=UNZARSPR016) | Homo sapiens farnesyl-diphosphate farnesyltransferase 1, partial [synthetic construct] |
| 275 | Query=c127807/f2p0/777 | 78 | [ref\|NP_006224.1\|](https://www.ncbi.nlm.nih.gov/protein/5453930?report=genbank&log$=protalign&blast_rank=29&RID=UNZP256J016) | DNA-directed RNA polymerase II subunit RPB9 [Homo sapiens] |
| 276 | Query=c128295/f1p53/1279 | 93 | ref\|NP_002801.1\| | 26S proteasome non-ATPase regulatory subunit 4 [Homo sapiens] |
| 277 | Query=c128924/f1p2/1941 | 94 | [ref\|NP_060687.2\|](https://www.ncbi.nlm.nih.gov/protein/222537737?report=genbank&log$=protalign&blast_rank=1&RID=URN2B59A014) | ER degradation-enhancing alpha-mannosidase-like protein 2 isoform 1 precursor [Homo sapiens] |
| 278 | Query=c16261/f2p1/972 | 97 | ref\|NP_001065278.1 | NADH dehydrogenase [ubiquinone] 1 beta subcomplex subunit 11, mitochondrial precursor [Pan troglodytes] |
|  |  | 97 | ref\|NP_001129470.1 | NADH dehydrogenase [ubiquinone] 1 beta subcomplex subunit 11, mitochondrial isoform 2 [Homo sapiens] |
| 279 | Query=c164094/f1p1/1384 | 92 | [ref\|NP_060679.1](https://www.ncbi.nlm.nih.gov/protein/8922652?report=genbank&log$=protalign&blast_rank=1&RID=UN525FE7014) | ADP-ribosylation factor GTPase-activating protein 1 isoform a [Homo sapiens] |
| 280 | Query=c164873/f3p26/1488 | 97 | ref\|NP_005778.1 | dol-P-Man:Man(5)GlcNAc(2)-PP-Dol alpha-1,3-mannosyltransferase isoform a [Homo sapiens] |
| 281 | Query=c164957/f1p2/1416 | 95 | emb\|CAE54074.1 | putative uridine kinase [Homo sapiens] |
| 282 | Query=c165013/f1p2/1359 | 90 | ref\|XP_016859677.1 | NADH-ubiquinone oxidoreductase 75 kDa subunit, mitochondrial isoform X1 [Homo sapiens] |
| 283 | Query=c16582/f1p0/1301 | 96 | ref\|XP_006717225.1 | nicotinamide riboside kinase 1 isoform X1 [Homo sapiens] |
| 284 | Query=c166029/f1p5/2121 | 92 | ref\|NP_001096029.1 | phospholipid phosphatase 5 isoform 1 [Homo sapiens] |
| 285 | Query=c166230/f2p13/1536 | 95 | ref\|XP_011513733.1 | DNA polymerase delta subunit 2 isoform X1 [Homo sapiens] |
| 286 | Query=c167189/f1p2/2509 | 97 | ref\|NP_060579.3 | poly(A) RNA polymerase, mitochondrial precursor [Homo sapiens] |
| 287 | Query=c167971/f1p1/1965 | 97 | ref\|XP_004064721.1 | ribose-phosphate pyrophosphokinase 1 isoform 2 [Gorilla gorilla gorilla] |
|  |  | 83 | ref\|NP_002755.1 | ribose-phosphate pyrophosphokinase 1 isoform 1 [Homo sapiens] |
| 288 | Query=c170434/f1p10/1532 | 93 | ref\|NP_000916.2 | pyruvate dehydrogenase E1 component subunit , mitochondrial isoform 1 precursor [Homo sapiens] |
| 289 | Query=c191186/f1p1/2824 | 92 | ref\|NP_057360.2 | serine/threonine-protein kinase Sgk2 isoform beta [Homo sapiens] |
| 290 | Query=c189702/f1p9/2923 | 95 | ref\|NP_000176.2 | heparin cofactor 2 precursor [Homo sapiens] |
| 291 | Query=c217020/f1p0/510 | 95 | ref\|NP_002406.1 | macrophage migration inhibitory factor |
| 292 | Query=c26685/f1p0/1019 | 98 | [ref\|XP_012366333.1\|](https://www.ncbi.nlm.nih.gov/protein/820982291?report=genbank&log$=protalign&blast_rank=1&RID=USHKX3WR014) | PREDICTED: PIN2/TERF1-interacting telomerase inhibitor 1 isoform X1 [Nomascus leucogenys] |
| 293 | Query=c27565/f3p0/1136 | 99 | ref\|XP_011946644.1 | phosphoenolpyruvate carboxykinase [GTP], mitochondrial isoform X1 [Cercocebus atys] |
| 294 | Query=c2768/f1p0/1111 | 88 | dbj\|BAA06808.1 | alanyl-tRNA synthetase [Homo sapiens] |
| 295 | Query=c27947/f2p14/1808 | 90 | dbj\|BAA11928.1 | ER-60 protease [Homo sapiens] |
| 296 | Query=c29322/f2p1/1217 | 89 | gb\|AAH05054.2 | Methylmalonic aciduria (cobalamin deficiency) cblB type [Homo sapiens] |
| 297 | Query=c29565/f1p0/798 | 85 | gb\|AAH02714.2 | Glycerophosphodiester phosphodiesterase domain containing 3 [Homo sapiens] |
| 298 | Query=c31040/f1p2/3454 | 96 | [ref\|NP_001596.2\|](https://www.ncbi.nlm.nih.gov/protein/109148542?report=genbank&log$=protalign&blast_rank=1&RID=USJNB2F4014) | alanine--tRNA ligase, cytoplasmic [Homo sapiens] |
| 299 | Query=c31152/f1p4/2594 | 96 | ref\|NP_006706.2 | alpha-mannosidase 2C1 isoform 1 [Homo sapiens] |
| 300 | Query=c3205/f1p1/854 | 100 | ref\|XP_009241997.1 | phosphatidate phosphatase PPAPDC1B isoform X2 [Pongo abelii] |
| 301 | Query=c3382/f1p6/798 | 94 | ref\|NP_000467.1 | adenylate kinase isoenzyme 1 isoform 1 [Homo sapiens] |
| 302 | Query=c36672/f2p6/935 | 87 | gb\|AAQ13425.1 | phosphatidylethanolamine-N-methyltransferase-like protein [Homo sapiens] |
| 303 | Query=c36821/f1p3/1884 | 95 | ref\|NP_878256.1 | geranylgeranyl transferase type-2 subunit alpha [Homo sapiens] |
| 304 | Query=c37371/f2p6/1794 | 95 | ref\|NP_000276.2 | xaa-Pro dipeptidase isoform 1 [Homo sapiens] |
| 305 | Query=c37407/f1p5/1057 | 85 | ref\|NP_054783.2 | N-terminal Xaa-Pro-Lys N-methyltransferase 1 isoform a [Homo sapiens] |
| 306 | Query=c37483/f1p3/1454 | 92 | ref\|NP_009213.1\| | E3 ubiquitin-protein ligase RNF13 [Homo sapiens] |
| 307 | Query=c37951/f1p4/2278 | 98 | ref\|NP_060705.2 | cytosolic non-specific dipeptidase isoform 1 [Homo sapiens] |
| 308 | Query=c39116/f1p1/1514 | 98 | ref\|NP_001311234.1\| | dolichyl-diphosphooligosaccharide--protein glycosyltransferase subunit 2 isoform 8 precursor [Homo sapiens] |
| 309 | Query=c40057/f1p0/962 | 84 | [ref\|XP_006906067.2\|](https://www.ncbi.nlm.nih.gov/protein/989897813?report=genbank&log$=protalign&blast_rank=1&RID=UN5PAA0W016) | mediator of RNA polymerase II transcription subunit 19 [Pteropus alecto] |
|  |  | 82 | [ref\|NP_703151.2\|](https://www.ncbi.nlm.nih.gov/protein/952009246?report=genbank&log$=protalign&blast_rank=3&RID=UN5PAA0W016) | mediator of RNA polymerase II transcription subunit 19 isoform 2 [Homo sapiens] |
| 310 | Query=c40152/f1p0/1487 | 97 | ef\|XP_016876784.1\| | MAP/microtubule affinity-regulating kinase 3 isoform X8 [Homo sapiens] |
| 311 | Query=c40292/f1p3/2109 | 94 | [ref\|XP_016880852.1\|](https://www.ncbi.nlm.nih.gov/protein/1034602159?report=genbank&log$=protalign&blast_rank=1&RID=UNR7YT7U014) | PREDICTED: CDP-diacylglycerol--glycerol-3-phosphate 3-phosphatidyltransferase, mitochondrial isoform X6 [Homo sapiens] |
| 312 | Query=c40465/f1p1/1525 | 80 | [ref\|XP_016885242.1\|](https://www.ncbi.nlm.nih.gov/protein/1034675015?report=genbank&log$=protalign&blast_rank=4&RID=URS911B7014) | PREDICTED: spermine synthase isoform X1 [Homo sapiens] |
| 313 | Query=c41258/f1p104/1449 | 93 | [ref\|NP_003356.2\|](https://www.ncbi.nlm.nih.gov/protein/46593007?report=genbank&log$=protalign&blast_rank=1&RID=URUMUH72016) | cytochrome b-c1 complex subunit 1, mitochondrial precursor [Homo sapiens] |
| 314 | Query=c41501/f1p8/2243 | 92 | [ref\|NP_055205.2\|](https://www.ncbi.nlm.nih.gov/protein/77404397?report=genbank&log$=protalign&blast_rank=4&RID=URV07HHD01R) | staphylococcal nuclease domain-containing protein 1 [Homo sapiens] |
| 315 | Query=c41946/f2p2/1398 | 88 | [ref\|NP_000150.1\|](https://www.ncbi.nlm.nih.gov/protein/4503943?report=genbank&log$=protalign&blast_rank=2&RID=URVJC9UF014) | glutaryl-CoA dehydrogenase, mitochondrial isoform a precursor [Homo sapiens] |
| 316 | Query=c41972/f2p1/967 | 95 | [ref\|NP_002593.1\|](https://www.ncbi.nlm.nih.gov/protein/4505673?report=genbank&log$=protalign&blast_rank=1&RID=URW4H514016) | retinal rod rhodopsin-sensitive cGMP 3',5'-cyclic phosphodiesterase subunit gamma |
| 317 | Query=c4270/f1p1/1210 | 92 | [ref\|NP_001266285.1\|](https://www.ncbi.nlm.nih.gov/protein/525342559?report=genbank&log$=protalign&blast_rank=1&RID=URWNGEF401R) | acyl-protein thioesterase 1 isoform 2 [Homo sapiens] |
| 318 | Query=c4504/f4p32/879 | 97 | [ref\|NP_001616.1\|](https://www.ncbi.nlm.nih.gov/protein/4502013?report=genbank&log$=protalign&blast_rank=3&RID=URWUMZXS01R) | adenylate kinase 2, mitochondrial isoform a [Homo sapiens] |
| 319 | Query=c46533/f3p1/1440 | 96 | [ref\|XP_002807536.2\|](https://www.ncbi.nlm.nih.gov/protein/390473753?report=genbank&log$=protalign&blast_rank=6&RID=URX15PZR01R) | protein farnesyltransferase/geranylgeranyltransferase type-1 subunit alpha isoform X2 [Callithrix jacchus] |
|  |  | 90 | [ref\|NP_002018.1\|](https://www.ncbi.nlm.nih.gov/protein/4503771?report=genbank&log$=protalign&blast_rank=3&RID=URX15PZR01R) | protein f /geranylgeranyltransferase type-1 subunit alpha [Homo sapiens] |
| 320 | Query=c49191/f1p8/1702 | 93 | [ref\|NP_005872.2\|](https://www.ncbi.nlm.nih.gov/protein/171906589?report=genbank&log$=protalign&blast_rank=2&RID=US5XYZJX014) | [3-methyl-2-oxobutanoate dehydrogenase [lipoamide]] kinase, mitochondrial isoform a precursor [Homo sapiens] |
| 321 | Query=c50937/f2p2/801 | 99 | [ref\|XP_009426340.1\|](https://www.ncbi.nlm.nih.gov/protein/694956812?report=genbank&log$=protalign&blast_rank=1&RID=US76S3XH014) | PREDICTED: cytochrome c oxidase assembly protein COX16 homolog, mitochondrial isoform X1 [Pan troglodytes] |
|  |  | 73 | [ref\|NP_057552.1\|](https://www.ncbi.nlm.nih.gov/protein/7706011?report=genbank&log$=protalign&blast_rank=2&RID=US76S3XH014) | cytochrome c oxidase assembly protein COX16 homolog, mitochondrial isoform 1 precursor [Homo sapiens] |
| 322 | Query=c51273/f1p1/1956 | 98 | [ref\|NP_777572.2\|](https://www.ncbi.nlm.nih.gov/protein/195972892?report=genbank&log$=protalign&blast_rank=1&RID=US77VKKB016) | fatty-acid amide hydrolase 2 [Homo sapiens] |
| 323 | Query=c51945/f1p4/1475 | 92 | [ref\|NP_079431.1\|](https://www.ncbi.nlm.nih.gov/protein/13376751?report=genbank&log$=protalign&blast_rank=1&RID=US81UAKS014) | proteasomal ATPase-associated factor 1 isoform 2 [Homo sapiens] |
| 324 | Query=c5272/f2p0/1789 | 100 | [ref\|XP_003828299.1\|](https://www.ncbi.nlm.nih.gov/protein/397516150?report=genbank&log$=protalign&blast_rank=1&RID=US83F0SH014) | PREDICTED: DNA-directed RNA polymerase II subunit RPB4 isoform X1 [Pan paniscus] |
|  |  | 76 | [ref\|NP_004796.1\|](https://www.ncbi.nlm.nih.gov/protein/4758574?report=genbank&log$=protalign&blast_rank=4&RID=US83F0SH014) | DNA-directed RNA polymerase II subunit RPB4 [Homo sapiens] |
| 325 | Query=c53472/f3p10/1008 | 98 | [ref\|NP_060140.2\|](https://www.ncbi.nlm.nih.gov/protein/109148508?report=genbank&log$=protalign&blast_rank=2&RID=US995TKW014) | ubiquitin thioesterase OTUB1 [Homo sapiens] |
| 326 | Query=c5447/f1p4/2025 | 97 | [ref\|NP_001311228.1\|](https://www.ncbi.nlm.nih.gov/protein/1024249317?report=genbank&log$=protalign&blast_rank=1&RID=US9WYANU014) | dolichyl-diphosphooligosaccharide--protein glycosyltransferase subunit 2 isoform 3 precursor [Homo sapiens] |
| 327 | Query=c5524/f5p7/1068 | 99 | [ref\|XP_004060479.1\|](https://www.ncbi.nlm.nih.gov/protein/426388083?report=genbank&log$=protalign&blast_rank=1&RID=USA372VX014) | PREDICTED: cytochrome b-c1 complex subunit Rieske, mitochondrial-like isoform 2 [Gorilla gorilla gorilla] |
|  |  | 93 | [ref\|NP_005994.2\|](https://www.ncbi.nlm.nih.gov/protein/163644321?report=genbank&log$=protalign&blast_rank=4&RID=USA372VX014) | cytochrome b-c1 complex subunit Rieske, mitochondrial [Homo sapiens] |
| 328 | Query=c65158/f1p4/2998 | 95 | [ref\|NP_001293008.1\|](https://www.ncbi.nlm.nih.gov/protein/807045910?report=genbank&log$=protalign&blast_rank=4&RID=USAUH84X014) | CAD protein isoform 2 [Homo sapiens] |
| 329 | Query=c66208/f1p6/1141 | 93 | [ref\|NP_002927.2\|](https://www.ncbi.nlm.nih.gov/protein/21359816?report=genbank&log$=protalign&blast_rank=1&RID=USAVZRRK014) | ribonuclease H1 isoform 1 precursor [Homo sapiens] |
| 330 | Query=c6654/f1p2/1285 | 88 | [gb\|AAH34360.1\|](https://www.ncbi.nlm.nih.gov/protein/21706438?report=genbank&log$=protalign&blast_rank=1&RID=USB2VUWP014) | Citrate lyase beta like [Homo sapiens] |
| 331 | Query=c70822/f2p5/1035 | 91 | [ref\|NP_006212.1\|](https://www.ncbi.nlm.nih.gov/protein/5453898?report=genbank&log$=protalign&blast_rank=2&RID=USBZXUHT014) | peptidyl-prolyl cis-trans isomerase NIMA-interacting 1 [Homo sapiens] |
| 332 | Query=c70937/f1p10/1128 | 90 | [ref\|NP_001344.2\|](https://www.ncbi.nlm.nih.gov/protein/5453543?report=genbank&log$=protalign&blast_rank=2&RID=USC2KZY5016) | aldo-keto reductase family 1 member C1 [Homo sapiens] |
| 333 | Query=c145087/f1p4/2232 | 97 | ref\|XP_005247011.1\| | E3 ubiquitin-protein ligase TRIP12 isoform X1 [Homo sapiens] |
| 334 | Query=c147917/f1p0/1852 | 98 | ref\|NP_001193762.1\| | histone acetyltransferase KAT5 isoform 4 [Homo sapiens] |
| 335 | Query=c148726/f1p9/6101 | 95 | [ref\|NP_079055.3\|](https://www.ncbi.nlm.nih.gov/protein/226371737?report=genbank&log$=protalign&blast_rank=1&RID=VMRK35KC01R) | phosphatidylinositol 5-phosphate 4-kinase type-2 gamma isoform a [Homo sapiens] |
| 336 | Query=c15810/f1p1/814 | 98 | [gb\|AAH46205.1\|](https://www.ncbi.nlm.nih.gov/protein/28374296?report=genbank&log$=protalign&blast_rank=3&RID=VNACB31G01R) | USP9X protein, partial [Homo sapiens] |
| 337 | Query=c26715/f1p6/2577 | 91 | ref\|NP_036452.1 | group XV phospholipase A2 precursor [Homo sapiens] |
| 338 | Query=c27361/f1p1/841 | 85 | [ref\|XP_016879092.1\|](https://www.ncbi.nlm.nih.gov/protein/1034595777?report=genbank&log$=protalign&blast_rank=2&RID=VRFVXPN6014) | sulfotransferase 1A2 isoform X3 [Homo sapiens] |
| 339 | Query=c9936/f1p6/2686 | 86 | [ref\|NP_076977.3\|](https://www.ncbi.nlm.nih.gov/protein/51094101?report=genbank&log$=protalign&blast_rank=10&RID=VRHRXYZ901R) | ATP-dependent RNA helicase DDX54 isoform 2 [Homo sapiens] |
| 340 | Query=c9998/f1p6/1939 | 97 | [gb\|AAH26039.1\|](https://www.ncbi.nlm.nih.gov/protein/45708796?report=genbank&log$=protalign&blast_rank=2&RID=VRHYH44N014) | Mitochondrial GTPase 1 homolog (S. cerevisiae) [Homo sapiens] |

**The detail of a hormone metabolic enzyme in HepG2 cells：**

**Table S11. The mutation of 3*β*-hydroxysteroid dehydrogenase**

| **cDNA** | **Identity%** | **Sequence ID(Ref.)** | **Predicted Protein** |
| --- | --- | --- | --- |
| Query=c165854/f1p2/2007 | 86 | NP_079469.2 | 3 beta-hydroxysteroid dehydrogenase type 7 isoform a [Homo sapiens] |

**The amino acid sequence of 3*β*-hydroxysteroid dehydrogenase was compared with the NCBI database:**

Query=c165854/f1p2/2007

MADSAQAQKLVYLVTGGCGFLGEHVVRMLLQREPRLGELRVFDQHLGPWLEELKTGTRNVIEACVQTGTRFLVYTSSMEVVGPNTKGHPFYRGNEDTPYEAVHRHPYPCSKALAEWLVLEANGRKVRGGLPLVTCALRPTGIYGEGHQIMRDFYRQGLRLGGWLFRAIPASVEHGRVYVGNVAWMHVlaareleqraalMGGQVYFCYDGSPYRSYEDFNMEFLGPCGLRLVGARPLLPYWllvflaalnallqwllrplvlyapllNPYTLAVANTTFTVSTDKAQRHFGYEPLFSWEDSRTRTILWVQAATGSAQ

**3 beta-hydroxysteroid dehydrogenase type 7 isoform a [Homo sapiens]**

**Sequence ID:**[**NP_079469.2**](https://www.ncbi.nlm.nih.gov/protein/19923621?report=genbank&log$=protalign&blast_rank=3&RID=0B4CAZ0101R)**Length: 369Number of Matches: 1**

Related Information

[Gene](https://www.ncbi.nlm.nih.gov/gene?term=19923621%5bPUID%5d%20OR%20767988827%5bPUID%5d%20OR%20767988829%5bPUID%5d%20OR%20767988831%5bPUID%5d%20OR%2047605550%5bPUID%5d%20OR%2013436260%5bPUID%5d%20OR%20157929146%5bPUID%5d%20OR%20157929148%5bPUID%5d&RID=0B4CAZ0101R&log$=genealign&blast_rank=3)-associated gene details

Range 1: 1 to 369[GenPept](https://www.ncbi.nlm.nih.gov/protein/19923621?report=genbank&log$=protalign&blast_rank=3&RID=0B4CAZ0101R&from=1&to=369)[Graphics](https://www.ncbi.nlm.nih.gov/protein/19923621?report=graph&rid=0B4CAZ0101R%5b19923621%5d&tracks=%5bkey:sequence_track,name:Sequence,display_name:Sequence,id:STD1,category:Sequence,annots:Sequence,ShowLabel:true%5d%5bkey:gene_model_track,CDSProductFeats:false%5d%5bkey:alignment_track,name:other%20alignments,annots:NG%20Alignments|Refseq%20Alignments|Gnomon%20Alignments|Unnamed,shown:false%5d&v=0:387&appname=ncbiblast&link_loc=fromHSP)Next MatchPrevious Match

**Table S12. Alignment statistics for match #1**

| **Score** | **Expect** | **Method** | **Identities** | **Positives** | **Gaps** |
| --- | --- | --- | --- | --- | --- |
| 629bits(1621) | 0.0 | Compositional matrix adjust. | 316/369(86%) | 316/369(85%) | 52/369(14%) |

Query 1 MADSAQAQKLVYLVTGGCGFLGEHVVRMLLQREPRLGELRVFDQHLGPWLEELKTG---- 56

MADSAQAQKLVYLVTGGCGFLGEHVVRMLLQREPRLGELRVFDQHLGPWLEELKTG

Sbjct 1 MADSAQAQKLVYLVTGGCGFLGEHVVRMLLQREPRLGELRVFDQHLGPWLEELKTGPVRV 60

Query 57 ------------------------------------------------TRNVIEACVQTG 68

TRNVIEACVQTG

Sbjct 61 TAIQGDVTQAHEVAAAVAGAHVVIHTAGLVDVFGRASPKTIHEVNVQGTRNVIEACVQTG 120

Query 69 TRFLVYTSSMEVVGPNTKGHPFYRGNEDTPYEAVHRHPYPCSKALAEWLVLEANGRKVRG 128

TRFLVYTSSMEVVGPNTKGHPFYRGNEDTPYEAVHRHPYPCSKALAEWLVLEANGRKVRG

Sbjct 121 TRFLVYTSSMEVVGPNTKGHPFYRGNEDTPYEAVHRHPYPCSKALAEWLVLEANGRKVRG 180

Query 129 GLPLVTCALRPTGIYGEGHQIMRDFYRQGLRLGGWLFRAIPASVEHGRVYVGNVAWMHVL 188

GLPLVTCALRPTGIYGEGHQIMRDFYRQGLRLGGWLFRAIPASVEHGRVYVGNVAWMHVL

Sbjct 181 GLPLVTCALRPTGIYGEGHQIMRDFYRQGLRLGGWLFRAIPASVEHGRVYVGNVAWMHVL 240

Query 189 AARELEQRAALMGGQVYFCYDGSPYRSYEDFNMEFLGPCGLRLVGARPLLPYWLLVFLAA 248

AARELEQRA LMGGQVYFCYDGSPYRSYEDFNMEFLGPCGLRLVGARPLLPYWLLVFLAA

Sbjct 241 AARELEQRATLMGGQVYFCYDGSPYRSYEDFNMEFLGPCGLRLVGARPLLPYWLLVFLAA 300

Query 249 LNALLQWLLRPLVLYAPLLNPYTLAVANTTFTVSTDKAQRHFGYEPLFSWEDSRTRTILW 308

LNALLQWLLRPLVLYAPLLNPYTLAVANTTFTVSTDKAQRHFGYEPLFSWEDSRTRTILW

Sbjct 301 LNALLQWLLRPLVLYAPLLNPYTLAVANTTFTVSTDKAQRHFGYEPLFSWEDSRTRTILW 360

Query 309 VQAATGSAQ 317

VQAATGSAQ

Sbjct 361 VQAATGSAQ 369

**
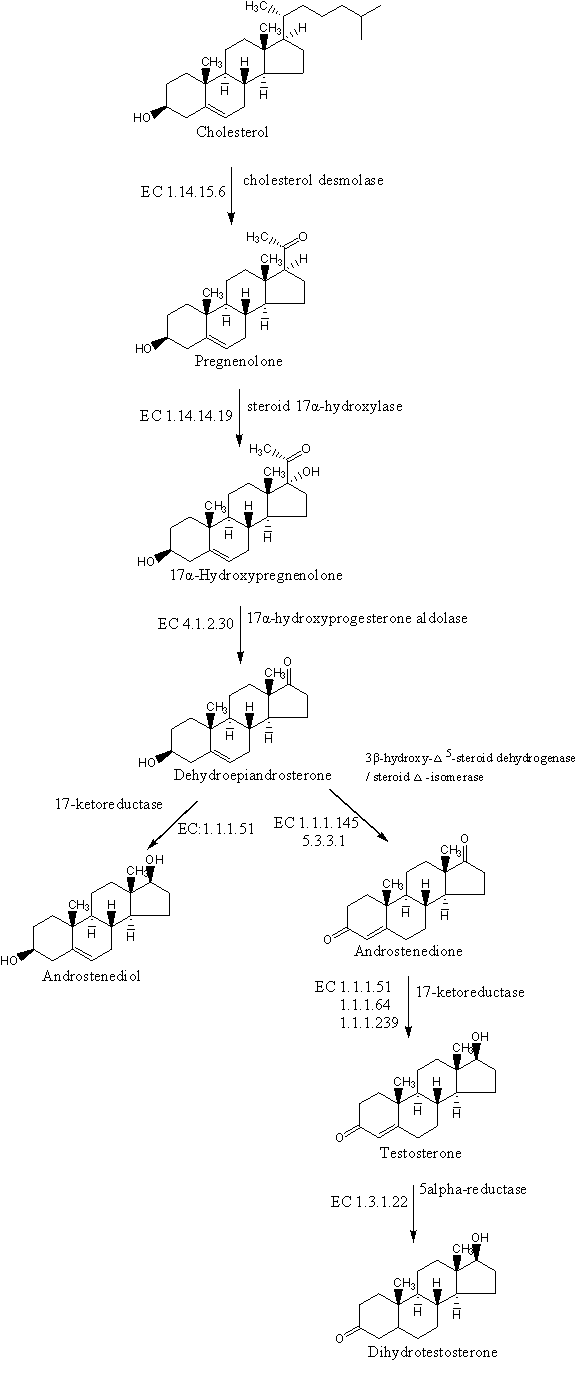
This study refers to human hormone metabolism pathway**

**Figure S****4.** The metabolic pathway of androgen hormone


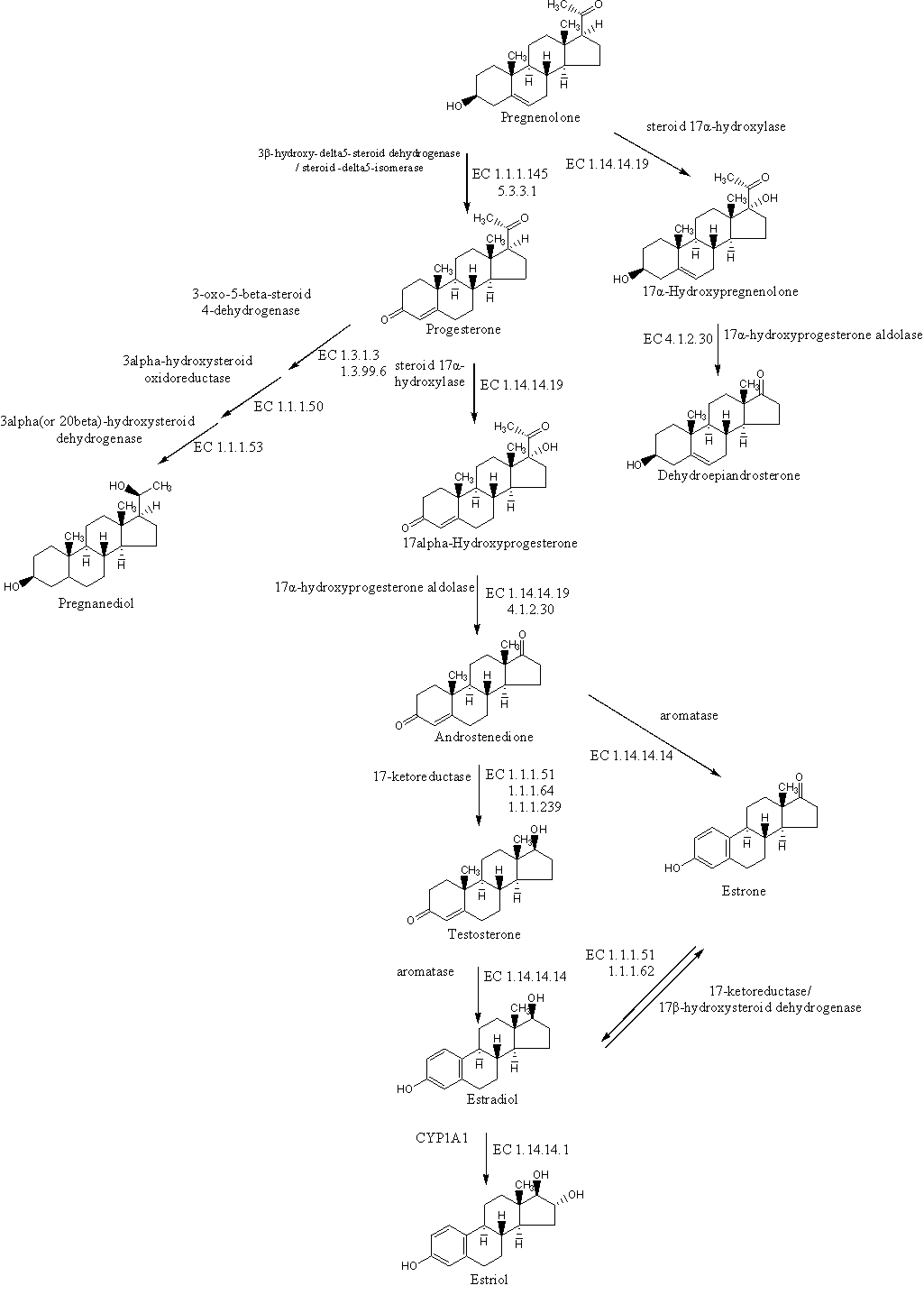


**Figure S5**. The metabolic pathway of estrogen hormone
